# Forchlorfenuron and Novel Analogs Cause Cytotoxic Effects in Untreated and Cisplatin-Resistant Malignant Mesothelioma-Derived Cells

**DOI:** 10.3390/ijms23073963

**Published:** 2022-04-02

**Authors:** Thomas Henzi, Kim-Long Diep, Anne Oberson, Valerie Salicio, Christian G. Bochet, Beat Schwaller

**Affiliations:** 1 Anatomy, Section of Medicine, Faculty of Science and Medicine, University of Fribourg, Route Albert-Gockel 1, CH-1700 Fribourg, Switzerland; thomas.henzi@unifr.ch (T.H.); anne.oberson@unifr.ch (A.O.); valerie.salicio@unifr.ch (V.S.); 2 Department of Chemistry, Faculty of Science and Medicine, University of Fribourg, Chemin du Musee 9, CH-1700 Fribourg, Switzerland; kim-long.diep@unifr.ch (K.-L.D.); christian.bochet@unifr.ch (C.G.B.)

**Keywords:** malignant mesothelioma, septin 7, forchlorfenuron, FCF, cisplatin, septin cytoskeleton, combination therapy

## Abstract

Malignant mesothelioma (MM) is a currently incurable, aggressive cancer derived from mesothelial cells, most often resulting from asbestos exposure. The current first-line treatment in unresectable MM is cisplatin/pemetrexed, which shows very little long-term effectiveness, necessitating research for novel therapeutic interventions. The existing chemotherapies often act on the cytoskeleton, including actin filaments and microtubules, but recent advances indicate the ‘fourth’ form consisting of the family of septins, representing a novel target. The septin inhibitor forchlorfenuron (FCF) and FCF analogs inhibit MM cell growth in vitro, but at concentrations which are too high for clinical applications. Based on the reported requirement of the chloride group in the 2-position of the pyridine ring of FCF for MM cell growth inhibition and cytotoxicity, we systematically investigated the importance (cell growth-inhibiting capacity) of the halogen atoms fluorine, chlorine, bromine and iodine in the 2- or 3-position of the pyridine ring. The MM cell lines ZL55, MSTO-211H, and SPC212, and—as a control—immortalized Met-5A mesothelial cells were used. The potency of the various halogen substitutions in FCF was mostly correlated with the atom size (covalent radius); the small fluoride analogs showed the least effect, while the largest one (iodide) most strongly decreased the MTT signals, in particular in MM cells derived from epithelioid MM. In the latter, the strongest effects in vitro were exerted by the 2-iodo and, unexpectedly, the 2-trifluoromethyl (2-CF_3_) FCF analogs, which were further tested in vivo in mice. However, FCF-2-I and, more strongly, FCF-2-CF_3_ caused rapidly occurring strong symptoms of systemic toxicity at doses lower than those previously obtained with FCF. Thus, we investigated the effectiveness of FCF (and selected analogs) in vitro in MM cells which were first exposed to cisplatin. The slowly appearing population of cisplatin-resistant cells was still susceptible to the growth-inhibiting/cytotoxic effect of FCF and its analogs, indicating that cisplatin and FCF target non-converging pathways in MM cells. Thus, a combination therapy of cisplatin and FCF (analogs) might represent a new avenue for the treatment of repopulating chemo-resistant MM cells in this currently untreatable cancer.

## 1. Introduction

Malignant mesothelioma (MM) is a currently incurable, aggressive cancer derived from the mesothelial cells that cover the walls of body cavities—including the pericardium, pleura and the peritoneum—as well as the surfaces of the organs localized within those cavities. MM etiology is most strongly associated with asbestos exposure [1]. The incidence rate is around one case per 100,000 persons, leading to approximately 3000 annual cases in the US [2]; it is estimated that more than 100,000 individuals worldwide die each year from MM, lung cancer and asbestosis [3]. Up to now, all of the treatment modalities against MM have shown rather little effectiveness. The median survival of MM patients is in the order of 12–18 months, and the 5-year survival rate is less than 5% [4]. At present, the only FDA—and European Medicines Agency—approved first-line treatment in unresectable MM is cisplatin-pemetrexed, generally consisting of four cycles [5]. While the initial treatment leads to substantial tumor regression, a rather rapid relapse of the tumor generally occurs due to the growth of chemoresistant MM cells. These resistant cell populations—also termed MM-initiating cells [6], MM side population [7] or ALDH^high^CD44^+^ MM cells [8]—show various traits of cancer stem cells (CSC; in the case of MM these are also named mesothelioma-derived stem cells (MSC)), which have also previously been demonstrated to exist in MM cell populations [9,10]. Such MSC are also assumed to be responsible for cancer cell repopulation between the intervals of chemo-radiation in MM treatment, as was recently evidenced in a mouse model [11].

**Figure 1:**
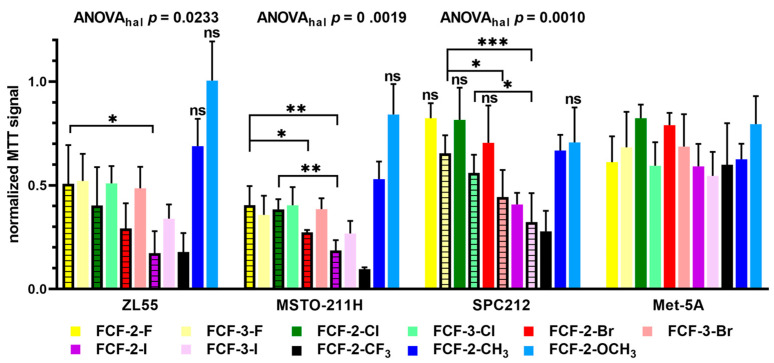
Cell proliferation/viability-decreasing effect of FCF and 10 FCF analogs in human MM cells (ZL55, MSTO-211H, SPC212) and immortalized (untransformed) Met-5A mesothelial cells. The cells were exposed to FCF and 10 analogs (for nomenclature, see Supplementary Information) at a concentration of 50 µM, and MTT assays were performed 96 h after the beginning of the treatment. The bars represent the mean (± SD) of 4–5 independent experiments, and each condition was measured in three wells. In each independent experiment, the MTT signal of the untreated cells (medium containing 0.125% DMSO) of each cell line was set to 1.0. For the ZL55 and MSTO-211H cells, the bars for the FCF analogs with halogen atoms in the 2-position of the pyridine ring are stippled; for SPC212 cells, the stippled bars represent the halogenated FCF analogs with modifications in the 3-position. In all of the MM cell lines (but not in untransformed Met-5A) the ANOVA of all of the experimental conditions including the control cells revealed significant differences (*p* < 0.0001) among the compounds. Pairwise comparisons (control vs. FCF compounds) showed significant decreases (Supplementary Table S1), except where they are marked as not significant (n.s.). The grouping of the four single-halide compounds in the 2-position (ZL55 and MSTO-211H) or the 3-position (SPC212) revealed significant differences (ANOVA_hal_) within the group; significant differences between pairs are marked by asterisks. *, ** and *** represent *p* < 0.05, *p* < 0.01 and *p* < 0.001, respectively.

Other adjuvant or neoadjuvant therapies currently being tested in phase I–III clinical trials include viral therapy, the application of neo-antigen-based vaccines or angiogenesis inhibitors, immune checkpoint blockade—e.g., aiming at programmed death 1 (PD-1) and programmed death ligand-1 (PD-L1)—or therapies targeting mesothelin, e.g., with antibody drug conjugates (for details, see Figure 1 and Table 1 in [4]). Novel substances targeting pathways in which tumor suppressor genes are implicated in MM etiology, such as *BAP1*, *NF2* or *CDKN2A* encoding BRCA1-associated protein 1, neurofibromin 2 (merlin) and cyclin-dependent kinase inhibitor 2A, respectively, together with inhibitors of CSC survival (e.g., focal adhesion kinase (FAK) inhibitors) are other promising avenues towards novel MM treatments (for more details, see [2,4,12]). Furthermore, surgery for malignant pleural mesothelioma after radiotherapy (SMART) is still being investigated as a promising MM treatment option [13].

While chemotherapies often act on ‘classical’ cytoskeletal components of rapidly proliferating tumor cells including actin filaments and microtubules, much less has been investigated regarding the possibility of targeting the sometimes called ‘fourth’ cytoskeletal component consisting of septins, in mammals embracing 13 septin genes subdivided into four distinct groups [14]. Septin family members associate to form hetero-oligomers in the form of higher-order cytoskeletal structures, including fibers, filaments and ring structures. Besides their important function in cytokinesis, septins are implicated in many other cellular processes, including cell proliferation, migration, polarity and morphology, as well as cell signaling and apoptosis. Because these functions are altered in the process of carcinogenesis, the targeting of septin function(s) is being discussed as a putative novel cancer therapy [15]. This may include the downregulation of specific septin family members, or the blocking of septin assembly/function by small molecule inhibitors. Such an inhibitor exists in the form of the diarylurea-based forchlorfenuron (FCF), which was initially identified as a molecule that inhibits cell division in budding yeast and induces ectopic septin structures by stabilizing septin heteromers [16]. Several reported effects of FCF are linked to the impairment of septin distribution, assembly or dynamics [17], or septin/protein interactions (e.g., ErbB2/HER2; [18]), as nearly identical results were obtained by septin depletion/downregulation. FCF also decreases the proliferation/viability and alters the morphology of MM cells (increase in spindle-like cells), similarly to the effect observed in cells, where septin 7 is downregulated by *SEPT7* shRNA [19]. However, the impairment of the migration of epithelial cells by FCF was shown to be independent of septin function, and is caused by altered cell signaling resulting from the decreased expression of c-Jun and attenuated ERK activity [20]. Additional septin-independent effects of FCF have been reported [21].

Currently, only two studies have investigated, in a systematic way, the application of FCF and—moreover—FCF analogs for the inhibition of tumor cell growth in vitro using cell lines from different organs and tissues [19,22]. FCF was initially identified as a phytohormone with cytokinin activity, and is used as a plant growth regulator to boost fruit size [23,24]. More recently, FCF was shown to efficiently inhibit the growth of tumor cells derived from various organs and tissues [25,26]. FCF’s low toxicity, as reported by the U.S. Environmental Protection Agency (EPA), makes FCF (and, putatively, FCF analogs) promising candidates for future therapeutic applications in various cancer types [19,22]. 

The effects of FCF and its analogs in cancer cells are several-fold: MM cells are blocked in the G2/M phase of the cell cycle and, furthermore, apoptosis is increased [19]; the latter effect is also observed in FCF analog-treated endometrial ECC-1 cancer cells [22]. The study by Blum et al. demonstrated the importance (and ultimate necessity) of the chloro group in the 2-position of the pyridine ring of FCF for protein (septin) interaction; all three FCF analogs lacking the chloro group showed no anti-proliferative/cytotoxic effect in the MM cell lines ZL55, MSTO-211H and SPC212, or in untransformed, immortalized Met-5A mesothelial cells [19]. The importance of the 2-chloropyridine part of FCF was also confirmed by others [22]. In the pharmaceutical (drugs) and agrochemical (pesticides, disinfectants) industries, the halogenation of active compounds has long been exploited with the aim to tailor these molecules to their specific needs; e.g., to increase their metabolic stability, affinity for the target molecule, or lipophilicity. A search in the protein database (PDB) of halogenated ligands co-crystallized with proteins led to the discovery of some novel principles revealing the electrostatic (polar) and hydrophobic nature of such halogen/protein interactions [27]. In general, halogens in organic halides show a preference for hydrophobic amino acids (leucine, glycine, phenylalanine) and the hydrogen-bonding residues serine and threonine. However, additional preferences exist for each halide for particular amino acids [27]; see also the Discussion section.

In this structure–activity relationship study carried out in MM cells, in a systematic approach, we further investigated the antiproliferative/cytotoxic effect of newly synthesized FCF analogs containing different halogens (F, Cl, Br, I), as well as other substituents in the 2- or 3-position of the pyridine ring. The potency of the FCF halide analogs was correlated with their atom size—i.e., the covalent radius—of halogens (F < Cl < Br < I), an effect which was most clearly observed with analogs containing the various halogens in the 2-position in epithelioid MM cells (MSTO-211H and ZL55), and in the 3-position in SPC212 cells with spindloid (sarcomatoid) morphology.

Because the FCF analogs with a trifluoromethyl (CF_3_) or an iodo group in the 2-position (FCF-2-CF_3_ and FCF-2-I) showed the highest potency in all of the tested MM cell lines in vitro, these two compounds were also tested in vivo in mice. However, treatment with either compound resulted in rapid and strong systemic toxicity. Thus, as an alternate strategy to efficiently block MM cell proliferation/viability, we tested a combination therapy consisting of initial cisplatin treatment followed by the co-addition of FCF (and analogs) in MM cells in vitro. In all cases, the emergent populations of cisplatin-resistant MM were still susceptible to the inhibiting effects of FCF (and its analogs).

## 2. Results

### 2.1. Exposure to FCF and FCF Analogs Decreases the Proliferation/Viability of Cells of Mesothelial Origin

In our previous study [19], we showed that FCF inhibits the cell proliferation/viability of several MM cell lines (eight human, two mouse) in vitro, in a concentration-dependent manner (IC_50_ values: ~20–60 µM). Importantly, MM cell lines are more strongly affected than the non-transformed immortalized mesothelial cell lines Met-5A and LP9. Here, we selected three representative MM cell lines based on our prior results. These included: (I) ZL55 cells derived from an epithelioid MM characterized by nearly 100% epithelioid cells; (II) the commonly-used cell line MSTO-211H, generated from a biphasic MM and showing mostly epithelioid morphology and characteristics in vitro, with rare spindle-shaped cells; and III) SPC212 cells obtained from a sarcomatoid MM and, when cultured in vitro, consisting of a mixture of a few epithelioid-like and predominantly fibroblast-like (sarcomatoid) cells. As in our previous study [19], Met-5A cells showing higher resistance to the growth-inhibiting/cytotoxic effects of FCF evidenced by a higher IC_50_ value served as an example of untransformed, immortalized mesothelial cells.

The testing of several FCF analogs previously revealed the importance of the chloro group in position 2 in the pyridine ring of FCF [19]. The growth-inhibitory effect on MM cells was essentially abolished when applying FCF analogs lacking the chloro group (see Figure 6 in [19]). As we reasoned that the electronegative group at this position was indispensable, we synthesized novel FCF analogs containing other halogen atoms (F, Br, I) at either position 2 or 3 of the pyridine ring, in order to further explore the importance and conceivable selectivity brought about by the chloro group in FCF (Supplementary Table S1). FCF analogs, including one with a 2-CF_3_ group, were tested in the MTT assay using identical conditions, as reported previously [19]. Additionally, FCF analogs with non-halogen substituents in the 2-position of the pyridine ring were tested: 2-methyl (2-CH_3_) and 2-methoxy (2-OCH_3_) analogs of FCF (Supplementary Table S1). 

A concentration of 50 µM for FCF and all of the FCF analogs was chosen for the initial experiments, as IC_50_ values in that range have been reported for the parental compound FCF in most MM cells [19], in line with our aim of finding more potent FCF analogs. As FCF and its analogs were dissolved in DMSO, the control (untreated) cells of the four lines were also grown in the presence of the same final DMSO concentration during the experiment (≤0.125%). The MTT values of the untreated cells at 96 h were defined as 1.0. In line with previous results [19], the MTT signals and growth curves (e.g., for MSTO-211H cells) were not different in the presence or absence of 0.125% DMSO in the cell culture medium (data not shown).

At first sight, it is noticeable that the profiles (normalized MTT signals obtained after 96 h of treatment) of all 11 tested substances (FCF and 10 analogs) were rather similar in the mostly epithelioid ZL55 and MSTO-211H cells (Figure 1). Globally, MSTO-211H cells showed a slightly higher sensitivity (lower MTT signals) towards the effects of the FCF analogs than the ZL55 cells. Compared to the untreated cells, the MTT signals were lower in ZL55 and MSTO-211 cells treated with FCF and its analogs, as confirmed by the ANOVA and post-hoc analyses (Supplementary Table S2). The decreases were significant with all compounds, with the exception of the FCF-2-OCH_3_ analog in both cell lines and additionally FCF-2-CH_3_ in ZL55 cells. With the selected concentration of 50 µM, significant decreases were obtained with all of the halogenated FCF analogs and the one containing a trifluoromethyl (CF_3_) group in the 2-position.

Qualitatively, the effects were rather similar (yet smaller) for most of the compounds in SPC212 cells, with few exceptions. Whilst, in ZL55 and MSTO-211H cells, FCF analogs with the chloro, bromo and iodo group in the 2-position had a stronger effect than the same substituents in the 3-position, the opposite was true in SPC212 cells. The 3-F, 3-Cl and 3-Br analogs—and to a lesser extent the 3-I analog—were more potent (Figure 1). Possible reasons for this difference between epithelioid and spindle-shaped MM cells are addressed in the Discussion section. Moreover, the overall effect of all of the FCF analogs was weaker in SPC212 cells, as is consistent with our previous findings, i.e., a higher IC_50_ value for FCF in SPC212 cells compared to ZL55 and MSTO-211H cells (Figure 1C in [19]). In Met-5A mesothelial cells, the growth-inhibiting effects of FCF and its analogs were visibly smaller (and insignificant compared to untreated cells) than in the three MM cell lines, and the electron-withdrawing effect of the halogen vs. the 2-CH_3_ and 2-OCH_3_ groups was negligible.

A more detailed analysis on the effect of the different halogen atoms in the tested FCF analogs allowed for further generalizations that are mostly based on comparisons of the results obtained in ZL55 and MSTO-211H cells. FCF analogs without an electron-withdrawing group—that is, the 2-CH_3_ and, even more so, the 2-OCH_3_ analog—had a smaller impact on the proliferation/viability than all of the others containing halogen atoms. In particular, the larger methoxy group had essentially no effect on proliferation/viability indicating that the methoxy group prevented the rest of the molecule from binding to the in silicio-modeled FCF binding pocket present in septins (for details, see the Discussion and [28]). Significant differences in the potency of the various halogen substitutions in the pyridine ring of FCF were confirmed by ANOVA (ANOVA_hal_) followed by post-hoc analysis, and these were mostly correlated with atom size (covalent radius); the small fluoride analogs showed the least effect, while the largest one (iodide) most strongly decreased the MTT signals if the halogen atom was at the 2-position (pairwise comparisons by post-hoc analyses are shown in Figure 1).

In SPC212 cells, a clear halide size-dependent decrease in MTT signals was unexpectedly observed only with the 3-position analogs, as demonstrated by ANOVA followed by pair-wise comparisons (striped bars in Figure 1). Moreover, the effects were stronger than with the 2-position analogs for all four halides. As for ZL55 and MSTO-211H cells, both iodide analogs (2-I and 3-I) were the most effective ones in SPC212 cells. However, the strongest effect of all of the FCF analogs in all three MM cell lines was exerted by the one containing a trifluoromethyl (-CF_3_) group in the 2-position. In summary, an electronegative substituent in either the 2- (for epithelioid MM cells) or 3-position (for sarcomatoid MM cells) of the pyridine ring of FCF is essential for the inhibition of cell proliferation/viability; in agreement with previous results [19], the effects of FCF and its analogs were generally weaker in SPC212 cells with spindle-shaped morphology than in epithelioid MM cells.

### 2.2. Dose–Response Curves of Cells of Mesothelial Origin Exposed to FCF Analogs

**Figure 2:**
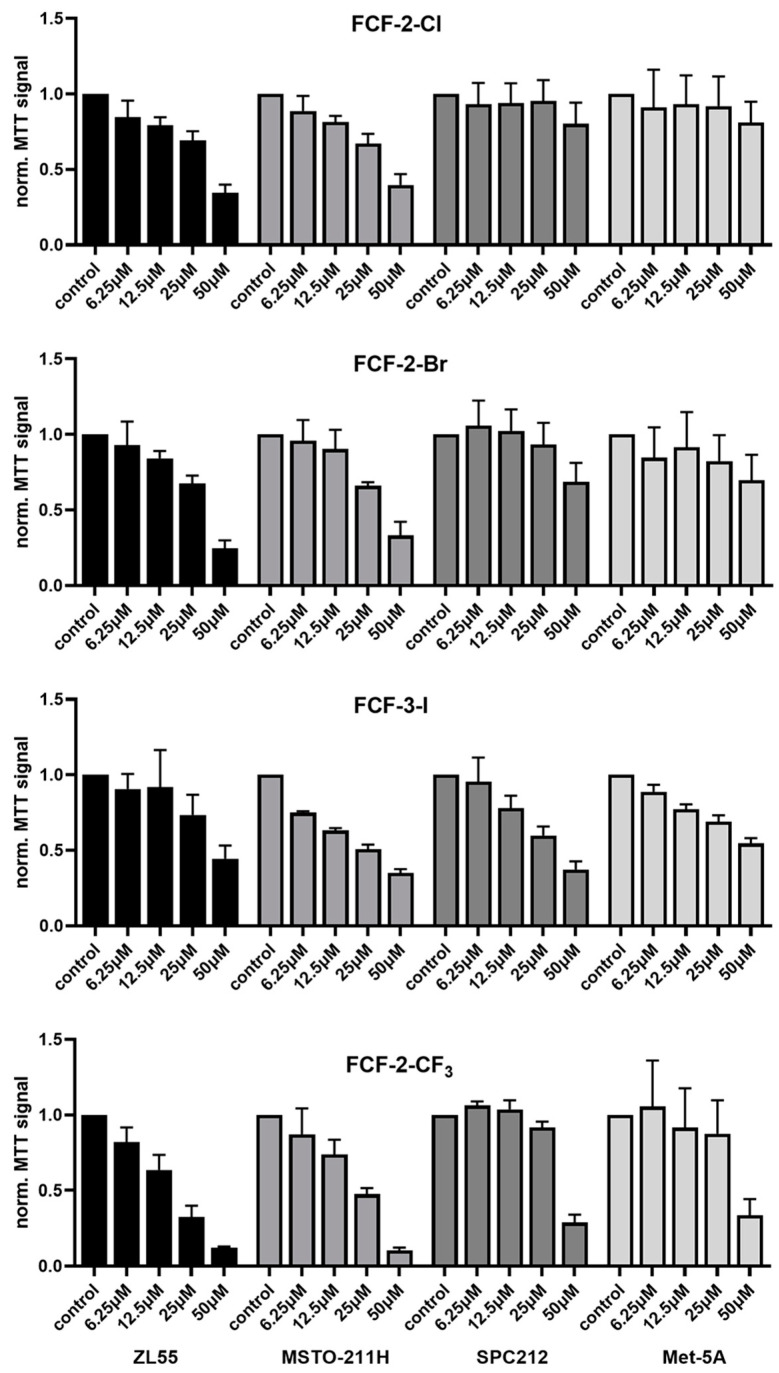
Dose–response curves obtained with (from left to right) ZL55, MSTO-211H, SPC212 and Met-5A cells treated with FCF-2-Cl (parental FCF), FCF-2-Br, FCF-3-I and FCF-2-CF_3_ for 96 h. MTT signals (normalized to non-treated cells from the control) of cells exposed to 6.25, 12.5, 25 and 50 µM FCF (and three FCF analogs). The results are the average of 3–4 independent experiments (mean ± SD); each condition was tested in three wells in each experiment. The results from the more sensitive ZL55 and MSTO-211H cells were further used to estimate the IC_50_ values (Supplementary Figures S1 and S2).

Based on the main intention to identify and characterize more potent FCF analogs with respect to the inhibition of MM cell function in vitro, further experiments were carried out with the three FCF analogs FCF-2-Br, FCF-3-I and FCF-2-CF_3_, and compared to the parental FCF (FCF-2-Cl). A dose–response curve was determined at the same time point (96 h) in ZL55, MSTO-211H, SPC212 and Met-5A cells with FCF (and its analogs) at concentrations of 6.25, 12.5, 25 and 50 µM (Figure 2). As is consistent with the results shown in Figure 1, the results obtained with ZL55 and MSTO-211H cells were nearly identical in this series of experiments. An inhibitory effect, i.e., a decrease in MTT signal intensity was observed in ZL55 and MSTO-211H cells at the lowest concentration (6.25 µM) with all four substances, while in SPC212 and Met-5A cells, higher concentrations of FCF (and its analogs) were required to decrease the MTT signals. The effect of FCF-2-Br was nearly identical to that of FCF (FCF-2-Cl) in all four cell lines, i.e., a concentration-dependent decrease in ZL55 and MSTO-211H cells, while only mildly affecting SPC212 and Met-5A cells. In SPC212 cells, the only promising candidate was FCF-3-I, showing a clear concentration-dependent decrease in MTT signal intensity. However, this was then relativized by a quite similar decrease in non-transformed Met-5A cells. This suggested that the effect was probably not specific for tumor cells. Treatment with FCF-2-CF_3_ resulted in a typical dose–response curve in ZL55 and MSTO-211H cells; in the other 2 cell lines the effect was almost binary: there was no decrease in the MTT signal intensity up to 25 µM, then an almost complete inhibition of proliferation (and a decrease in viability) at 50 µM.

The analyses of four data points per substance allowed for an approximate estimation of the IC_50_ values in ZL55 and MSTO-211H cells (Supplementary Figures S1 and S2). The concentrations of FCF and the three analogs for MSTO-211H cells required for half-maximal inhibition (IC_50_ values) were in a rather narrow range from the least potent—FCF-2-Cl (43 µM), followed by FCF 2-Br (37 µM) and FCF 3-I (31 µM)—to the most powerful, FCF-2-CF_3_ (20 µM), with the latter differing only by a factor of approximately two from FCF-2-Cl. Very similar results held true for ZL55 cells, where the IC_50_ value for FCF-2-CF_3_ (14 µM) was about threefold lower than that for the parental FCF (42 µM). In the less sensitive SPC212 cells, only the IC_50_ value estimates for the most potent FCF-3-I and FCF-2-CF_3_ were determined, and were in the order of 40 µM (Supplementary Figure S3).

In summary, when combining results from MSTO-211H and ZL55 cells, the inhibitory effects of FCF-3-I and FCF-2-Br were not significantly different from FCF. As the IC_50_ for FCF-2-CF_3_ was significantly lower (approximately two- to threefold) in the above cell lines, and because FCF-2-I (50 µM) showed similar potency to FCF-2-CF_3_ in MSTO-211H and ZL55 cells (Figure 1), these two substances were further tested in pilot experiments in vivo.

### 2.3. Systemic Toxicity of FCF-2-I and FCF-2-CF_3_ Observed in Mice In Vivo

Because FCF is an EPA-approved fertilizer used in fruit horticulture, its systemic effects in laboratory animals (mostly rats) have been investigated in greater detail; it was concluded that FCF shows low systemic toxicity and carcinogenicity in vivo (Pesticide Fact Sheet, EPA, 2004). In our previous study, we reported that FCF injected into the peritoneal cavity of BALB/c mice at a dose of 2.5 mg per mouse leads to typical cytostatic treatment-associated symptoms, including partial alopecia and heavy constipation; however, these symptoms were not observed until several days after the FCF treatment [19]. In addition, a few mice were found in transient hypothermia. At the histological level, the mesothelial cells forming the barrier between the peritoneal cavity and the layer of skeletal muscle of the diaphragm—in addition to the mesothelial cells of the tunica serosa of the intestine—had a more cuboidal appearance, which is characteristic for reactive mesothelial cells. Moreover, the rapidly proliferating epithelial cells at the base of the Lieberkuhn crypts including stem cells and Paneth cells had an atypical morphology, i.e., flatter (compressed) nuclei and an increased density of secretory granules (for details, see Figure 4 in [19]).

As only FCF-2-CF_3_ and FCF-2-I were more potent than FCF in the in vitro experiments in MM cells (Figure 1 and Figure 2, Supplementary Figure S1), and because there are, as yet, no data available on the potential toxicity of FCF-2-CF_3_ and FCF-2-I in vivo, pilot experiments were carried out with groups (group size: four or five mice, see Materials and Methods) of BALB/c mice (three treatment groups, and one control group per substance). The control mice injected with the vehicle (PG/DMSO) showed no symptoms during the entire testing period (8 days). The mice treated with 2.5 mg FCF-2-CF_3_ showed strong symptoms of systemic toxicity, i.e., no physical activity, no social interaction, no exploring just 1 h after the injection. Additional symptoms appeared during the next hours, and included trembling, staggering, forming a hunchback, and the development of shaggy fur. All of the animals in this group had to be sacrificed 6 h after the start of the experiment (the results from all of the in vivo experiments are summarized in Table 1).

The mice receiving 0.83 mg FCF-2-CF_3_ were sacrificed 24 h after the start of the experiment. They showed the same symptoms as above, but starting later and to a lesser extent. Of the mice injected with 0.28 mg FCF-2-CF_3_, three recovered and lived without apparent symptoms until the end of the experiment (8 days); one mouse was sacrificed 72 h after the start of the experiment due to the appearance of the above-mentioned symptoms. A similar in vivo experiment was also carried out with FCF-2-I; based on the results obtained with FCF-2-CF_3_, the doses were adapted (1.5, 1 and 0.5 mg/mouse), and the number of mice per group was increased to five. As with FCF-2-CF_3_, doses of 1.5 and 1.0 mg elicited rapid systemic toxicity in three out of five and two out of five mice, respectively, and the mice were euthanized before the selected endpoint (day 8; for details, see Table 1). With 0.5 mg, one mouse was euthanized at day 7, one showed mild symptoms from 24 to 96 h, and three mice survived without apparent symptoms.

In summary, FCF-2-CF_3_ and FCF-2-I showed strong and rapid systemic toxicity at doses lower than those tolerated with the parental compound FCF (1.5 mg). That is, the higher potency of FCF-2-CF_3_ and FCF-2-I in vitro is paralleled by an increase in toxicity in vivo, essentially precluding in vivo application.

### 2.4. Treatment of Partially Cisplatin-Resistant MM Cells with FCF or FCF Analogs to Evaluate the Potential of FCF (and Its Analogs) for ‘Combination Therapy’

**Figure 3:**
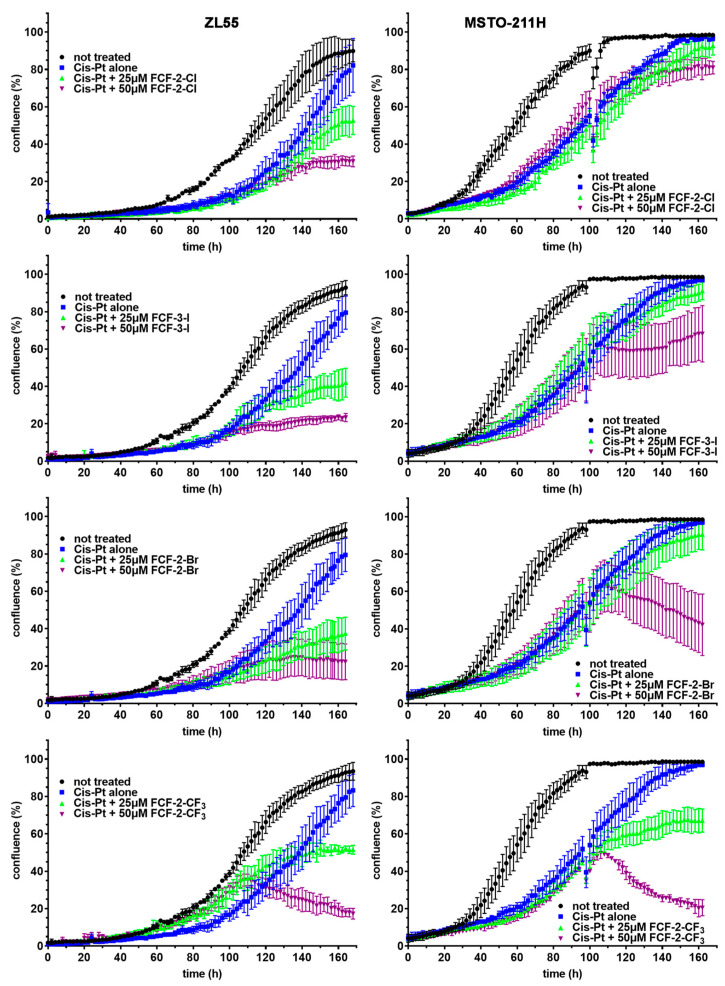
Real-time growth curves of human ZL55 and MSTO-211H cells exposed to cisplatin (Cis-Pt) alone for 96 h, followed by supplemental treatment with FCF-2-Cl, FCF-3-I, FCF-2-Br and FCF-2-CF_3_ for an additional 72 h at concentrations of 25 and 50 µM. The representative growth curves of non-treated MM cells are shown in black. A plateau at near 100% confluence is reached at ~100 h in MSTO-211H cells, and at >140 h in ZL55. Treatment with cisplatin (1.25 µM) strongly reduces MTT signals for the first ~60 h. From then on, an increasing number of cisplatin-resistant cells appear. At 96 h, FCF and its three analogs (FCF-3-I, FCF-2-Br and FCF-2-CF_3_) were added at 25 µM (green curves) and 50 µM (magenta curves), or they were left to grow in the presence of cisplatin only (blue curves). The latter cells reached near-100% confluence at 168 h, while the addition of FCF (and its analogs) decreased the confluence to various extents. The values in the curves represent the mean ± SD from three wells obtained within one experiment. The scattered values at 96 h are the results of the brief removal of the plates from the Incucyte system to add the FCF and its analogs.

The results from MTT assays report on the global effects of a certain treatment on cell proliferation/viability at a defined (often single) time point. However, they don’t allow us to follow the temporal trajectory of the cell numbers, which are the combined effects of cell growth, cell division and cell death. More information is obtained by the acquisition of a series of time-lapse series images (Videos) and derived real-time growth curves. Thus, the effect of the parental FCF (FCF-2-Cl) and the analogs FCF-3-I, FCF-2-Br and FCF-2-CF_3_ was investigated in combination with cisplatin treatment. Cisplatin-pemetrexed chemotherapy—the approved first-line treatment for MM patients—results in the appearance of chemoresistant MM cells, similarly to the effect observed in cisplatin-and pemetrexed-treated MM cells in vitro [9]. Thus, we investigated whether the subpopulations of cisplatin-resistant MM cells were still susceptible to treatment with FCF and its analogs. For this, ZL55, MSTO-211H, and—as a control—Met-5A cells were first treated with cisplatin (1.25 µM). The cell proliferation was monitored in the Incucyte system by recording real-time growth curves. During the first 96 h, the cell proliferation of cisplatin-treated ZL55 and MSTO-211H cells was nearly completely blocked; starting from approximately day 4 onwards, the appearance of slowly growing cisplatin-resistant cell populations was observed (Figure 3, Videos S1–S12,). Thus, from this time point onwards, the MM cells were treated for a further 3 days by the addition of FCF (and its analogs FCF-3-I, FCF-2-Br and FCF-2-CF_3_) at concentrations of 25 µM and 50 µM to the cisplatin-containing cell culture medium. The two concentrations were chosen because they are close to the range of the IC_50_ values for the four compounds (14–48 µM; Supplementary Figure S1) in ZL55 and MSTO-211H cells. Initial experiments were also carried out with SPC212 cells. However, treatment with cisplatin resulted in an extreme flattening of these cells, precluding their accurate detection by the Incucyte system. This resulted in erroneous curves (examples are shown in Supplementary Figure S4); as such, SPC212 cells were not further investigated.

In cells treated with cisplatin only, the cell proliferation of resistant cells increased, resulting in a confluency of approximately 80–100% at day 7. The addition of FCF or the FCF analogs FCF-3-I and FCF-2-Br clearly slowed down cell proliferation in a concentration-dependent manner; at 50 µM, proliferation was nearly blocked. In cells treated with 50 µM FCF-2-Br, and even more so with 50 µM FCF-2-CF_3_, proliferation was not only halted but the cell confluence even decreased considerably, which is indicative of noticeable cell death (Figure 3 and Videos S6 and S12). The treatment of mesothelial Met-5A cells with cisplatin had only a weak effect on cell proliferation (confluence), and the treated cells reached values of about 80–90% at 96 h. Furthermore, in Met-5A cells, FCF-3-I and FCF-2-Br slowed down proliferation, while FCF-2-CF_3_ had an additional cytotoxic effect (data not shown). The strong cytotoxicity exerted by FCF-2-CF_3_ in proliferating non-tumor mesothelial cells in vitro might also be causally linked to the strong systemic toxicity of FCF-2-CF_3_ observed in mice in vivo.

**Figure 4:**
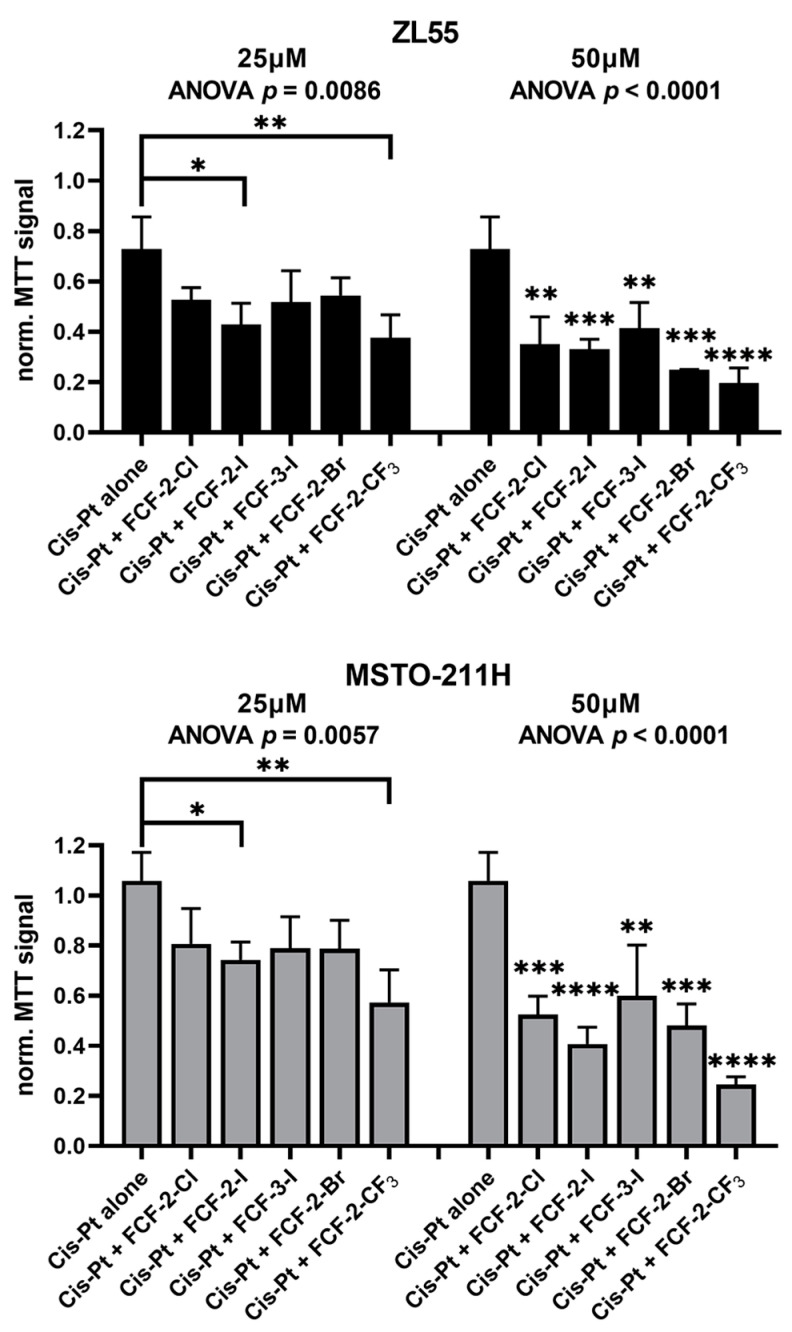
MTT assay reporting the decrease in proliferation/viability of either (I) naïve MM cells exposed to cisplatin alone, or (II) partially cisplatin-resistant MM cells additionally treated with FCF and its analogs for a total of 168 h. ZL55 (**upper**) and MSTO-211H (**lower**) MM cells were exposed to cisplatin for 96 h, followed by the addition of FCF and its analogs (FCF-2-I, FCF-3-I, FCF-2-Br and FCF-2-CF_3_) at 25 µM (**left**) and 50 µM (**right**) for an additional 72 h. Note the concentration-dependent decrease in MTT signal intensity caused by FCF and its analogs in comparison to MM cells treated with cisplatin only at the end of the total exposure time of 168 h. The results are the averages from three to four independent experiments. Each condition was tested in three wells per experiment. The results are shown as the mean ± SD, and the values for untreated cells were set at 1.0. The ANOVA values are shown for the different treatments and cell lines. Significant differences compared to the treatment with cisplatin alone are marked by asterisks, *, **, *** and **** represent *p* < 0.05, *p* < 0.01, *p* < 0.001 and *p* < 0.0001 respectively.

Quantitative analyses of MTT signals obtained at day 7 revealed similar proliferation/viability decreases in ZL55 and MSTO-211H cells (Figure 4). The significant additional effects exerted by the FCF analogs compared to cisplatin alone were more obvious at 50 µM than at 25 µM (the ANOVA and post-hoc analyses are shown in Figure 4). With a concentration of 25 µM, the effects of FCF-2-Cl, FCF-2-I, FCF-3-I and FCF-2-Br were nearly identical in both MM cell lines, i.e., an additional reduction of the MTT signals in the order of ≈20–30% in comparison to cisplatin alone. A slightly stronger (and significant) effect was observed with FCF-2-I and FCF-2-CF_3_. The impairment of proliferation/viability was further enhanced at 50 µM in both MM cell lines. Under these conditions, the FCF-3-I analog was slightly less potent than the 2-analogs (FCF-2-Cl, FCF-2-I and FCF-2-Br), in line with the findings that 3-analogs were generally less potent in epithelioid MM cells (Figure 1). Again, FCF-2-CF_3_ exerted the strongest effect, also at the higher concentration.

In conclusion, FCF and FCF analogs clearly inhibit the proliferation of cisplatin-resistant ZL55 and MSTO-211H cells, and moreover, at 50 µM they decrease the viability of cells treated with this in vitro ‘combination therapy’.

## 3. Discussion

The current lack of efficient treatments for MM patients prompted us to investigate in more detail novel FCF analogs with respect to their suitability for potential therapeutic applications. FCF belongs to the family of diarylureas, a large group containing pharmacologically active compounds, some of which function as serine-threonine kinase or tyrosine kinase inhibitors, and some are used as anti-cancer drugs, e.g., sorafenib and regorafenib; for details, see [29]. FCF was first discovered as a plant fertilizer, and is currently used in agriculture to increase fruit size as the result of its potent cytokinin activity. Unexpectedly, FCF was then discovered to inhibit septin function, first in yeast [16] and then in mammalian cells [17]. This blocking of septin function is assumed to cause the inhibition of cancer cell proliferation, anchorage-independent growth, migration, and the invasion of cancer cell lines derived from various tissues and organs [19,22].

Our previous experiments clearly showed the relevance of the chloride group at the 2-position of the pyridine ring [19]. The importance of the 2-chloropyridine part of FCF was also confirmed by others; a replacement by 2-chloropyrimidine—while retaining the CF_3_S-phenyl moiety in the FCF analog UR214-6—largely abolished the growth-inhibiting effect in ovarian and endometrial cancer cells [22]. However, their main focus was centered on substituents on the phenyl—not the pyridyl—moiety of FCF. The cell growth-inhibiting effect was completely abolished by substitutions including the benzyloxy, pyrimidinyl or pyrrolyl groups (see structures UR214-2, UR214-4 and UR214- 3, respectively, in Table 1 of [22]). The presence of a CF_3_S (UR214-1) or a CF_3_ group together with an additional fluoride group on the phenyl ring (UR214-7) resulted in stronger inhibition in the four investigated cell lines (Figure 2 in [22]). Interestingly, the addition of a CF_3_ group in the 2-position of the pyridine ring also resulted in the strongest growth inhibition of MM cells observed in this study.

It is important to note that in all three structure–activity relationship studies on FCF (and its analogs) in tumor cells, the readout on tumor cell proliferation/viability was a rather indirect one. In classical biochemical assays, one measures the interaction of FCF (and its analogs) with either purified septins or septin heteromers of defined compositions. In such a way, binding constants or—as shown before—changes in thermal stability upon FCF binding to purified His-tagged septins 2, 3 and 7 could be obtained by differential scanning fluorimetry [28]. In the cellular assays, the readout ‘inhibition of cell proliferation/viability’ includes different cellular mechanisms: the kinetics of the uptake and degradation of FCF (and its analogs), cellular half-life, binding to septins and possibly off-targets reported for FCF before [21], and the effects of FCF (and its analogs) metabolites on tumor cell function(s). The fact that the CF_3_ moiety placed at completely different sites (the pyridine ring (this study) vs. the phenyl ring of FCF [22]) had a similar growth-inhibiting/cytotoxic effect might hint towards additional cytotoxic—likely off-target—effects by the CF_3_ group. It is more likely that FCF metabolites containing the CF_3_ moiety might exert their cytotoxic effects, which are not mediated by FCF analog-septin interactions; this hypothesis requires further detailed analyses. In line with this assumption is the fact that unexpected and strong systemic toxicity was observed in mice in vivo after FCF-2-CF_3_ administration, an effect which was not seen after the FCF treatment of mice [19]. This is also in view of the facts that the differences in IC_50_ values in MM cell lines in vitro differed only by a factor of two to three between FCF and FCF-2-CF_3_ (Supplementary Figure S1), and that strong FCF-2-CF_3_-mediated toxicity was observed in vivo already with doses approximately threefold lower than with FCF (0.83 mg vs. 2.5 mg).

While the binding of cations (Ca^2+^, Mg^2+^, Zn^2+^) to specific protein motifs has been comprehensively investigated [30], details on the halide-binding sites of proteins are rather scarce [31,32]. Generally, the halides (F, Cl, Br, I) strongly bind to the guanidinium moiety of arginine side chains, but each halide also displays certain preferences for other amino acids [33]. More important, details on interactions between halogenated organic ligands and macromolecules including proteins leading to the increased stability of protein–ligand complexes are only slowly emerging, and thus remain an understudied topic [34]. Organic halogens show a preference for hydrophobic amino acids (leucine, glycine, phenylalanine), and for serine and threonine. However, preferences exist for each halide for particular amino acids: fluorine has the highest propensity for interaction with glycine, chlorine with leucine, bromine with arginine, and iodine with lysine. If only amino acid side-chain interactions are considered, fluorine and chlorine substitutions have the highest propensities for interactions with leucine, bromine with phenylalanine, and iodine with serine (for more details, see [27]). While these results have yielded information on the preference of certain organic halides for particular amino acids, nothing was known about how the choice of halides in a given small molecule (FCF) affects the cytotoxic effects (the inhibition of proliferation/viability) of MM cells, i.e., on the FCF structure–activity relationship.

In a systematic approach, we investigated the effect of four different halogen atoms in the 2- or 3-position of the pyridine ring of FCF. An in silico study on the binding of FCF to septins indicated that septins are stabilized in a conformation that mimics a nucleotide-bound state, preventing further GTP binding and hydrolysis. Importantly, the hydroxyl group of Thr-186 (protomer A of septin 2) was predicted to form a halogen bond with the chlorine atom of FCF’s pyridine ring (Figure 4A in [30]). Modelling revealed FCF’s interaction with the highly conserved residues present in septins 2, 3, and 7 in a rather similar, but not identical way. Thus, it is conceivable that the various halide substitutions in FCF analogs differently affect the strength of binding to the various septin family members, and thus the readout ‘proliferation/viability’ of MM cells.

In the endometrial and ovarian cancer cell lines ECC-1 and HCH-1, respectively, FCF decreases the expression of HER2 (human epidermal growth factor receptor 2; human gene symbol: *ERBB2*), a protein which is often overexpressed in certain tumor types. Moreover, the survival rates of patients with endometrial cancers are lower if they express higher levels of septins 2, 3 and 7. Furthermore, septins 2 and 9 (not 6 and 7) are elevated in micro-dissected stroma samples obtained from high-grade serous ovarian cancer patients compared to samples from normal ovarian stroma [22]. Thus, one can conclude that altered (often higher) levels of certain septins are associated with specific tumor types. This may hold true not only for tumors from a particular tissue but also possibly for tumor subtypes from the same tissue. In the case of MM, epithelioid and sarcomatoid tumor cells are characterized by clearly distinguishable morphological features, in situ and in vitro. Morphological changes are often linked to alterations in cytoskeletal components and proteins involved in EMT. As an example, ARHGAP4, a member of the Rho family of GTPases, is involved in the regulation of the cell morphology of breast cancer-derived cells. While the down-regulation of ARHGAP4 favors EMT, its overexpression leads to the inverse process, MET [35]. Interestingly, septin 9 negatively regulates ARHGAP4 expression favoring EMT, and the direct overexpression of septin 9 promotes the expression of mesenchymal markers. To our knowledge, no information is currently available regarding whether the distinct MM cell morphologies could be linked to the specific expression profiles of the various septin members. It is noteworthy that the FCF analogs with the 2-position substitutions showed a halogen size-specific effect in (essentially epithelioid) ZL55 and MSTO-211H cells. On the other hand, a halogen size effect was observed in the sarcomatoid SPC212 cells with the FCF analogs with the 3-position substitutions. We reason that these differences might be possibly linked to different septin expression profiles between sarcomatoid and epithelioid MM. The difference in efficiency of the 2- vs. the 3-position halides of FCF analogs might reflect differences in septin expression/composition, which is a hypothesis warranting further detailed investigations; in line with Pous et al. [15], this underscores the importance of septin polymer composition in cancer-related functions.

Because we did not manage to find a single high-potency (low-IC_50_) FCF analog with low systemic toxicity, we investigated an alternative route to inhibit MM cell growth in vitro. One of the likely causes for the relapse of tumor cell growth in MM patients in situ after cisplatin-pemetrexed or γ-ray irradiation treatment is the slow appearance of a treatment-resistant cell population, most probably with characteristics of MSC. This has also been experimentally demonstrated in mice in vivo, where a tenfold lower load of irradiated (likely MSC-enriched) mouse MM cells (RN5, AB12) resulted in a similar tumor incidence and tumor size when compared to injection with untreated cells [11]. This indicates the increased tumor growth/progression potential of MSC. Several upregulated genes were identified among the MM cells that were treated either with cisplatin or subjected to radiation treatment [11], or that were selected by their stem cell-like properties [10]. These MSCs (RN5-SO^high^) were previously shown to be as sensitive to the growth-inhibiting effect of calretinin downregulation as the parental RN5 cells [10], with calretinin being a negative regulator of septin 7 expression and vice versa. Moreover, calretinin and septin 7 directly interact, and are thus co-localized at precise time points during cytokinesis in calretinin-overexpressing ZL55 and MSTO-211H cells [28]. That is, putative calretinin-positive (CR-positive) MSC arising from cisplatin treatment were hypothesized to remain susceptible to the effects of FCF (and possibly its analogs). This was confirmed in the CR-positive MM cell lines ZL55 and MSTO-211H, which were initially treated with cisplatin (1.25 µM) for 4 days, subsequently leading to the appearance of cisplatin-resistant cells. These cells, which were previously shown to be highly enriched in CSC (i.e., EGFP-positive cells; see Figure 2D in [10]), were still susceptible to FCF and the analogs FCF-2-Br, FCF-2-I, FCF-3-I and FCF-2-CF_3_ in vitro. Mechanistically, and with a focus on a putative therapeutic application, this is of importance. While many effects of cisplatin on cancer cells are well-studied—including DNA damage leading to apoptosis, oxidative stress, the modulation of Ca^2+^ signaling, and the activation of various cellular (often kinase) pathways (for a review, see [36,37]—it is currently unknown whether, at some stage, cytoskeletal components including septins are involved in the mechanisms leading either to cell death or development of resistance. That is, if the pathways of the induction of cell death or resistance by cisplatin and FCF would converge at some point, FCF would be expected to be ineffective in cisplatin-resistant cells. Our results indicate that cisplatin and FCF target essentially independent pathways, such that FCF still impairs the proliferation/viability of cisplatin-resistant MM cells.

This susceptibility was evidenced by the inhibition of cell proliferation, as well as increased cell death in the population of cisplatin-resistant MM cells, with the latter being particularly notable for FCF-2-CF_3_. In summary, FCF and its analogs may represent a new family of compounds to target cisplatin-resistant MM cells, a subpopulation that quickly emerges after the temporary reduction of MM tumor mass following first-line MM treatment. Additional studies are required in order to further develop novel FCF analogs with increased potency (lower IC_50_ values in vitro), but importantly with tolerable pharmaco-toxic profiles.

## 4. Materials and Methods

### 4.1. Cell Culture

The MM cell lines ZL55, SPC212 (obtained from the University Hospital, Zurich, Switzerland) and MSTO-211H (American Type Culture Collection; ATCC, Manassas, VA, USA) were grown in RPMI1640 medium (Gibco, Basel, Switzerland) supplemented with 10% fetal bovine serum (FBS, Gibco), 100 U/mL penicillin, and 100 µg/mL streptomycin (1% PS, Gibco). The SV40-immortalized human mesothelial cell line Met-5A (ATCC) was maintained in Dulbecco’s modified Eagle’s medium/F-12 1:1 plus GlutaMax (Gibco) supplemented with 10% FBS (fetal bovine serum), 100 U/mL penicillin, and 100 µg/mL streptomycin (1% PS, Gibco).

### 4.2. Treatment of the Cells with FCF and Its Analogs, and in Combination with Cisplatin

The cells were seeded in 96-well plates (1000 cells per well for MM cells and 2000 cells for Met-5A) and grown for 24 h. FCF (CAS 68157-60-8, LabForce AG, Muttenz, Switzerland) and the synthetized analogs were added in a concentration range from 0 to 50 µM, and MTT assays were performed 96 h post-treatment in order to determine the number of viable and/or proliferating cells [38]. Real-time cell proliferation curves (based on cell confluence) and images were acquired with the Incucyte live-cell imaging system (Essen Bioscience, Ann Arbor, MI, USA), as described before [39]. In the ‘combination therapy’ experiments, the cells were initially treated with cisplatin (1.25 µM) for 96 h. Subsequently, FCF (and its analogs) were added for an additional 72 h at concentrations of 25 and 50 µM.

### 4.3. In Vivo Toxicity Test with Mice Treated with FCF-2-I and FCF-2-CF_3_

The experiments were performed according to institutional guidelines of the present Swiss law and the European Communities Council Directive of 24 November 1986 (86/609/EEC). The authorization number for the housing of mice is H-04.2012-Fr, and those for the experiments are 2017_32_FR and 2021_07_FR. All of the experiments were approved by the Cantonal Veterinary Office (Canton of Fribourg, Switzerland). BALB/c mice (males, 3–6 months-old) were divided into 4 experimental groups, i.e., 3 treatment groups and a control group. The two tested substances—FCF-2-CF_3_ and FCF-2-I—were dissolved in DMSO and then diluted in sterile propylene glycol, as described previously for FCF [19]. Three doses per substance (all in a volume of 200 µL propylene glycol (PG) containing 0.5% DMSO)—and, in the control group, 200 µL solvent only—were injected i.p. In the experiment with FCF-2-CF_3_, the doses were 2.5, 0.83 and 0.28 mg/mouse, the mouse age was 3 months, and the group size was 4 mice. For the later experiments with FCF-2-I, the doses were 1.5, 1.0 and 0.5 mg/mouse, the mice were 6 months old, and the group size was increased to five. In order to confirm our previous in vivo results with FCF, and to directly compare them with the novel FCF analogs, 10 mice were treated with 1.5 mg FCF. The health status of the mice was monitored by visual inspection at short intervals; every hour during the first 8 h, and then at progressively longer intervals (2–4 times per 24 h) for up to 8 days.

### 4.4. Synthesis and Characterization of the FCF Analogs

Details on the synthesis and characterization of all ten novel diarylureas used in this study are described in the Supplementary Information section.

### 4.5. Statistical Analysis

The statistical analyses (ANOVA followed by Tukey’s multiple comparisons test) were performed using GraphPad Prism 9.3.1 software (GraphPad Software, San Diego, CA, USA).

## Figures and Tables

**Table 1:** Summary of the results from the in vivo mouse experiments with FCF-2-CF_3_ and FCF-2-I.

Dose/Substance	Symptoms *	Severity of Symptoms	Animals with Symptoms after 24 h **	Animals Recovering	Timepoint of Euthanasia in Non-Recovering Mice
0.28 mg FCF-2-CF_3_	1/2/3/4/5	strong	4(4)	3(4)	72 h
0.83 mg FCF-2-CF_3_	1/2/3/4/5	very strong	4(4)	0(4)	24 h
2.50 mg FCF-2-CF_3_	1/2/3/4/5	very strong	n/a **	0(4)	6 h
0.5 mg FCF-2-I	1/2/5	mild	2(5)	4(5)	168 h
1.0 mg FCF-2-I	1/2/5	mild	2(5)	3(5)	96 h
1.5 mg FCF-2-I	1/2/5	strong	4(5)	2(5)	72 h

* Description of symptoms: 1, reduced activity/exploring; 2, reduced social interaction; 3, trembling/staggering; 4, hunchback posture; 5, shaggy fur. ** The mice treated with 2.5 mg FCF-2-CF_3_ were euthanized 6 h after treatment.

## Data Availability

The data presented in this study are available in the Supplementary Material of this article.

## Supplementary Materials

The following supporting information can be downloaded at: https://www.mdpi.com/article/10.3390/ijms23073963/s1. Reference [40] is cited in the Supplementary Materials.
