# Supplementary material for: Forchlorfenuron and Novel Analogs Cause Cytotoxic Effects in Untreated and Cisplatin-Resistant Malignant Mesothelioma-Derived Cells
Source: Int J Mol Sci. 2022 Apr 2;23(7):3963. doi: 10.3390/ijms23073963 (PMC8999537; doi:10.3390/ijms23073963)

Supplemental Figures S1 – S4, Tables S1 & S2 & Supporting Information for Henzi *et al.* (2022)

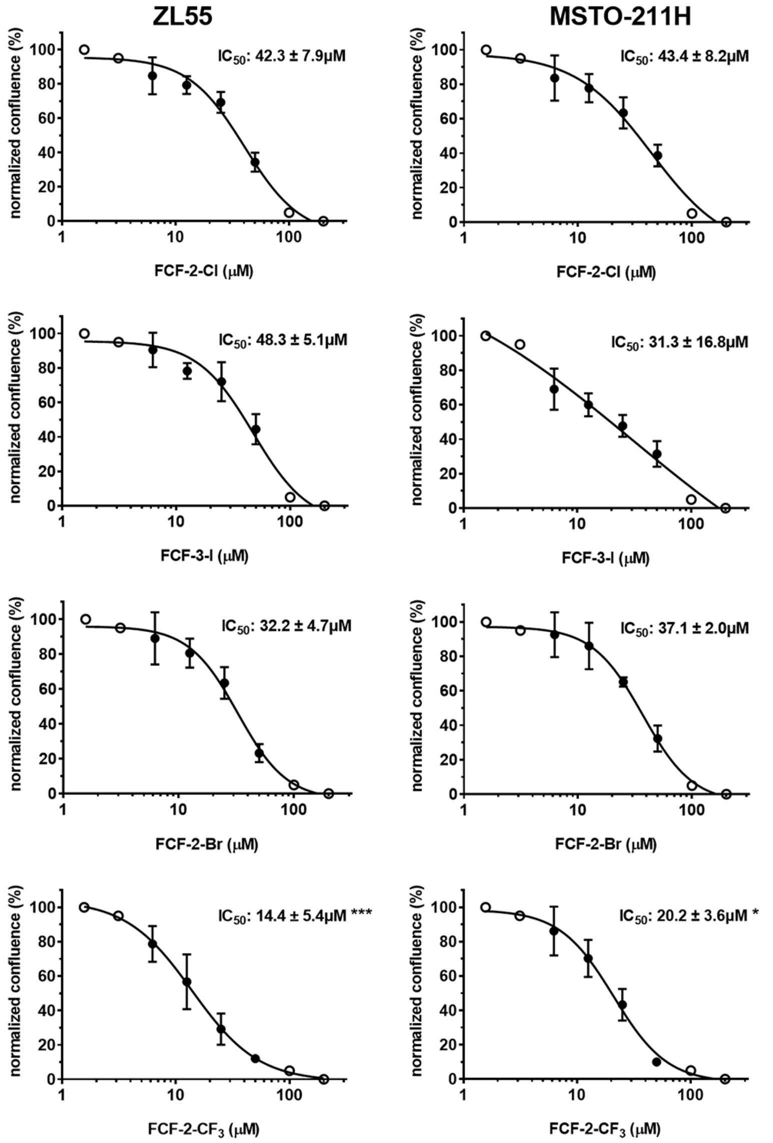

Suppl. Figure S1 Determination of IC_{50} for FCF-2-Cl (parental FCF), FCF-3-I, FCF-2-Br and FCF-CF_3 in ZL 55 (left) and MSTO-211H (right) cells. The reported IC_{50} values are the mean ± SD from 3 – 4 independent experiments (each condition measured in 3 wells) with concentrations of 6.25, 12.5, 25 and 50 µM. Based on previous results from Blum et al. (Blum et al., 2018), values for lower concentrations (1.5, 3.125 µM) were set as 100 and 95% respectively and for higher concentrations (100, 200 µM) as 5 and 0%, respectively (open symbols). Changing of these preset values (± 5%) have only a minor effect on the estimated IC_{50} values (Suppl. Figure S2). ANOVA revealed significant differences among the four tested compounds in ZL55 (p<0.0001) and MSTO-211H cells (p<0.05). Pairwise comparisons by post-hoc analysis (FCF *vs.* the three FCF analogs) showed that significantly lower IC_{50} values were only observed with FCF-2-CF_3 (*** p<0.001 for ZL55 cells; * p<0.05 for MSTO-211H cells). For clarity, the average curves calculated from all data points derived from 3 – 4 independent experiments (mean ± SD; full symbols) are depicted).

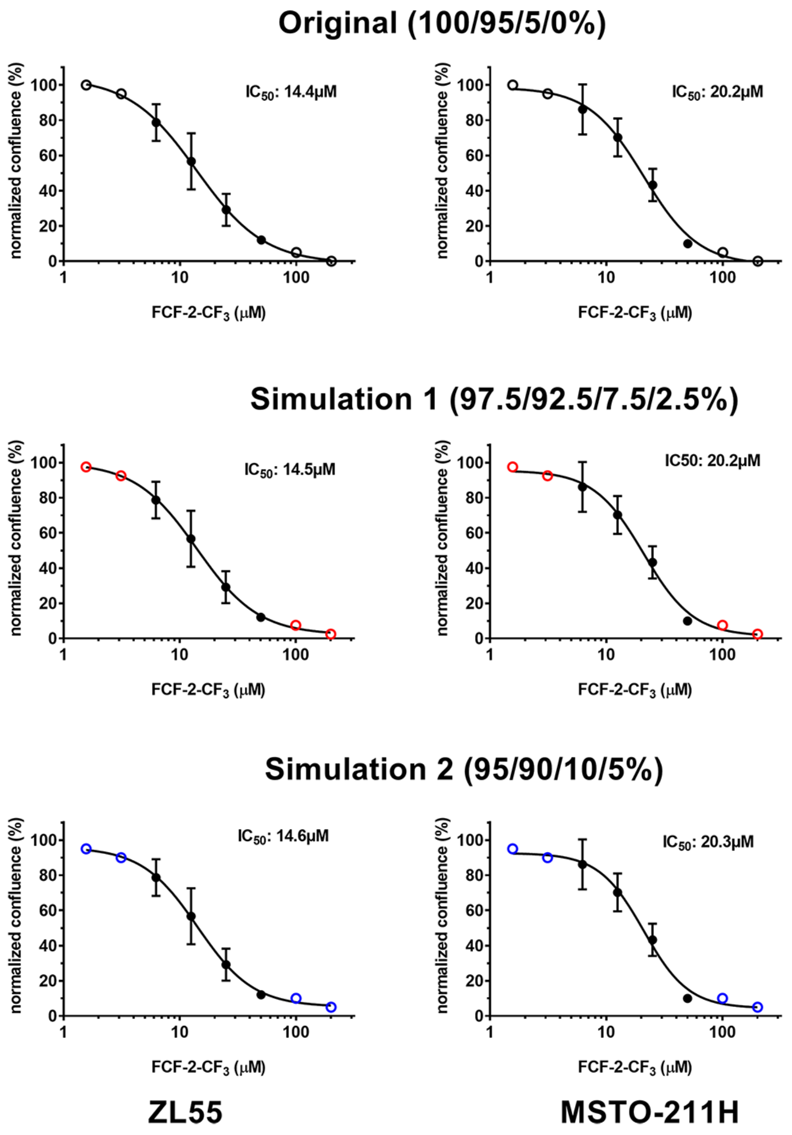

Suppl. Figure S2 Effect of changing upper and lower values used in suppl. Figure S1 for estimation of IC_{50} for FCF-CF_3 in ZL 55 (left) and MSTO-211H (right) cells. In the upper part, values shown in suppl. Figure S1 are depicted. That is, upper values were set at 100 and 95% for 1.5 and 3.125 µM, the lower ones at 5 and 0% for 100 and 200 µM, respectively (black open circles). In the middle panels, these values were set at 97.5 and 92.5% (upper) and 7.5 and 2.5% (lower; red open circles). In the lower panels values were set at 95, 90, 10 and 5% (blue open circles). Differences in IC_{50} values obtained in the 3 calculations (original and 2 simulations) were less than 2% in both cell lines and considerably smaller than the SD values from the 4 independent experiments carried out in both cell lines.

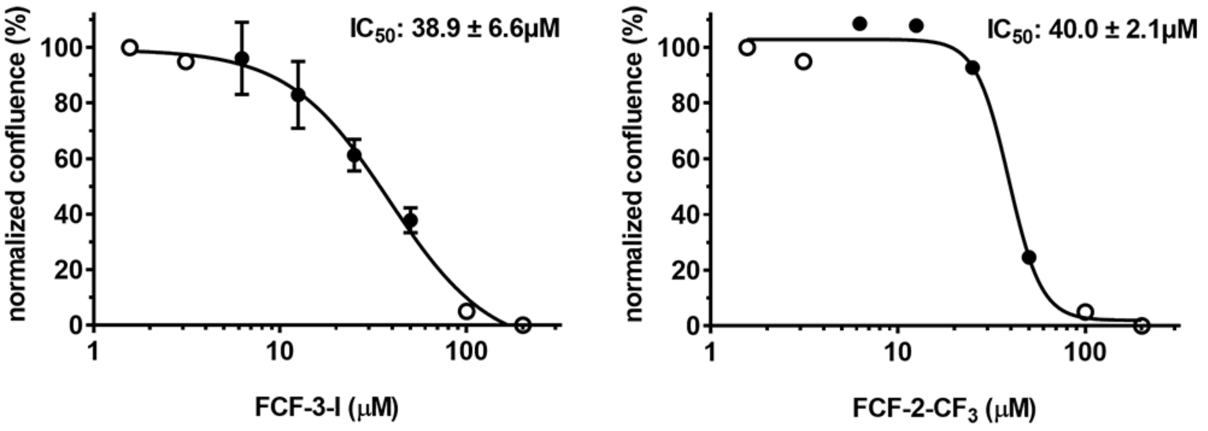

Suppl. Figure S3 Determination of IC_{50} for FCF-3-I and FCF-2-CF_3 in SPC212 cells. All details are identical as mentioned in Suppl. Figure 1 legend.

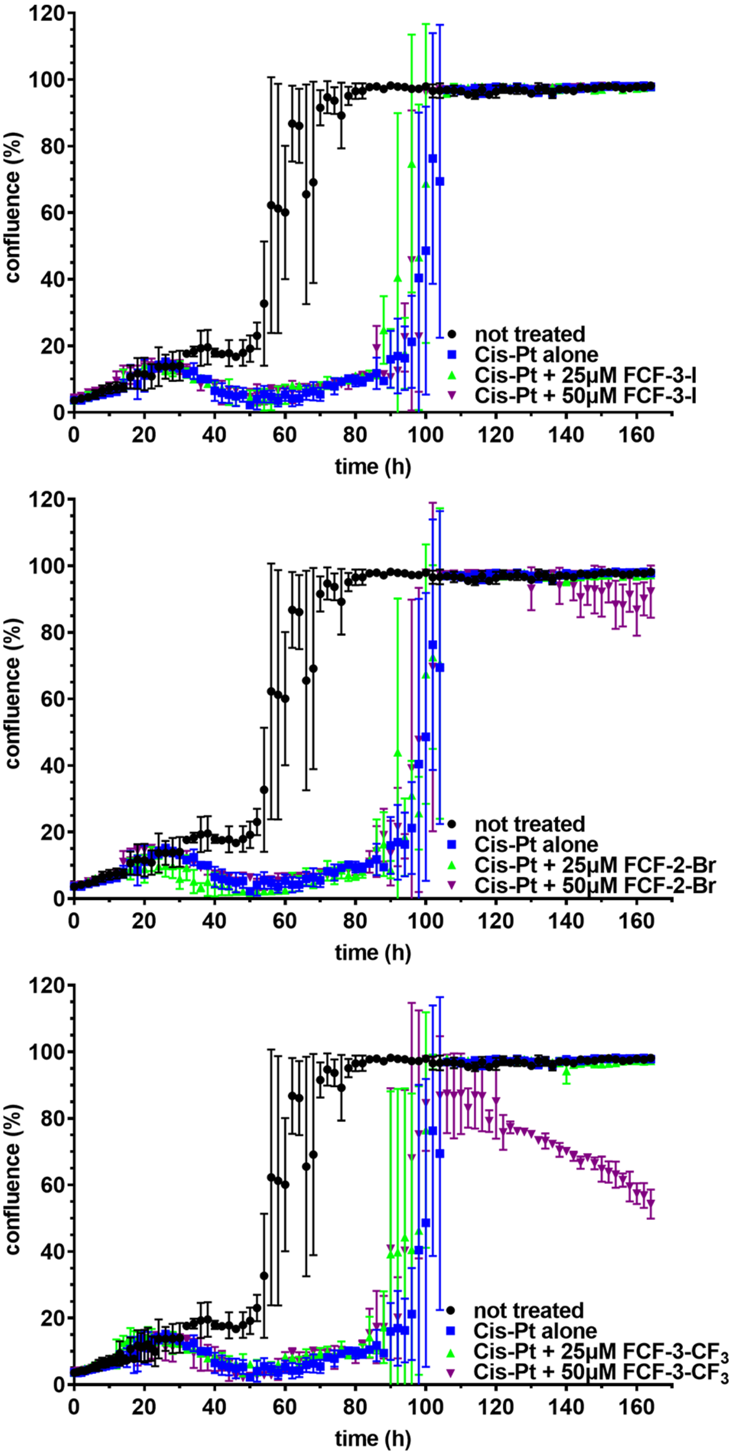

Suppl. Figure S4 Real-time growth curves of SPC212 cells exposed to Cis-Pt alone for 96 h, followed by additional treatment with FCF-3-I, FCF-2-Br, and FCF-2-CF$_3$ for an additional 72 h at concentrations of 25 and 50 µM. All details are identical as shown in Figure 3. Note the huge variation (standard deviation) in signal intensity during the phase of logarithmic cell growth resulting from cell flattening preventing accurate detection by the Incucyte system.

Supplementary Table S1

Compound number	Name Abbreviation	Structure	Molecular weight
**1**	1-(2-fluoropyridin-4-yl)-3-phenylurea **FCF-2-F**	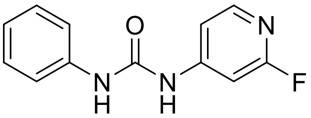	231.23
**2**	1-(2-fluoropyridin-4-yl)-3-phenylurea **FCF-3-F**	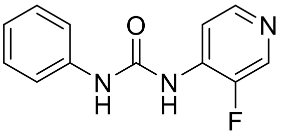	231.23
**3**	1-(2-chloropyridin-4-yl)-3-phenylurea **FCF-2-Cl « FCF »**	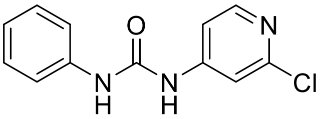	247.68
**4**	1-(3-chloropyridin-4-yl)-3-phenylurea **FCF-3-Cl**	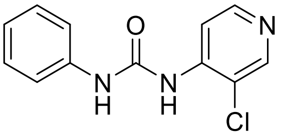	247.68
**5**	1-(2-bromopyridin-4-yl)-3-phenylurea **FCF-2-Br**	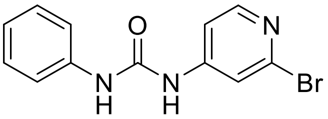	292.14
**6**	1-(3-bromopyridin-4-yl)-3-phenylurea **FCF-3-Br**	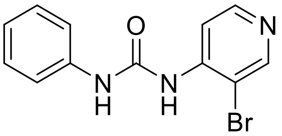	292.14
**7**	1-(2-iodopyridin-4-yl)-3-phenylurea **FCF-2-I**	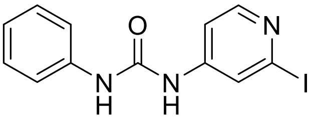	339.13
**8**	1-(3-iodopyridin-4-yl)-3-phenylurea **FCF-3-I**	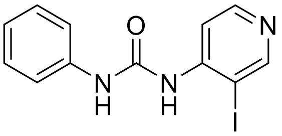	339.13
**9**	1-phenyl-3-(2-(trifluoromethyl)pyridin-4-yl)urea **FCF-2-CF$_3$**	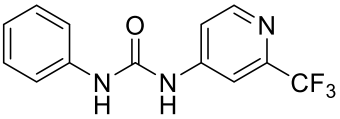	281.24
**10**	1-(2-methylpyridin-4-yl)-3-phenylurea **FCF-2-CH$_3$**	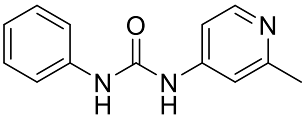	227.27
**11**	1-(2-methoxypyridin-4-yl)-3-phenylurea **FCF-2-OMe**	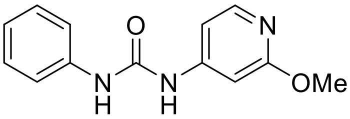	243.27

Supplementary Table S2

Pairwise comparisons of effects caused by treatment of MM cells with FCF analogs (50 µM) *vs.* untreated control cells. Initial ANOVA had revealed significant differences between groups. Significant differences are marked in black, insignificant differences are marked in red.

ZL55 cells

**Tukey's multiple comparisons test**	**Mean Diff.**	**95,00% CI of difference**	**Summary**	**Adjusted *P* Value**
FCF-2-F vs. Control	-0,4926	-0,8330 to -0,1522	***	0,0006
FCF-3-F vs. Control	-0,479	-0,7999 to -0,1580	***	0,0004
FCF-2-Cl vs. Control	-0,5975	-0,9379 to -0,2571	****	<0,0001
FCF-3-Cl vs. Control	-0,4911	-0,8315 to -0,1506	***	0,0007
FCF-2-Br vs. Control	-0,708	-1,048 to -0,3676	****	<0,0001
FCF-3-Br vs. Control	-0,5148	-0,8553 to -0,1744	***	0,0003
FCF-2-I vs. Control	-0,8277	-1,149 to -0,5068	****	<0,0001
FCF-3-I vs. Control	-0,6603	-1,001 to -0,3199	****	<0,0001
FCF-2-CF_3 vs. Control	-0,8213	-1,162 to -0,4809	****	<0,0001
FCF-2-CH_3 vs. Control	-0,3115	-0,6519 to 0,02896	ns	0,1002
FCF-2-OCH_3 vs. Control	0,004301	-0,3361 to 0,3447	ns	>0,9999

MSTO-211H cells

**Tukey's multiple comparisons test**	**Mean Diff.**	**95,00% CI of difference**	**Summary**	**Adjusted *P* Value**
FCF-2-F vs. Control	-0,5959	-0,8241 to -0,3676	****	<0,0001
FCF-3-F vs. Control	-0,6423	-0,8864 to -0,3983	****	<0,0001
FCF-2-Cl vs. Control	-0,6153	-0,8436 to -0,3870	****	<0,0001
FCF-3-Cl vs. Control	-0,5961	-0,8244 to -0,3678	****	<0,0001
FCF-2-Br vs. Control	-0,7264	-0,9547 to -0,4981	****	<0,0001
FCF-3-Br vs. Control	-0,6146	-0,8429 to -0,3863	****	<0,0001
FCF-2-I vs. Control	-0,8144	-1,058 to -0,5704	****	<0,0001
FCF-3-I vs. Control	-0,7324	-0,9607 to -0,5041	****	<0,0001
FCF-2-CF_3 vs. Control	-0,9037	-1,132 to -0,6754	****	<0,0001
FCF-2-CH_3 vs. Control	-0,4703	-0,6986 to -0,2420	****	<0,0001
FCF-2-OCH_3 vs. Control	-0,1591	-0,3874 to 0,06917	ns	0,4049

SPC212 cells

**Tukey's multiple comparisons test**	**Mean Diff.**	**95,00% CI of difference**	**Summary**	**Adjusted *P* Value**
FCF-2-F vs. Control	-0,1773	-0,4797 to 0,1250	ns	0,6668
FCF-3-F vs. Control	-0,3464	-0,6314 to -0,06135	**	0,0068
FCF-2-Cl vs. Control	-0,1855	-0,4878 to 0,1169	ns	0,6055
FCF-3-Cl vs. Control	-0,4404	-0,7427 to -0,1381	***	0,0006
FCF-2-Br vs. Control	-0,2958	-0,5981 to 0,006521	ns	0,06
FCF-3-Br vs. Control	-0,5566	-0,8589 to -0,2542	****	<0,0001
FCF-2-I vs. Control	-0,5926	-0,8777 to -0,3076	****	<0,0001
FCF-3-I vs. Control	-0,6764	-0,9787 to -0,3740	****	<0,0001
FCF-2-CF_3 vs. Control	-0,7217	-1,024 to -0,4194	****	<0,0001
FCF-2-CH_3 vs. Control	-0,3324	-0,6347 to -0,03004	*	0,0207
FCF-2-OCH_3 vs. Control	-0,2936	-0,5959 to 0,008698	ns	0,0637

Legends to Videos S1 – S12

Time-lapse series (168 h) acquired on the Incucyte system of ZL55 (Videos 1 – 6) and MSTO-211H (Videos 7 – 12) MM cells

S1, S7 untreated control cells

S2, S8 cells treated with 1.25 µM Cis-Pt for 168 h

S3, S9 cells treated with Cis-Pt (1.25 µM) for 96 h, then supplemented with FCF-2-Cl (50 µM) for 72 h

S4, S10 cells treated with Cis-Pt (1.25 µM) for 96 h, then supplemented with FCF-3-I (50 µM) for 72 h

S5, S11 cells treated with Cis-Pt (1.25 µM) for 96 h, then supplemented with FCF-2-Br (50 µM) for 72 h

S6, S12 cells treated with Cis-Pt (1.25 µM) for 96 h, then supplemented with FCF-2-CF_3 (50 µM) for 72 h

Supporting Information

General Methods (chemicals, analytical methods)

Chemicals were obtained from commercial suppliers (TCI, Acros Organics, Sigma-Aldrich, Fluorochem, Apollo Scientific). Dry toluene was obtained from drying columns from the Chemistry Department of UNIFR. Thin layer chromatography was performed on Merck TLC Silicagel 60 F_{254} or TLC Silica gel 60 RP-18 $F_{254}S$. UV light (254 nm) and/or a potassium permanganate stain were used to visualize the spots on TLCs. NMR measurements were carried out on a Bruker Avance 300 MHz spectrometer (^{1}H: 300 MHz, ^{13}C: 75 MHz) or a Bruker Avance III HD 400 Mhz spectrometer (^{1}H: 400 MHz, ^{13}C: 101 MHz). Chemical shifts are expressed in parts per million (ppm) using residual solvent protons as internal standards. Coupling constants (J) are reported in Hz. Splitting patterns are designated as s (singlet), d (doublet), dd (double doublet), t (triplet), dt (double triplet), q (quartet), br. s. (broad singlet), m (multiplet). Deuterated solvents were obtained from Cambridge Isotope Laboratories. IR spectra were recorded on a Bruker FT-IR Tensor II using a Golden Gate diamond ATR system. Melting point measurements were performed using a Büchi Melting Point B-540 device. ESI-MS was carried out using a Bruker esquire HCT Ion trap mass spectrometer.

General procedure for the synthesis or diarylureas

Ureas were synthesized by the reaction of phenyl isocyanate with various 4-aminopyridines (see below) in toluene (PhMe) at 80°C. To a solution of the isocyanate (1.2 eq) in dry toluene (C = 0.2 M) was added the 4-aminopyridine (1 eq). The solution was heated at 80°C in a sealed tube for 4 to 6 hours. If precipitation occurred upon cooling to room temperature, the solution was filtered to isolate the crude product. If not, the solvent was removed under reduced pressure. In both cases, the crude mixture was either recrystallized in acetonitrile or purified by column chromatography to obtain the desired diarylurea.

1-(2-fluoropyridin-4-yl)-3-phenylurea

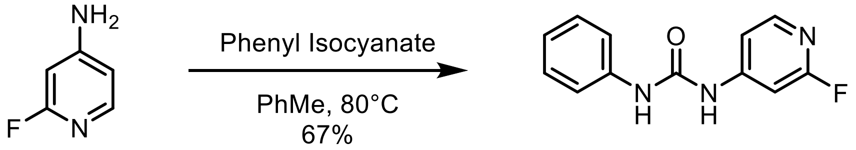

Synthesized according to the general procedure by the reaction of 2-fluoropyridin-4-amine (324 mg, 2.89 mmol) with phenyl isocyanate. It was then purified by column chromatography (30% to 80% EtOAc in heptane, silica gel) to obtain 1-(2-fluoropyridin-4-yl)-3-phenylurea (453 mg, 1.96 mmol, 67%) as a white solid.

^{1}H NMR (400 MHz, Acetone-d_6) δ = 8.74 (br. s., 1 H), 8.32 (br. s., 1 H), 8.00 (d, *J* = 5.6 Hz, 1 H), 7.56 - 7.50 (m, 2 H), 7.36 (d, *J* = 1.7 Hz, 1 H), 7.34 - 7.28 (m, 2 H), 7.26 (td, *J* = 1.7, 5.7 Hz, 1 H), 7.08 - 7.01 (m, 1 H) **^{13}C NMR** (101 MHz, Acetone-d_6) δ = 166.2 (d, *J* = 230.3 Hz), 153.2, 152.8 (d, *J* = 11.7 Hz), 149.1 (d, *J* = 18.3 Hz), 140.5, 130.2, 124.4, 120.5, 112.3 (d, *J* = 2.9 Hz), 98.0 (d, *J* = 44.8 Hz) **FT-IR** (ATR, neat, cm^{-1}) 3334, 3264, 3187, 3104, 3062, 1714, 1665, 1592, 1523, 1498, 1480, 1444, 1413, 1345, 1315, 1297,1270, 1258, 1214, 1155, 1117, 1048, 1029, 985, 867, 854, 831, 797, 775, 754.49, 738.70, 705.97, 690, 638 **ESI-MS** *m/z* calcd for $C_{12}H_{10}FN_3O$ $[M + H]^+$ 232.1, found 232.1 **Melting point**: 175-176°C

1-(3-fluoropyridin-4-yl)-3-phenylurea

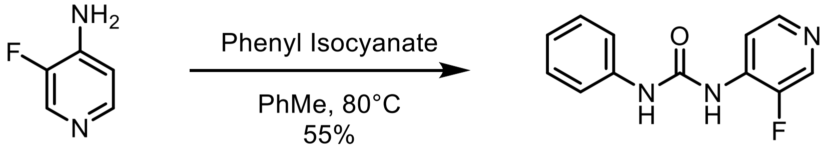

Synthesized according to the general procedure by the reaction of 3-fluoropyridin-4-amine (176 mg, 1.59 mmol) with phenyl isocyanate. It was then purified by column chromatography (35% to 80% EtOAc in heptane, silica gel) to obtain 1-(3-fluoropyridin-4-yl)-3-phenylurea (201 mg, 0.87 mmol, 55%) as a white solid.

^{1}H NMR (400 MHz, Acetone-d_6) δ = 8.70 (br. s., 1 H), 8.48 (br. s., 1 H), 8.43 - 8.31 (m, 2 H), 8.26 (d, *J* = 5.4 Hz, 1 H), 7.62 - 7.45 (m, 2 H), 7.39 - 7.24 (m, 2 H), 7.13 - 6.95 (m, 1 H) **^{13}C NMR** (101 MHz, Acetone-d_6) δ = 152.4, 150.2 (d, *J* = 249.4 Hz), 140.0, 137.5, 137.3, 136.0 (d, *J* = 8.1 Hz), 129.8, 123.9, 119.7, 114.3 **FT-IR** (ATR, neat, cm^{-1}) 3451, 3335, 3274, 3035, 1707, 1608, 1596, 1560, 1529, 1500, 1489, 1445, 1431, 1302, 1249, 1210, 1200, 1179, 1156, 1042, 893, 822, 748, 695, 639 **ESI-MS** *m/z* calcd for $C_{12}H_{10}FN_3O$ $[M + H]^+$ 232.1, found 232.1 **Melting point**: 169 - 170°C

1-(3-chloropyridin-4-yl)-3-phenylurea

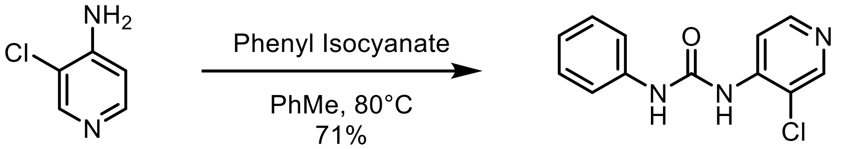

Synthesized according to the general procedure by the reaction of 4-amino-3-chloropyridin (100 mg, 0.778 mmol) with phenyl isocyanate. It was then purified by column chromatography (35% to 80% EtOAc in heptane, silica gel) to obtain 1-(3-chloropyridin-4-yl)-3-phenylurea (137 mg, 0.554 mmol, 71%) as a white solid.

^{1}H NMR (300 MHz, DMSO-d$_6$) δ = 9.68 (s, 1 H), 8.70 (s, 1 H), 8.51 (s, 1 H), 8.34 (d, *J* = 5.7 Hz, 1 H), 8.28 (d, *J* = 5.6 Hz, 1 H), 7.51 - 7.42 (m, 2 H), 7.40 - 7.27 (m, 2 H), 7.10 - 6.97 (m, 1 H) **^{13}C NMR** (75MHz, DMSO-d$_6$) δ = 151.5, 148.9, 148.7, 143.0, 138.8, 129.0, 122.8, 118.5, 118.4, 113.3 **FT-IR (**ATR, neat, cm^{-1})3527, 3357, 3274, 3204, 3129, 3081, 3034, 1724, 1624, 1588, 1557, 1495, 1444, 1411, 1306, 1251, 1196, 1176, 1091, 1036, 1022, 895, 842, 816, 749, 690, 639 **ESI-MS** *m/z* calcd for $C_{12}H_{10}ClN_3O$ [M + H]$^+$ 248.2, found 248.2 **Melting point:** 163 – 164°C

1-(2-bromopyridin-4-yl)-3-phenylurea

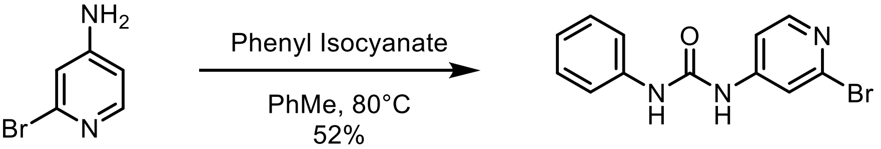

Synthesized according to the general procedure by the reaction of 2-bromopyridin-4-amine (503 mg, 2.90 mmol) with phenyl isocyanate. It was then recrystallized from hot acetonitrile to obtain 1-(2-bromopyridin-4-yl)-3-phenylurea (441 mg, 1.51 mmol, 52%) as white crystals.

^{1}H NMR (400 MHz, DMSO-d$_6$) δ = 9.32 (s, 1 H), 8.97 (s, 1 H), 8.15 (d, *J* = 5.6 Hz, 1 H), 7.81 (d, *J* = 1.8 Hz, 1 H), 7.53 - 7.40 (m, 2 H), 7.40 - 7.24 (m, 3 H), 7.09 - 6.90 (m, 1 H) **^{13}C NMR** (101 MHz, DMSO-d$_6$) δ = 151.8, 150.4, 148.8, 142.0, 138.7, 128.8, 122.7, 118.8, 114.9, 112.2 **FT-IR (**ATR, neat, cm^{-1}) 3396, 3239, 3128, 3029, 2978, 1729, 1579, 1527, 1494, 1468, 1439, 1390, 1302, 1267, 1244, 1191, 1158, 1124, 1068, 1043, 1025, 986, 921, 892, 875, 832, 747, 701, 687, 634, 615 **ESI-MS** *m/z* calcd for $C_{12}H_{10}BrN_3O$ [M + H]$^+$ 294.0, found 293.8 **Melting point:** 177-178 °C

1-(3-bromopyridin-4-yl)-3-phenylurea

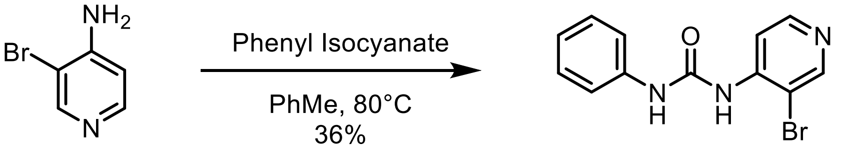

Synthesized according to the general procedure by the reaction of 4-amino-3-bromopyridine (1.01 g, 5.86 mmol) with phenyl isocyanate. It was then purified by column chromatography (50% to 80% EtOAc in heptane, silica gel) to obtain 1-(3-bromopyridin-4-yl)-3-phenylurea (580 mg, 1.99 mmol, 36%) as a white solid.

^{1}H NMR (400 MHz, DMSO-d_6) δ = 9.79 (br. s., 1 H), 8.61 (s, 1 H), 8.48 (br. s., 1 H), 8.35 (d, *J* = 5.6 Hz, 1 H), 8.24 (d, *J* = 5.6 Hz, 1 H), 7.53 - 7.42 (m, 2 H), 7.41 - 7.28 (m, 2 H), 7.11 - 6.98 (m, 1 H) **^{13}C NMR** (101 MHz, DMSO-d_6) δ = 151.6, 151.5, 149.0, 144.1, 138.8, 129.0, 122.7, 118.5, 114.0, 109.4 **FT-IR (**ATR, neat, cm^{-1}) 3534, 3344, 3266, 3203, 3125, 3083, 3032, 2984, 2873, 2811, 2754, 1722, 1707, 1625, 1585, 1557, 1494, 1444, 1410, 1305, 1251, 1196, 1174, 1083, 1018, 895, 841, 813, 750, 724, 690, 638 **ESI-MS** *m/z* calcd for $C_{12}H_{10}BrN_3O$ [M + H]$^+$ 294.0, found 293.8 **Melting point:** 166 - 169° C

2-iodopyridin-4-amine Synthesized using the method described by (Jin and Davies, 2017)

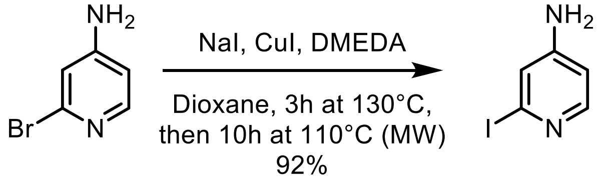

N1,N2-dimethylethane-1,2-diamine (31 µL, 0.1 Eq, 289 µmol), sodium iodide (866 mg, 2 Eq, 5.78 mmol), copper(I) iodide (27 mg, 0.05 Eq, 145 µmol) and 2-bromopyridin-4-amine (500 mg, 1 Eq, 2.89 mmol) was heated under argon in dry 1,4-Dioxane (10 mL) for 3h at 130°C. It was then heated for 10h at 110°C. The solution was diluted with saturated $NaHCO_3$ and extracted with EtOAc (3x15 mL). The combined organic layers were dried over sodium sulfate and concentrated under reduced pressure to obtain 2-iodopyridin-4-amine (587 mg, 2.67 mmol, 92 %) as a yellow oil with no further purification.

Note: We are aware that judging from other 4-aminopyridines, the product was expected to be a solid. It however never crystallized in our hands. A few percent (3-5%) of the starting bromide remains and could not be separated. This however is not a problem for the next step as the amount of contaminating bromide could be even more reduced by recrystallizing the product.

¹H NMR (400 MHz, CD_3CN) δ = 7.86 - 7.70 (m, 1 H), 6.97 (d, *J* = 2.0 Hz, 1 H), 6.59 - 6.44 (m, 1 H), 4.94 (br. s., 2 H) **¹³C NMR** (101 MHz, CD_3CN) δ = 156.5, 151.5, 120.0, 119.9, 110.7 **ESI-MS** *m/z* calcd for $C_5H_6IN_2$ $[M + H]^+$ 220.9 found 221.2 **FT-IR (**ATR, neat, cm^{-1}) 3442, 3302, 3157, 1635, 1578, 1531, 1474, 1408, 1340, 1282, 1245, 1123, 1056, 976, 885, 872, 846, 826, 702

¹H NMR (400 MHz, CD_3CN) δ = 7.86 - 7.70 (m, 1 H), 6.97 (d, *J* = 2.0 Hz, 1 H), 6.59 - 6.44 (m, 1 H), 4.94 (br. s., 2 H) **¹³C NMR** (101 MHz, CD_3CN) δ = 156.5, 151.5, 120.0, 119.9, 110.7 **ESI-MS** *m/z* calcd for $C_5H_6IN_2$ $[M + H]^+$ 220.9 found 221.2 **FT-IR (**ATR, neat, cm^{-1}) 3442, 3302, 3157, 1635, 1578, 1531, 1474, 1408, 1340, 1282, 1245, 1123, 1056, 976, 885, 872, 846, 826, 702

1-(2-iodopyridin-4-yl)-3-phenylurea

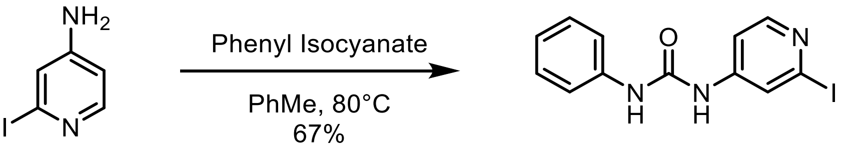

Synthesized according to the general procedure by the reaction of 4-amino-3-iodopyridine (405 mg, 1.86 mmol) with phenyl isocyanate. It was then purified by column chromatography (40% to 80% EtOAc in heptane, silica gel), followed by a recrystallization from hot acetonitrile to obtain 1-(2-iodopyridin-4-yl)-3-phenylurea (425 mg, 1.25 mmol, 67%) as a white solid.

¹H NMR (400 MHz, Acetone-d_6) δ = 8.55 (br. s., 1 H), 8.32 (br. s., 1 H), 8.17 - 8.12 (m, 1 H), 8.10 (d, *J* = 5.6 Hz, 1 H), 7.56 - 7.50 (m, 2 H), 7.42 (dd, *J* = 2.1, 5.6 Hz, 1 H), 7.35 - 7.26 (m, 2 H), 7.08 - 7.01 (m, 1 H) **¹³C NMR** (101MHz, Acetone-d_6) δ = 151.6, 150.6, 147.8, 139.0, 128.8, 122.9, 122.4, 119.0, 118.2, 112.4 **FT-IR (**ATR, neat, cm^{-1}) 3380, 3331, 3236, 3134, 3120, 3027, 2971, 1728, 1597, 1573, 1527, 1490, 1438, 1381, 1292, 1265, 1241, 1187, 1157, 1118, 1080, 1063, 1025, 983, 913, 877, 828, 748, 688, 633 **ESI-MS** *m/z* calcd for $C_{12}H_{10}IN_3O$ $[M + H]^+$ 339.9, found 340.0 **Melting point**: 177-179°C

1-(3-iodopyridin-4-yl)-3-phenylurea

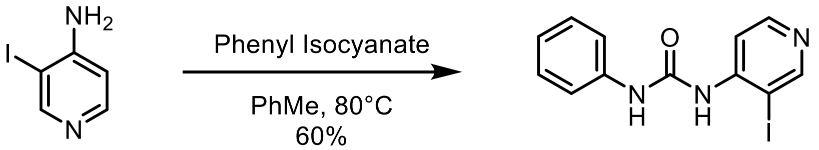

Synthesized according to the general procedure by the reaction of 4-amino-3-iodopyridine (100 mg, 0.454 mmol) with phenyl isocyanate. It was then purified by column chromatography (50% to 80% EtOAc in heptane, silica gel) to obtain 1-(3-iodopyridin-4-yl)-3-phenylurea (92 mg, 0.27 mmol, 60%) as a white solid.

^{1}H NMR (400 MHz, DMSO-d_6) δ = 9.87 (br. s., 1 H), 8.76 (d, *J* = 0.2 Hz, 1 H), 8.32 (d, *J* = 5.6 Hz, 1 H), 8.14 (br. s, 1 H), 8.10 (dd, *J* = 0.2, 5.6 Hz, 1 H), 7.51 - 7.45 (m, 2 H), 7.36 - 7.29 (m, 2 H), 7.07 - 7.00 (m, 1 H) **^{13}C NMR** (101 MHz, DMSO-d_6) δ = 157.5, 151.6, 149.2, 146.8, 138.9, 128.9, 122.7, 118.5, 114.4, 87.1 **FT-IR (**ATR, neat, cm^{-1}) 3337, 3273, 3203, 3008, 2952, 2871, 2810, 1716, 1629, 1600, 1581, 1555, 1490, 1444, 1406, 1326, 1299, 1251, 1194, 1109, 1081, 1038, 1014, 903, 846, 833, 751, 722, 687, 637 **ESI-MS** *m/z* calcd for $C_{12}H_{10}IN_3O$ [M + H]$^+$ 339.9, found 339.9 **Melting point:** 162-164 °C

1-(2-methylpyridin-4-yl)-3-phenylurea

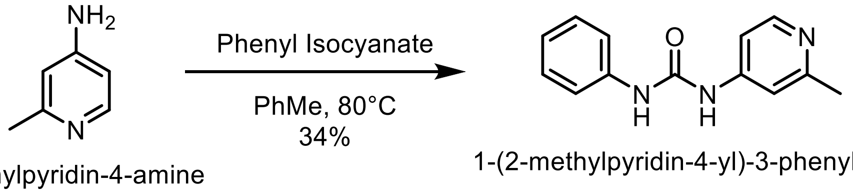

2-methylpyridin-4-amine

1-(2-methylpyridin-4-yl)-3-phenylurea

Synthesized according to the general procedure by the reaction of 2-methylpyridin-4-amine (502 mg, 4.64 mmol) with phenyl isocyanate. It was then purified by column chromatography (50% to 80% EtOAc in heptane, silica gel) to obtain 1-(3-bromopyridin-4-yl)-3-phenylurea (355 mg, 1.56 mmol, 34%) as a white solid.

^{1}H NMR (400 MHz, Acetone-d_6) δ = 8.31 (dd, *J* = 0.7, 5.4 Hz, 1 H), 7.58 - 7.48 (m, 2 H), 7.42 - 7.34 (m, 2 H), 7.33 - 7.17 (m, 2 H), 7.08 - 6.93 (m, 1 H), 2.43 (s, 3 H) **^{13}C NMR** (101 MHz, Acetone-d_6) δ = 159.6, 153.1, 150.1, 148.2, 139.8, 129.6, 123.6, 119.9, 112.5, 111.0, 24.4 **FT-IR (**ATR, neat, cm^{-1}) 3551, 3284, 3194, 3134, 3025, 2985, 2921, 2619, 2380, 2348, 1677, 1587, 1553, 1524, 1495, 1463, 1443, 1398, 1306, 1286, 1251, 1207, 1176, 1157, 1114, 1084, 1059, 1047, 1027, 1000, 960, 905, 875, 858, 837, 826, 786, 750, 724, 692, 643 **ESI-MS** *m/z* calcd for $C_{13}H_{13}N_3O$ [M + H]$^+$ 227.1, found 228.1 **Melting point:** 128 – 132 °C

1-phenyl-3-(2-(trifluoromethyl)pyridin-4-yl)urea

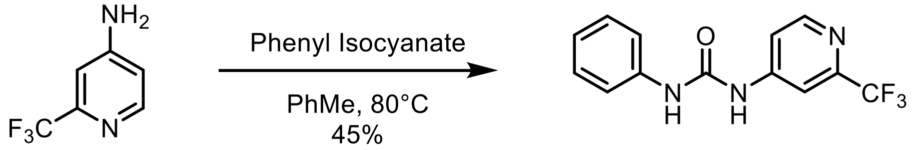

Synthesized according to the general procedure by the reaction of 2-(trifluoromethyl)pyridin-4-amine (450 mg, 2.8 mmol) with phenyl isocyanate. It was then purified by column chromatography (35% to 80% EtOAc in heptane, silica gel) to obtain 1-(3-bromopyridin-4-yl)-3-phenylurea (353 mg, 1.26 mmol, 45%) as a white solid.

^{1}H NMR (400 MHz, DMSO-d$_6$) δ = 9.53 (s, 1 H), 9.03 (s, 1 H), 8.52 (d, *J* = 5.5 Hz, 1 H), 8.05 (d, *J* = 1.8 Hz, 1 H), 7.57 (dd, *J* = 2.0, 5.6 Hz, 1 H), 7.51 - 7.40 (m, 2 H), 7.36 - 7.25 (m, 2 H), 7.12 - 6.93 (m, 1 H) **^{13}C NMR** (101 MHz, DMSO-d$_6$) δ = 152.0, 150.7, 148.3, 147.3 (d, *J* = 33.0 Hz), 138.7, 128.8, 122.8, 118.9, 121.7 (q, *J* = 274.3 Hz), 114.8, 108.8 (d, *J* = 2.9 Hz) **FT-IR (**ATR, neat, cm^{-1}) 3390, 3347, 3265, 3168, 3090, 3029, 1730, 1590, 1529, 1500, 1485, 1442, 1343, 1307, 1279, 1245, 1225, 1185, 1174, 1131, 1118, 1078, 1041, 1026, 993, 937, 895, 842, 753, 686, 638, 615 cm^{-1} **ESI-MS** *m/z* calcd for $C_{13}H_{10}F_3N_3O$ [M + H]$^+$ 282.1, found 282.0 **Melting point:** 166 - 168 °C

1-(2-methoxypyridin-4-yl)-3-phenylurea

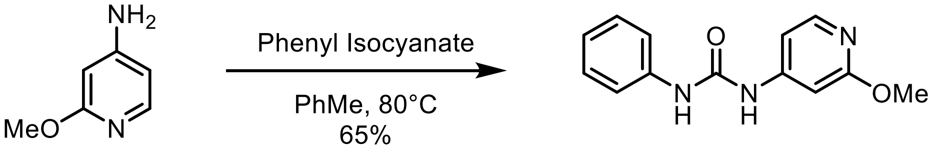

Synthesized according to the general procedure by the reaction of 2-methoxypyridin-4-amine (500 mg, 4.02 mmol) with phenyl isocyanate. It was then purified by column chromatography (50% to 80% EtOAc in heptane, silica gel) to obtain 1-(2-methoxypyridin-4-yl)-3-phenylurea (637 mg, 2.61 mmol, 65%) as a white solid.

^{1}H NMR (400 MHz, Acetone-d$_6$) δ = 8.41 (br. s., 1 H), 8.22 (br. s., 1 H), 7.94 (d, *J* = 5.7 Hz, 1 H), 7.55 - 7.49 (m, 2 H), 7.33 - 7.24 (m, 2 H), 7.07 (d, *J* = 1.7 Hz, 1 H), 7.04 - 6.97 (m, 2 H), 3.85 (s, 3 H) **^{13}C NMR** (101 MHz, Acetone-d$_6$) δ = 166.7, 153.4, 150.3, 148.5, 140.8, 130.2, 124.1, 120.3, 108.9, 99.0, 53.9 **FT-IR (**ATR, neat, cm^{-1}) 3327, 3299, 3192, 1658, 1583, 1526, 1496, 1456, 1396, 1342, 1314, 1297, 1221, 1187, 1171, 1110, 1038, 989, 952, 914, 858, 823, 793, 747, 731, 692, 649, 637, 617 **ESI-MS** *m/z* calcd for $C_{13}H_{13}N_3O_2$ [M + H]$^+$ 244.1, found 244.0 **Melting point**: 126 – 127 °C

References

Blum, W., Pecze, L., Rodriguez, J.W., Steinauer, M., and Schwaller, B. (2018). Regulation of calretinin in malignant mesothelioma is mediated by septin 7 binding to the CALB2 promoter. *BMC Cancer* 18**,** 475.

Jin, X., and Davies, R.P. (2017). Copper-Catalysed Aromatic-Finkelstein Reactions with Amine-Based Ligand Systems. . *Catal. Sci. Technol.* 7**,** 2110–2117.

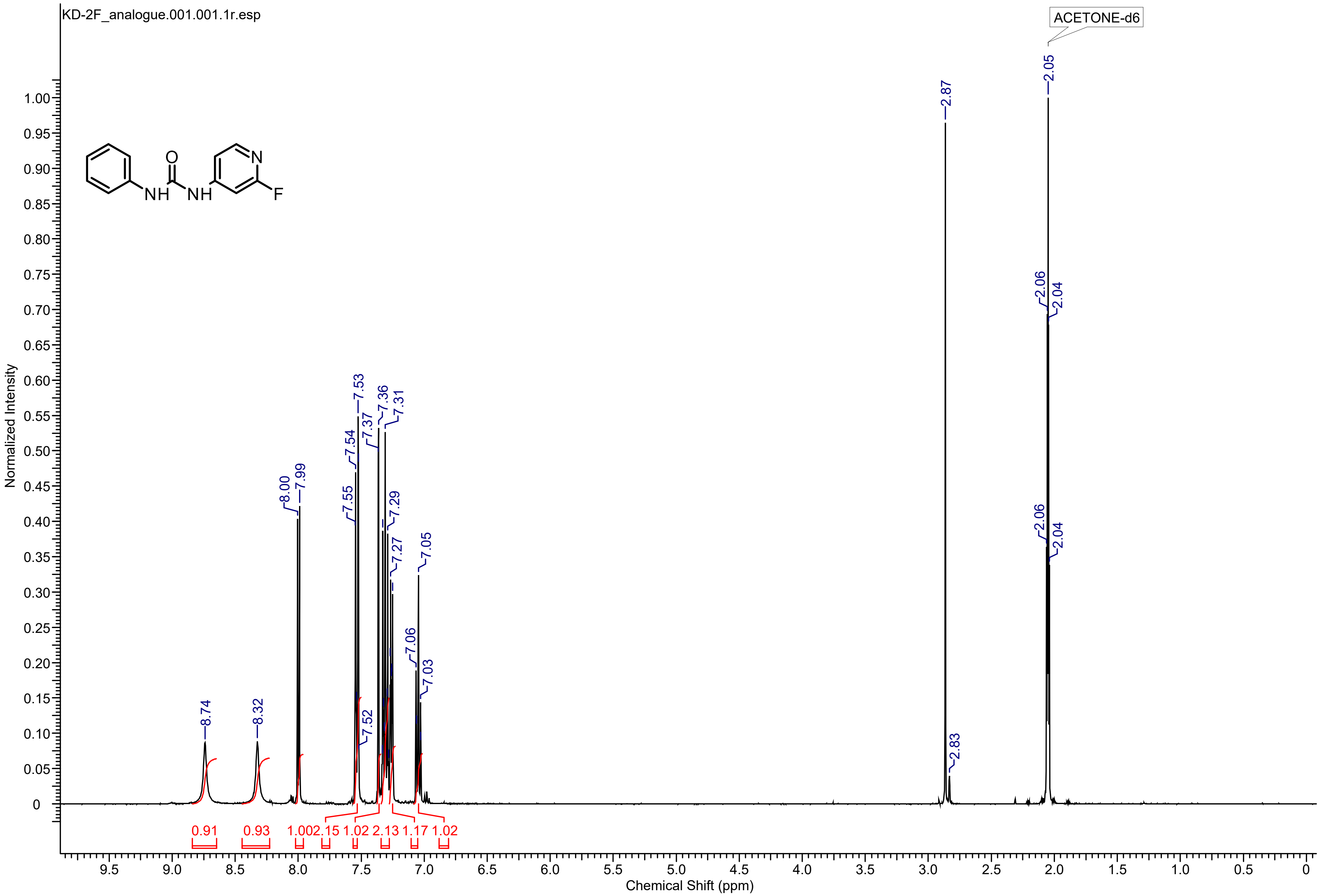
KD-2F_analogue.001.001.1r.esp
ACETONE-d6
NH
NH
O
N
F
8.74
8.32
8.00
7.99
7.55
7.54
7.53
7.52
7.37
7.36
7.31
7.29
7.27
7.06
7.05
7.03
2.87
2.83
2.06
2.05
2.04
0.91
0.93
1.00
2.15
1.02
2.13
1.17
1.02
Normalized Intensity
Chemical Shift (ppm)

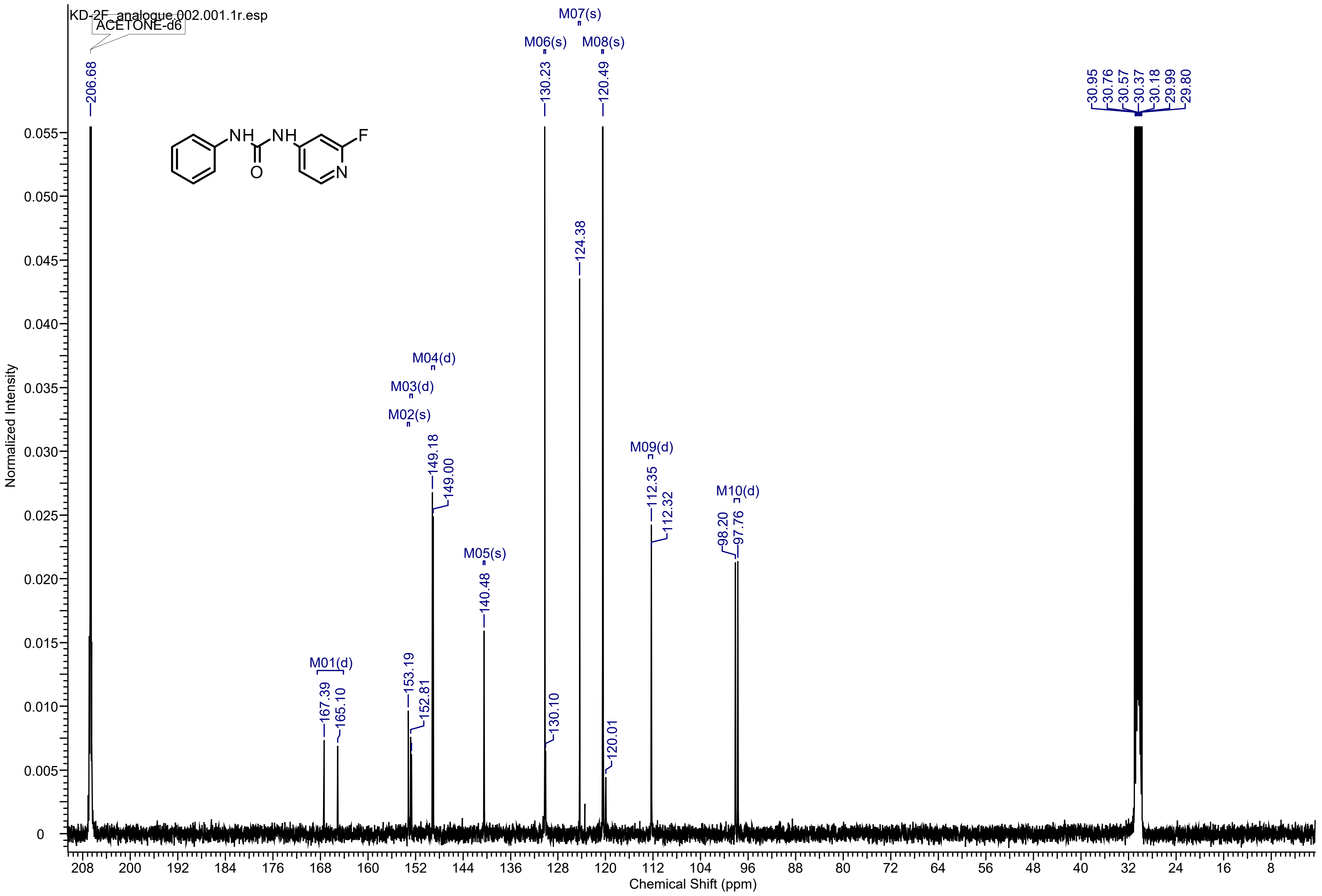

KD-2F_analogue.002.001.1r.esp
ACETONE-d6
206.68
M06(s)
130.23
M07(s)
124.38
M08(s)
120.49
30.95
30.76
30.57
30.37
30.18
29.99
29.80
M04(d)
M03(d)
M02(s)
149.18
149.00
M05(s)
140.48
M09(d)
112.35
112.32
M10(d)
98.20
97.76
M01(d)
167.39
165.10
153.19
152.81
130.10
120.01
NH
NH
O
F
N
Normalized Intensity
Chemical Shift (ppm)

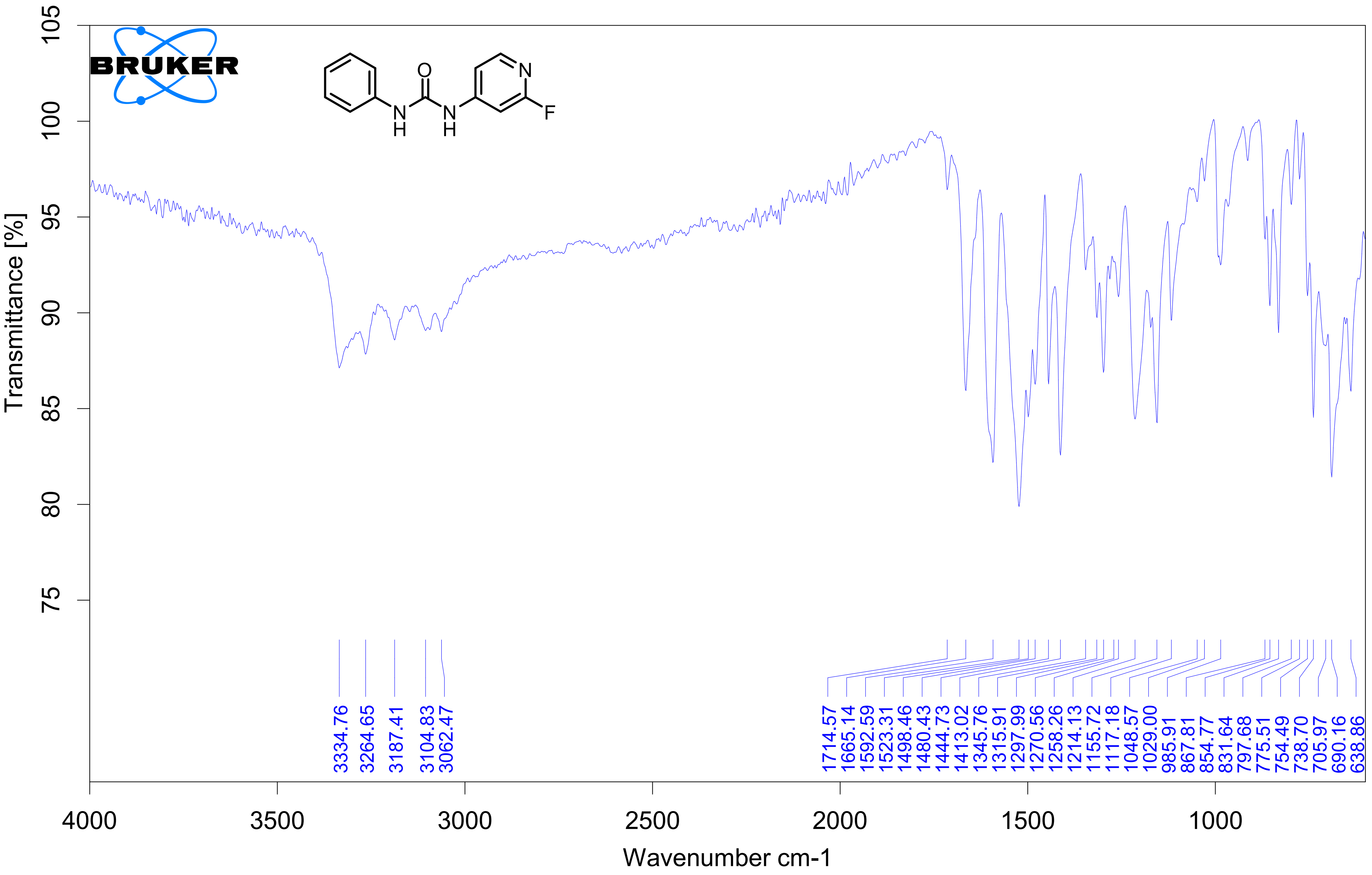

C:\DATA\User\Kim\KD-2F-analogue.0 KD-2F-analogue solid	05.07.2021

ESI-MS

Analysis Info

Acquisition Date 05.07.2021 17:16:36

Analysis Name	D:\Data\Kim\FCF_09.d
Method	AA_Standard_MS_2015.m
Sample Name	FCF_09
Comment	in MeOH

Instrument: BRUKER - Ion Trap MS esquire HCT

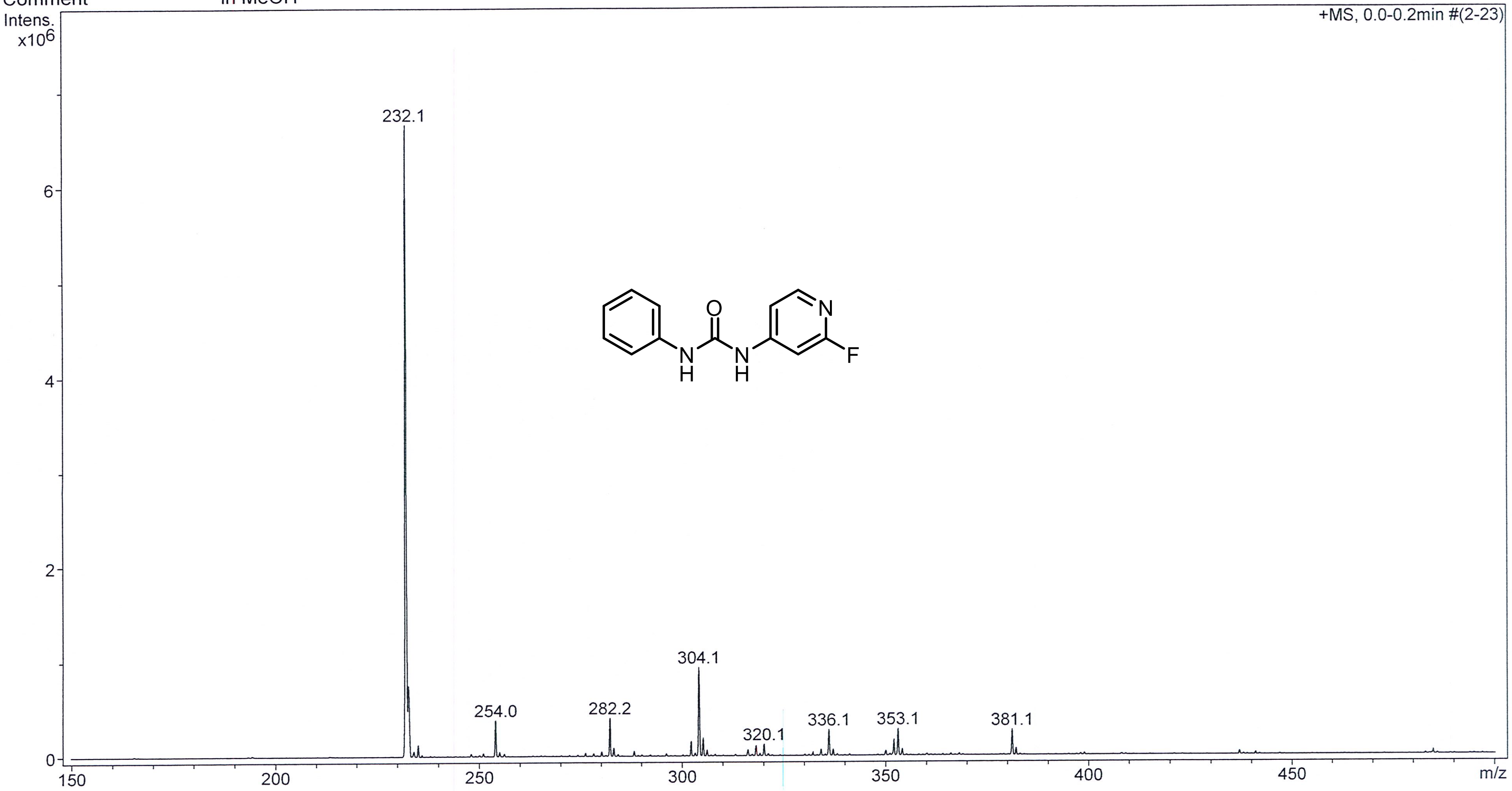

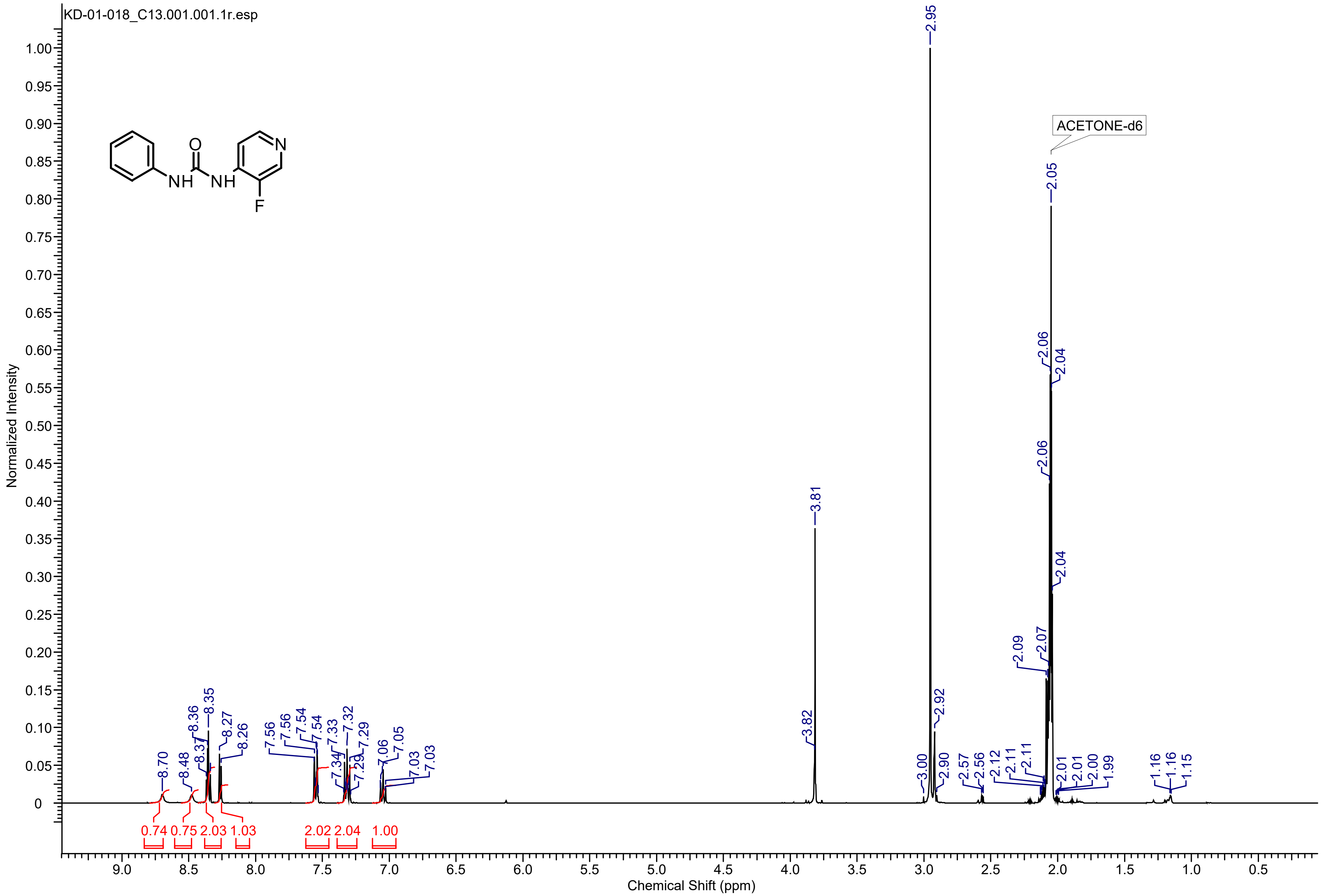

KD-01-018_C13.001.001.1r.esp
ACETONE-d6
Normalized Intensity
Chemical Shift (ppm)
NH
NH
O
N
F

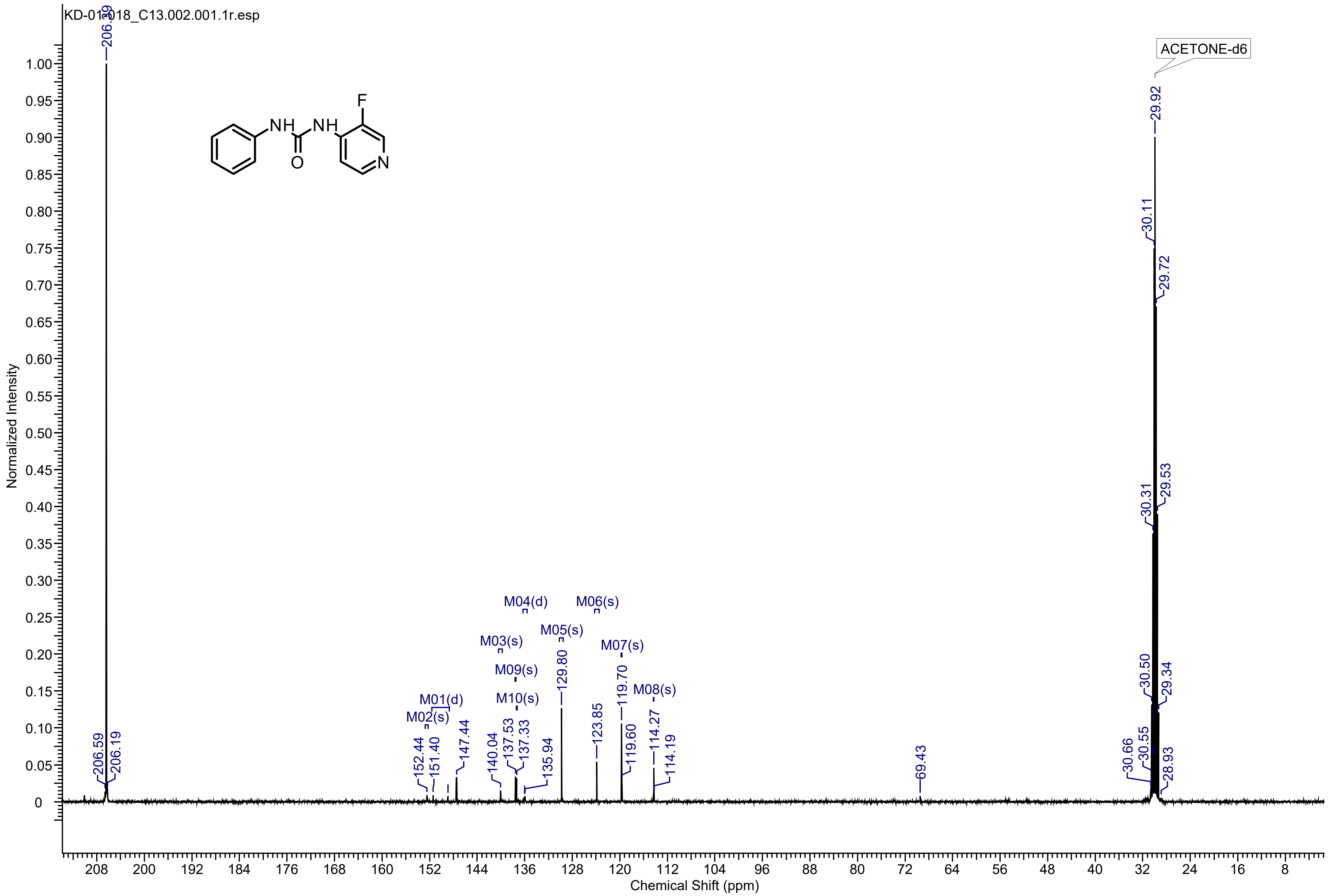
KD-01-018_C13.002.001.1r.esp
ACETONE-d6
NH
NH
F
O
N
206.39
206.59
206.19
152.44
151.40
147.44
140.04
137.53
137.33
135.94
129.80
123.85
119.70
119.60
114.27
114.19
69.43
30.66
30.55
30.50
30.31
30.11
29.92
29.72
29.53
29.34
28.93
M01(d)
M02(s)
M03(s)
M04(d)
M05(s)
M06(s)
M07(s)
M08(s)
M09(s)
M10(s)
Normalized Intensity
Chemical Shift (ppm)

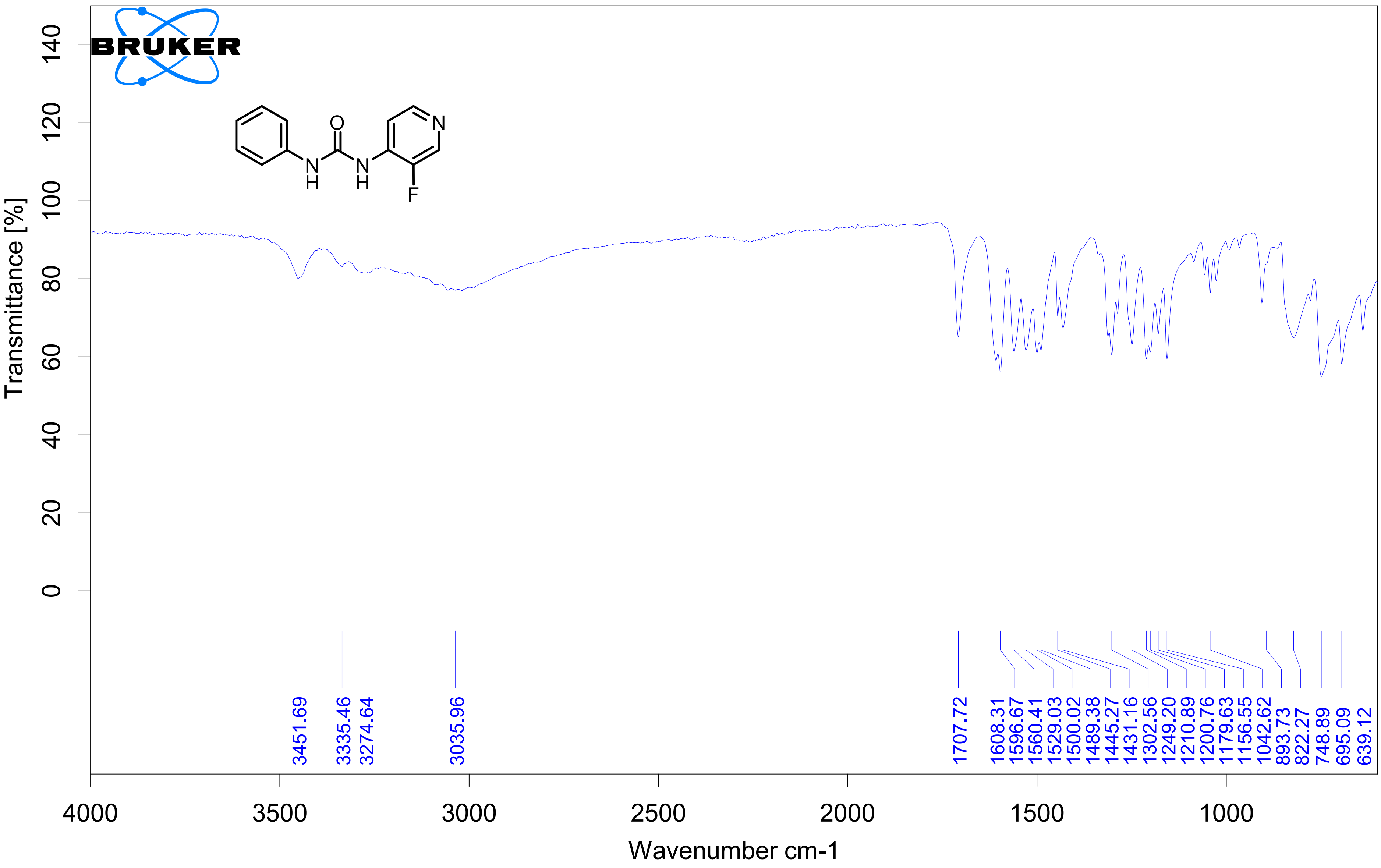

C:\DATA\User\Kim\KD-3F-analogue.0	KD-3F-analogue	solid	05.07.2021

ESI-MS

Analysis Info

Analysis Name	D:\Data\Kim\3F-analogue_2.d
Method	AA_Standard_MS_2015.m
Sample Name	3F-analogue_2
Comment	in MeOH

Acquisition Date 19.05.2021 16:45:06

Instrument: BRUKER - Ion Trap MS esquire HCT

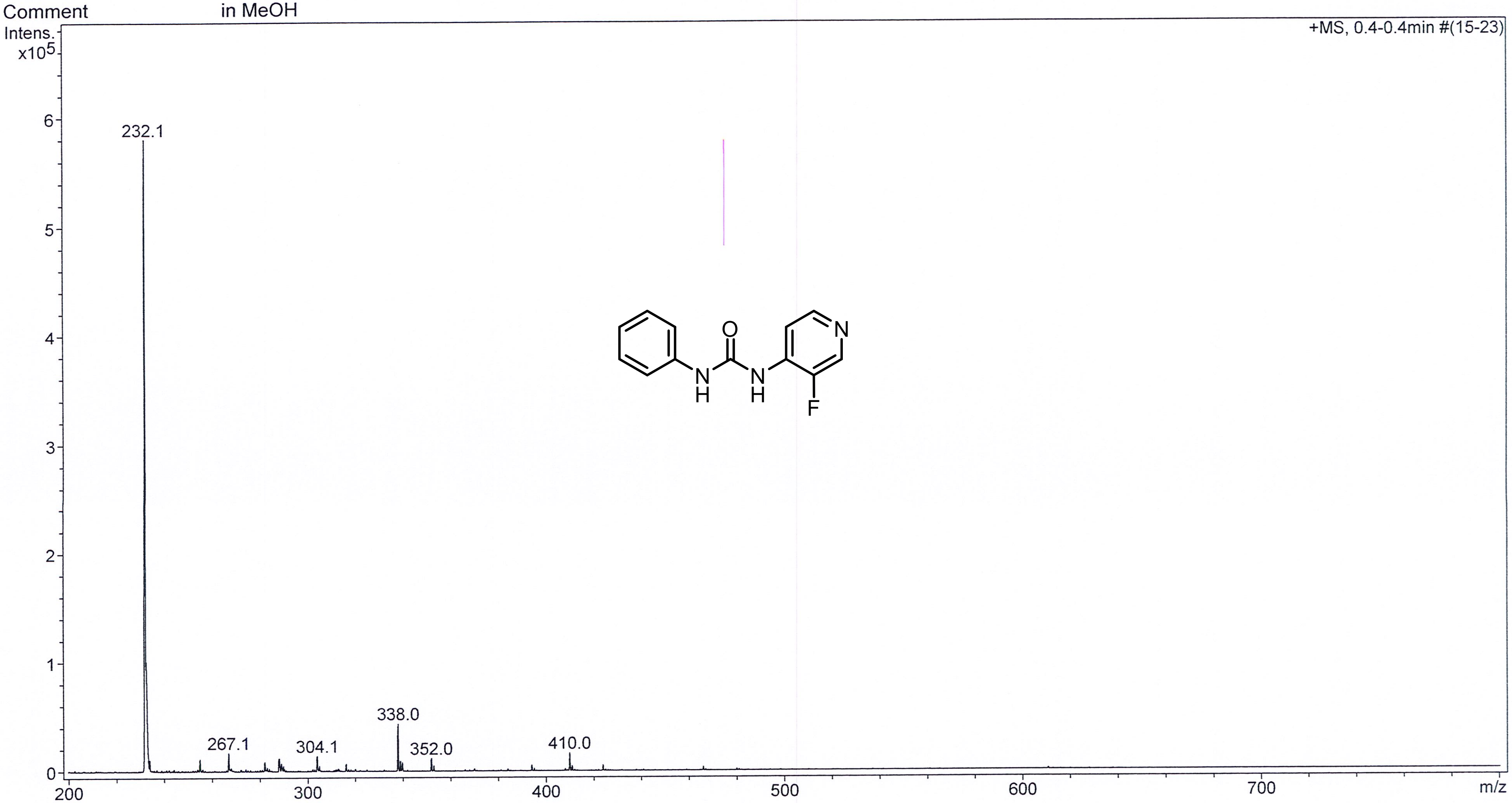

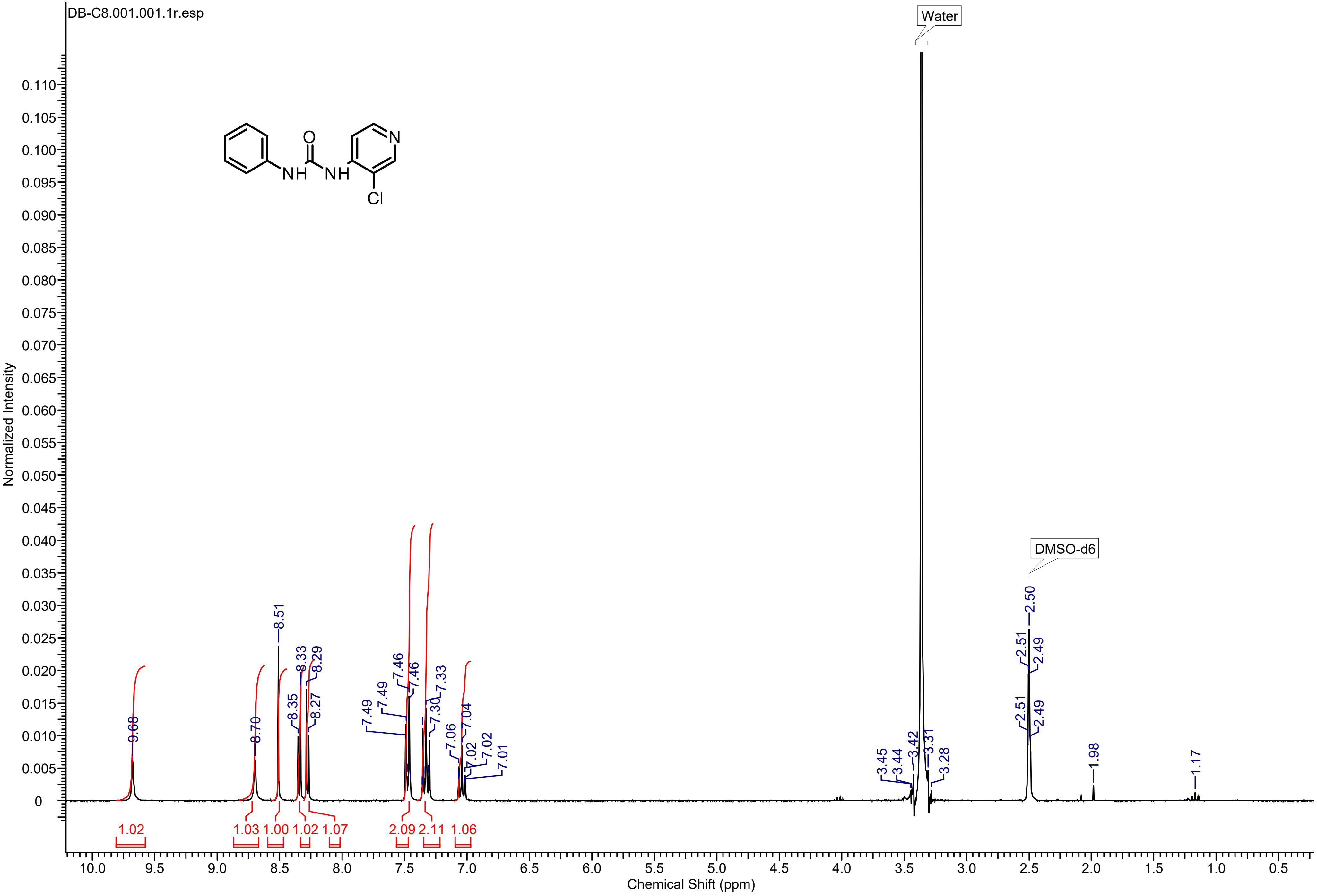
DB-C8.001.001.1r.esp
Water
DMSO-d6
NH
NH
O
N
Cl
Normalized Intensity
Chemical Shift (ppm)
9.68
8.70
8.51
8.35
8.33
8.29
8.27
7.49
7.49
7.46
7.46
7.33
7.30
7.06
7.04
7.02
7.02
7.01
3.45
3.44
3.42
3.31
3.28
2.51
2.51
2.50
2.49
2.49
1.98
1.17
1.02
1.03
1.00
1.02
1.07
2.09
2.11
1.06

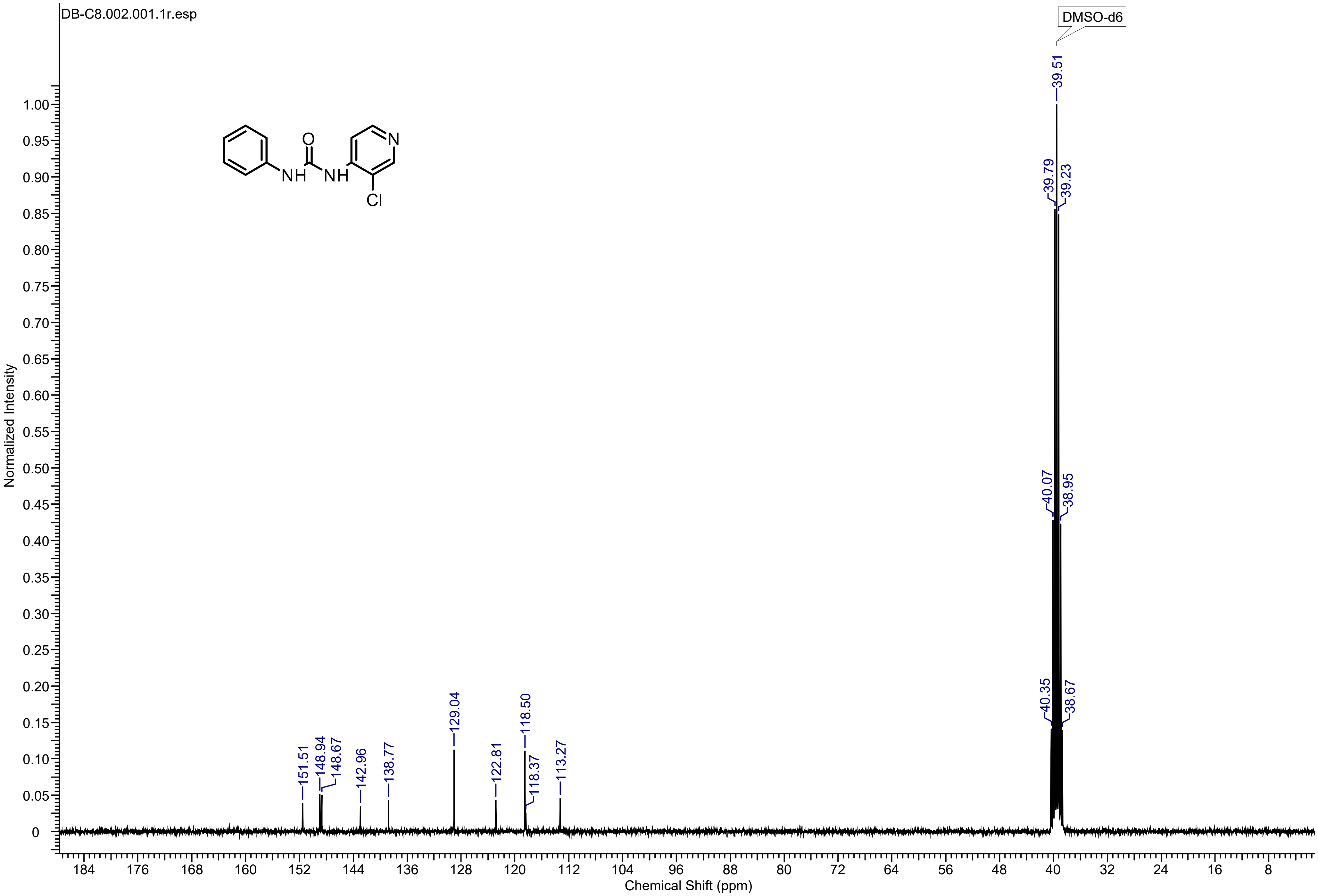
DB-C8.002.001.1r.esp
DMSO-d6
39.51
39.79
39.23
40.07
38.95
40.35
38.67
151.51
148.94
148.67
142.96
138.77
129.04
122.81
118.50
118.37
113.27
NH
NH
O
N
Cl
Normalized Intensity
Chemical Shift (ppm)

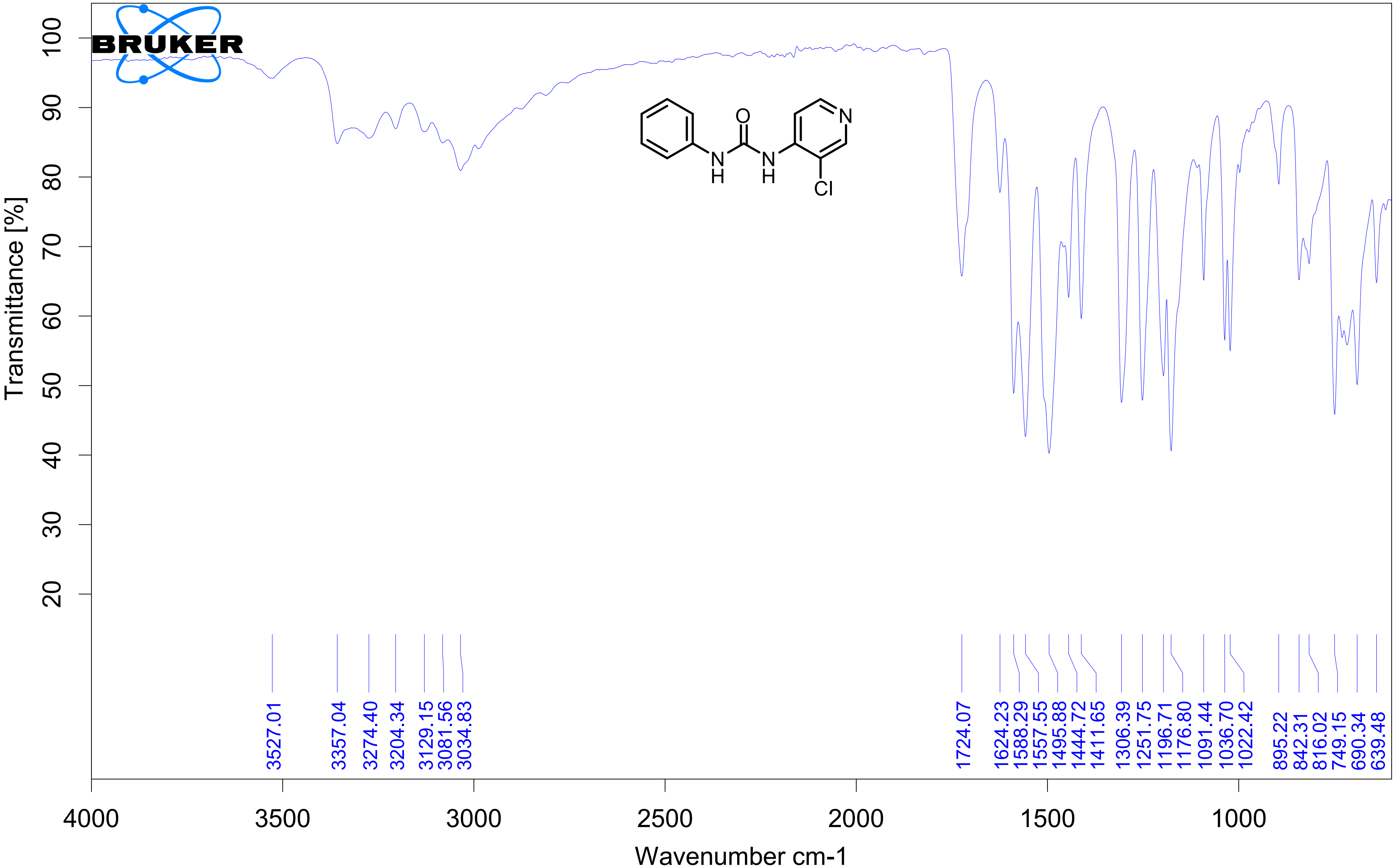

C:\DATA\User\Kim\C8.0 C8 solid	27.08.2019

ESI-MS

Analysis Info

Acquisition Date 05.07.2021 17:14:42

Analysis Name D:\Data\Kim\FCF_08.d
Method AA_Standard_MS_2015.m
Sample Name FCF_08
Comment in MeOH

Instrument: BRUKER - Ion Trap MS esquire HCT

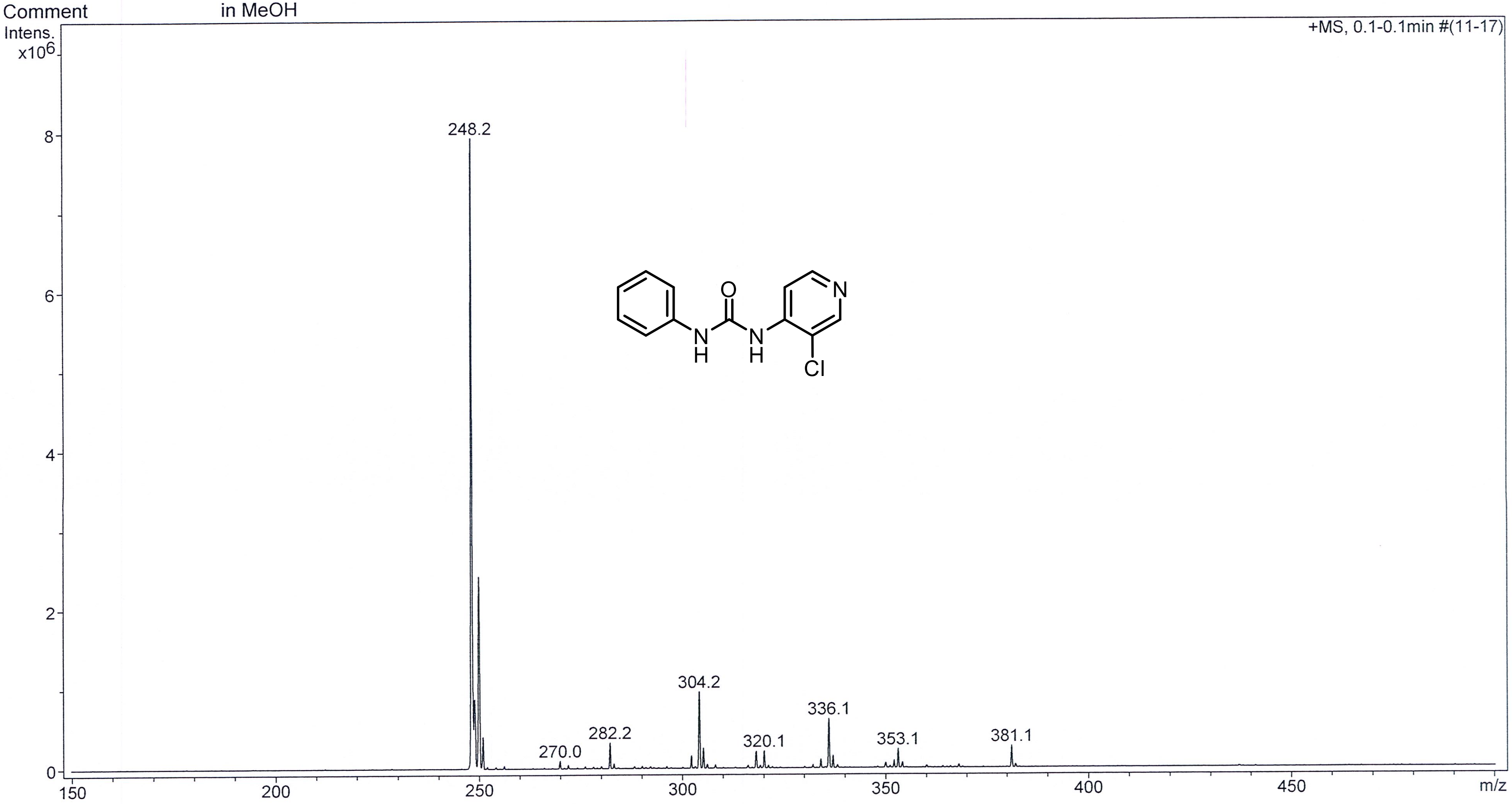

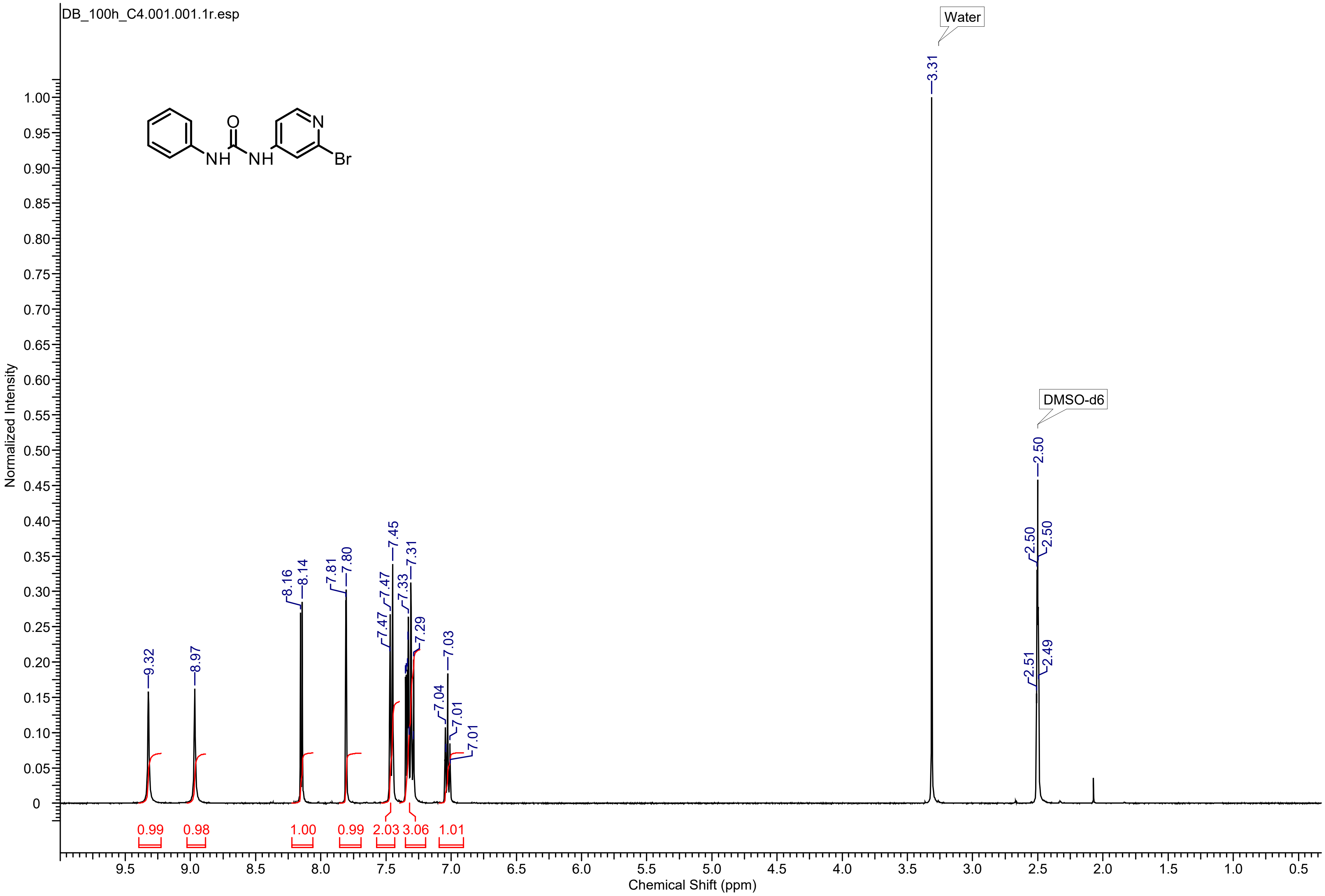
DB_100h_C4.001.001.1r.esp
Water
DMSO-d6
Normalized Intensity
Chemical Shift (ppm)
9.32
8.97
8.16
8.14
7.81
7.80
7.47
7.47
7.45
7.33
7.31
7.29
7.04
7.03
7.01
7.01
3.31
2.51
2.50
2.50
2.50
2.49
0.99
0.98
1.00
0.99
2.03
3.06
1.01
NH
NH
O
N
Br

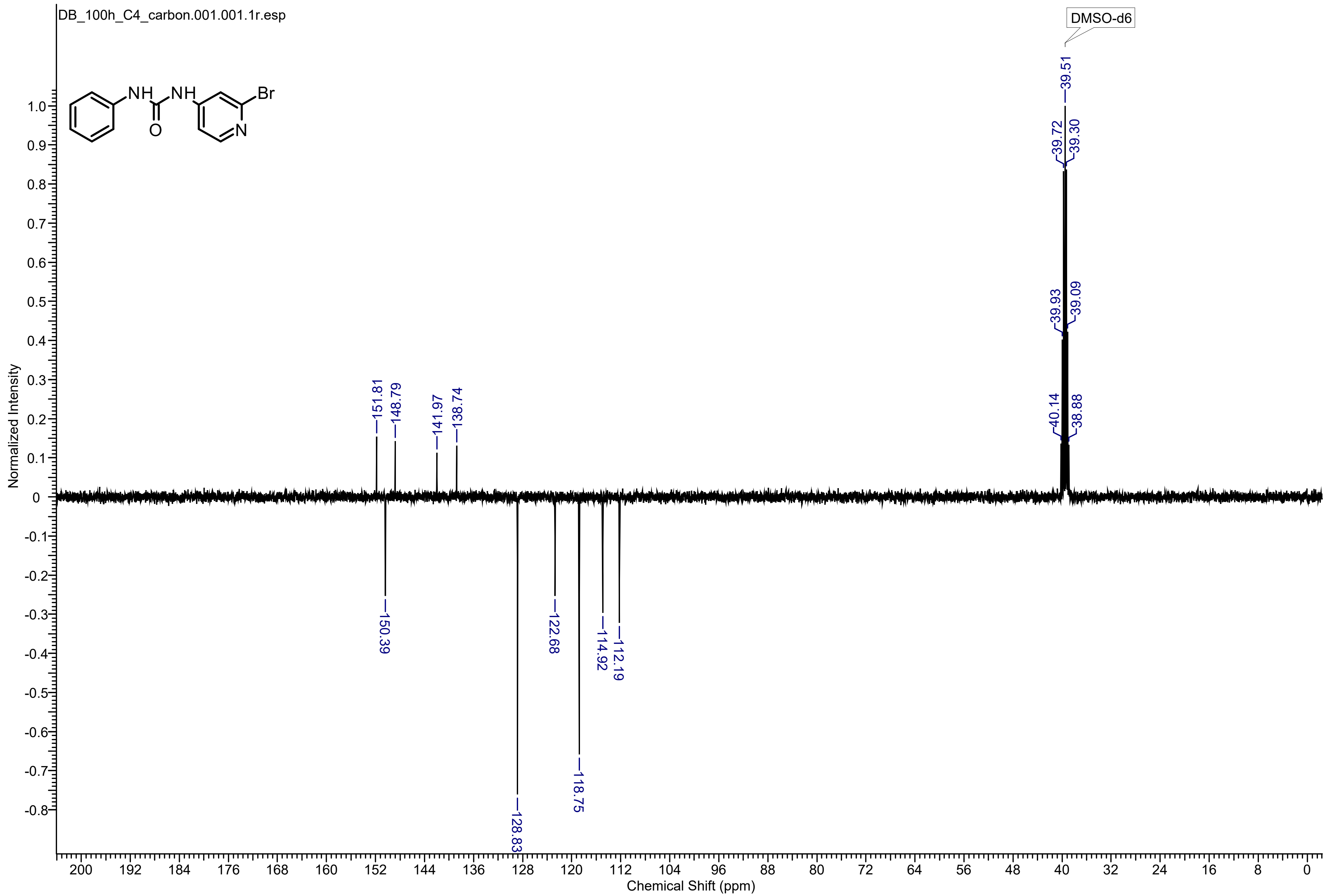

DB_100h_C4_carbon.001.001.1r.esp
DMSO-d6
NH
NH
Br
O
N
151.81
148.79
141.97
138.74
150.39
128.83
122.68
118.75
114.92
112.19
39.51
39.72
39.30
39.93
39.09
40.14
38.88
Normalized Intensity
Chemical Shift (ppm)

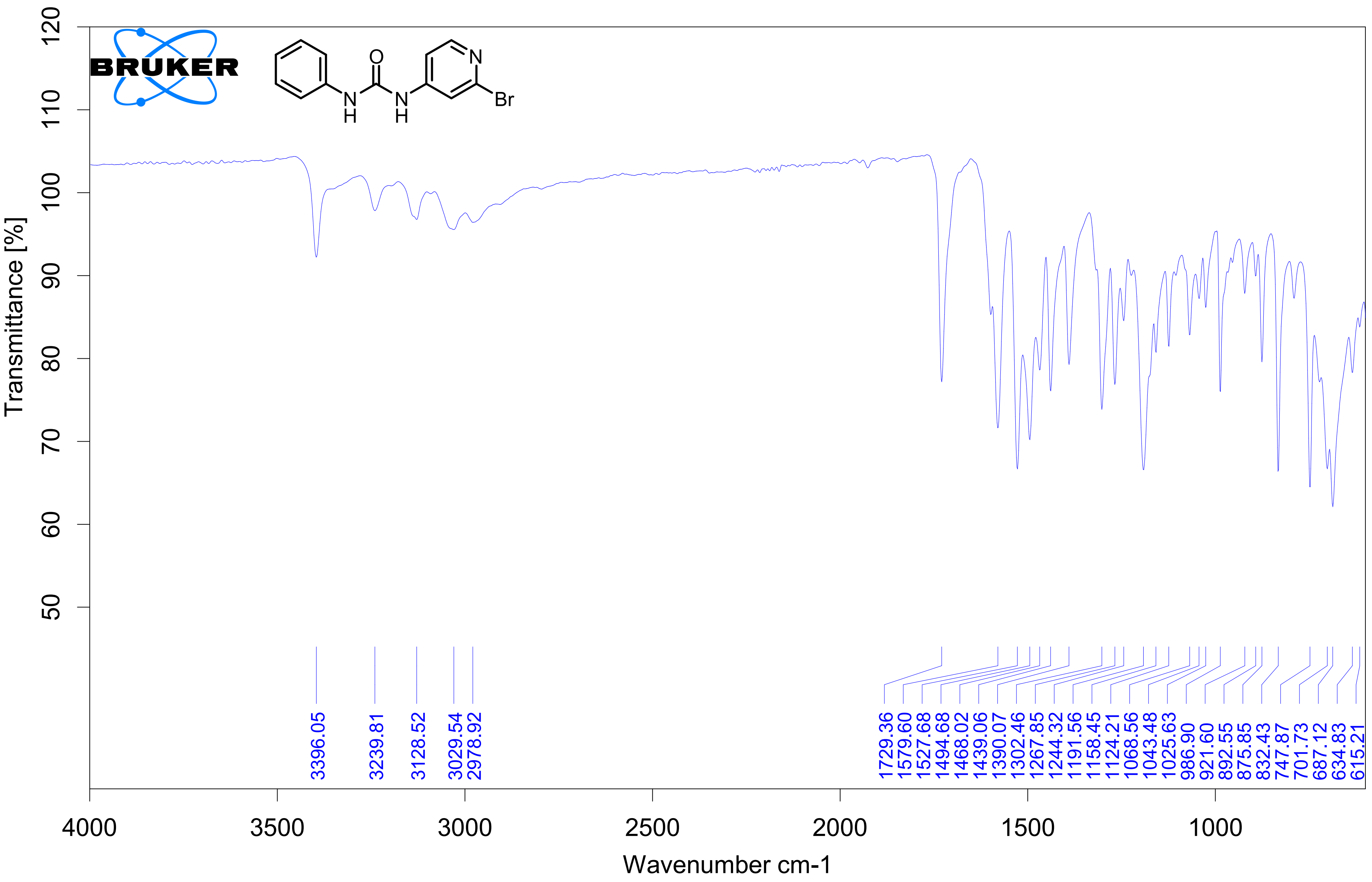

C:\DATA\User\Kim\C4.0	C4	solid	27.08.2019

ESI-MS

Analysis Info

Analysis Name	D:\Data\Kim\FCF_04.d	Acquisition Date	05.07.2021 17:08:55
Method	AA_Standard_MS_2015.m		
Sample Name	FCF_04	Instrument:	BRUKER - Ion Trap MS esquire HCT
Comment	in MeOH		

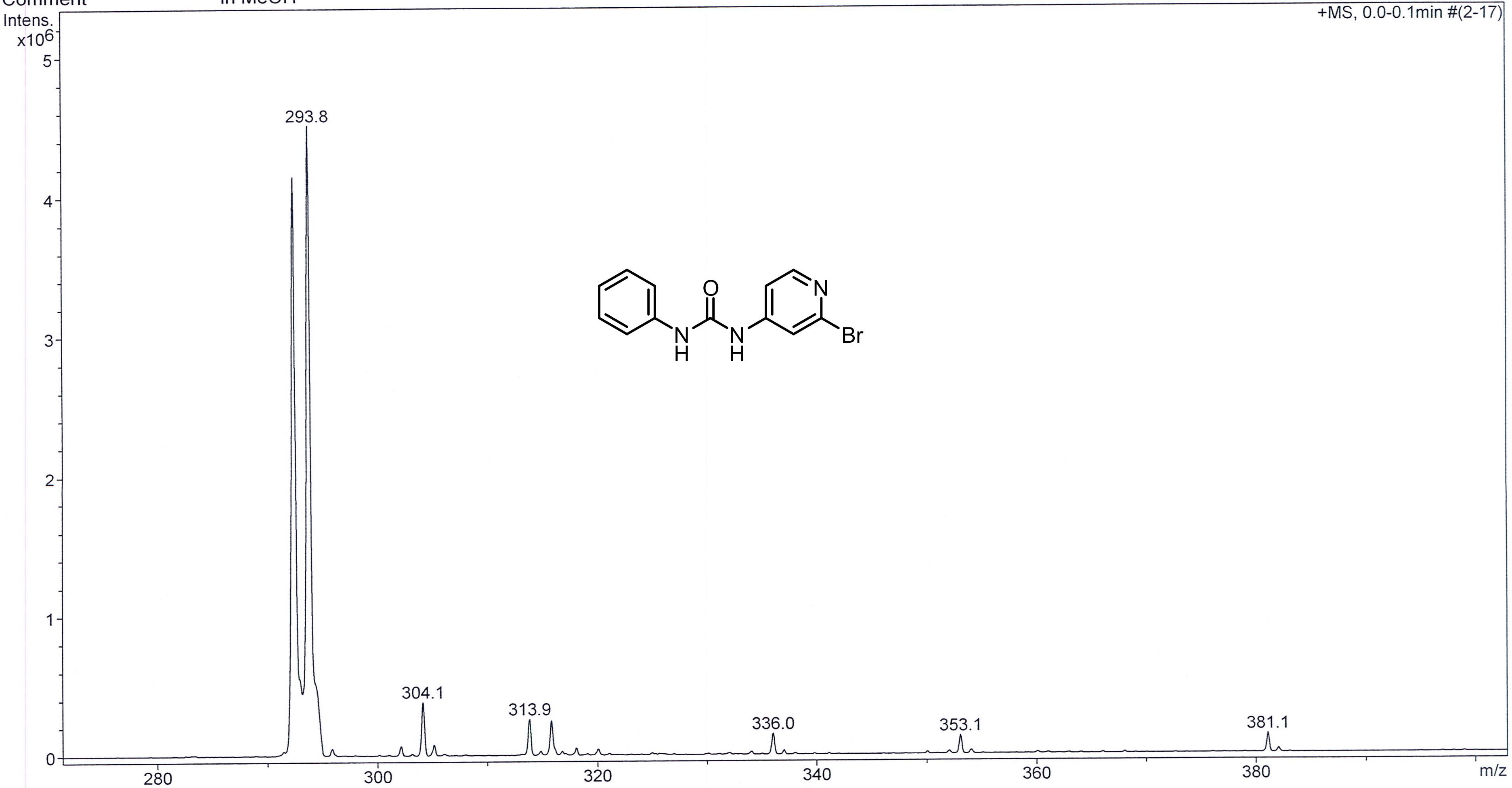

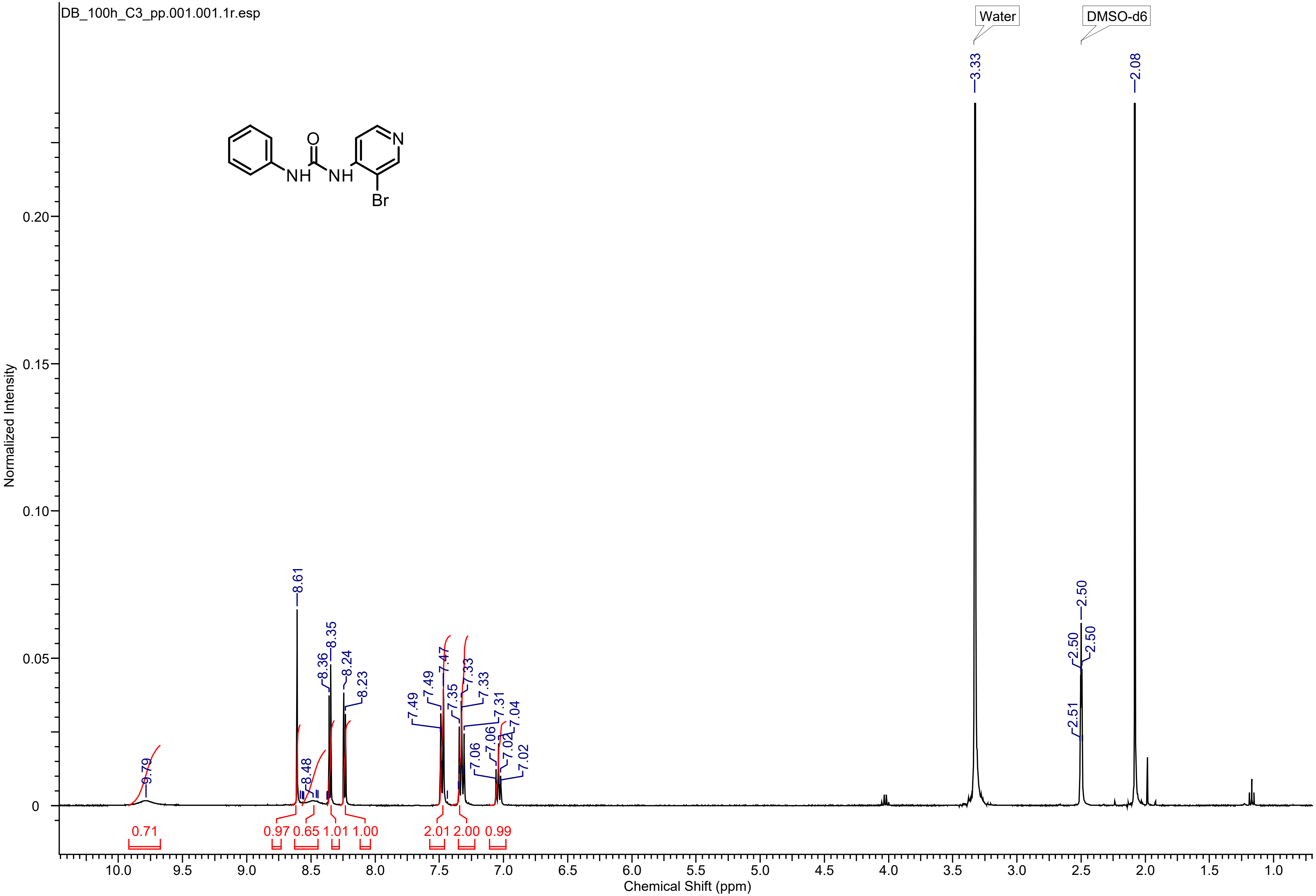
DB_100h_C3_pp.001.001.1r.esp
Water
DMSO-d6
3.33
2.08
NH
NH
O
N
Br
Normalized Intensity
Chemical Shift (ppm)
9.79
8.61
8.48
8.36
8.35
8.24
8.23
7.49
7.49
7.47
7.35
7.33
7.33
7.31
7.06
7.06
7.04
7.04
7.02
2.51
2.50
2.50
2.50
0.71
0.97
0.65
1.01
1.00
2.01
2.00
0.99

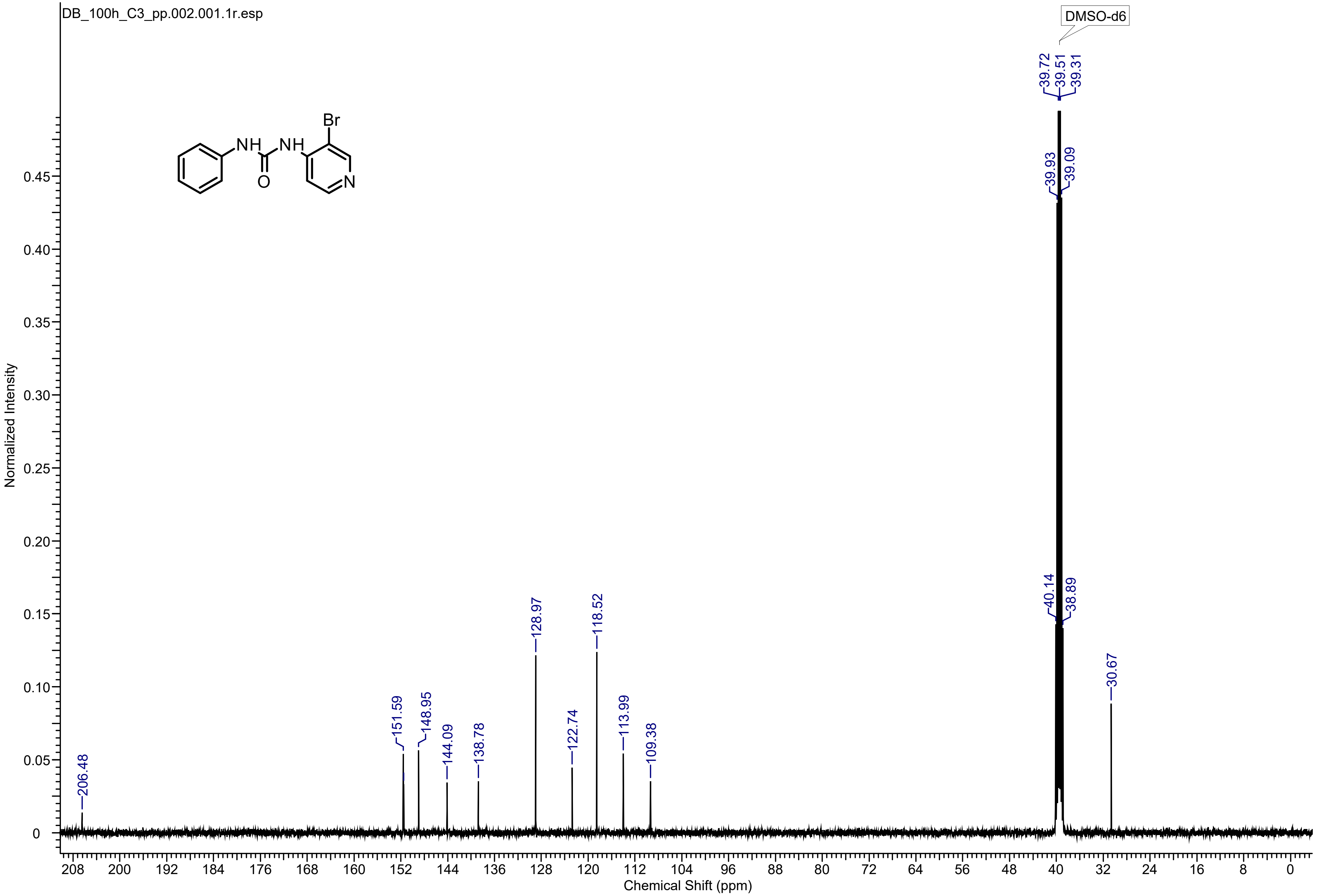

DB_100h_C3_pp.002.001.1r.esp
DMSO-d6
Br
NH
NH
O
N
39.72
39.51
39.31
39.93
39.09
40.14
38.89
206.48
151.59
148.95
144.09
138.78
128.97
122.74
118.52
113.99
109.38
30.67
Normalized Intensity
0.45
0.40
0.35
0.30
0.25
0.20
0.15
0.10
0.05
0
208 200 192 184 176 168 160 152 144 136 128 120 112 104 96 88 80 72 64 56 48 40 32 24 16 8 0
Chemical Shift (ppm)

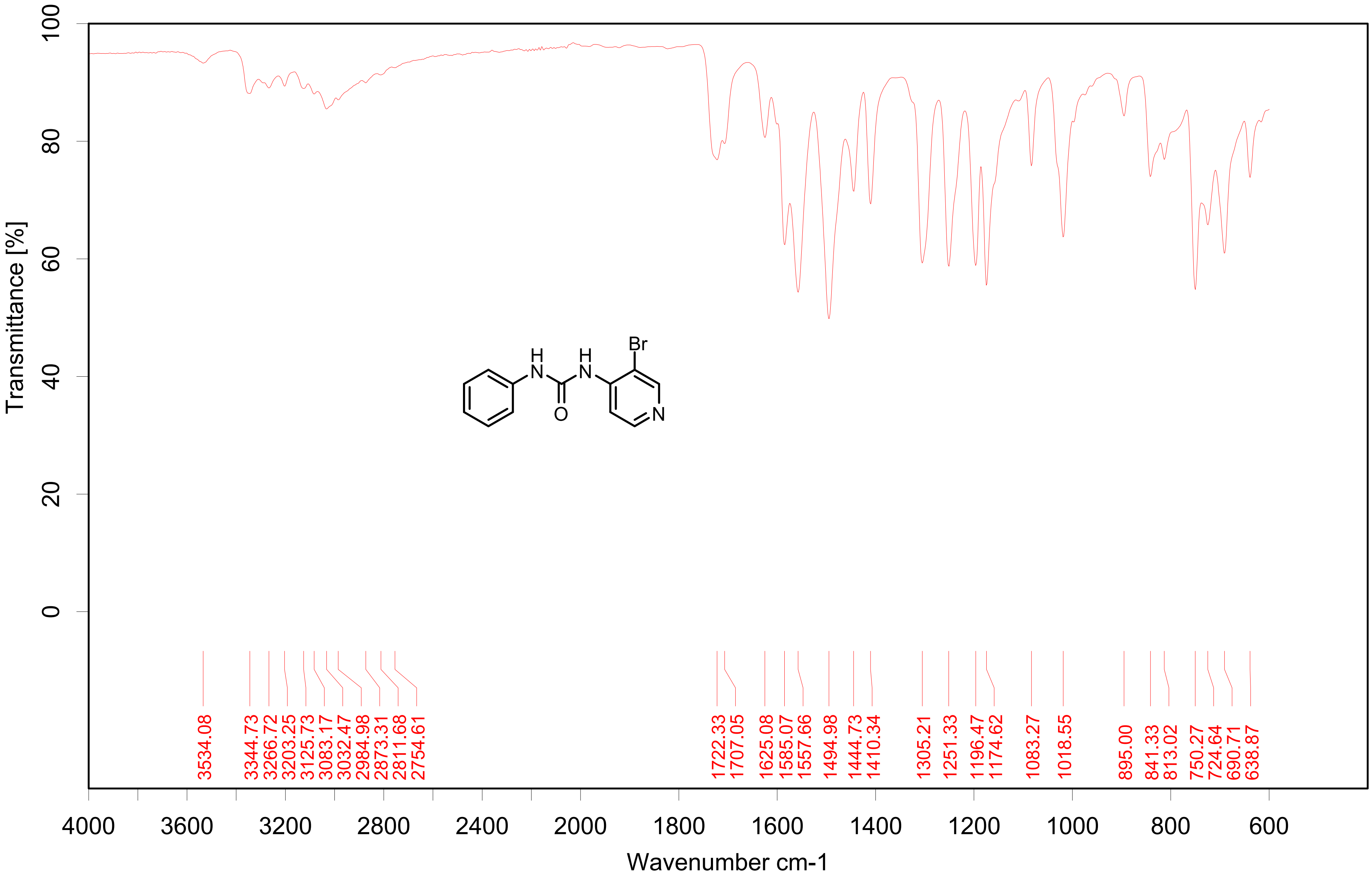

C:\DATA\User\Kim	C3.0	Date: 27.08.2019, 16:29:07

ESI-MS

Analysis Info

Analysis Name	D:\Data\Kim\FCF_03.d	Acquisition Date	05.07.2021 17:06:58
Method	AA_Standard_MS_2015.m		
Sample Name	FCF_03	Instrument:	BRUKER - Ion Trap MS esquire HCT
Comment	in MeOH		

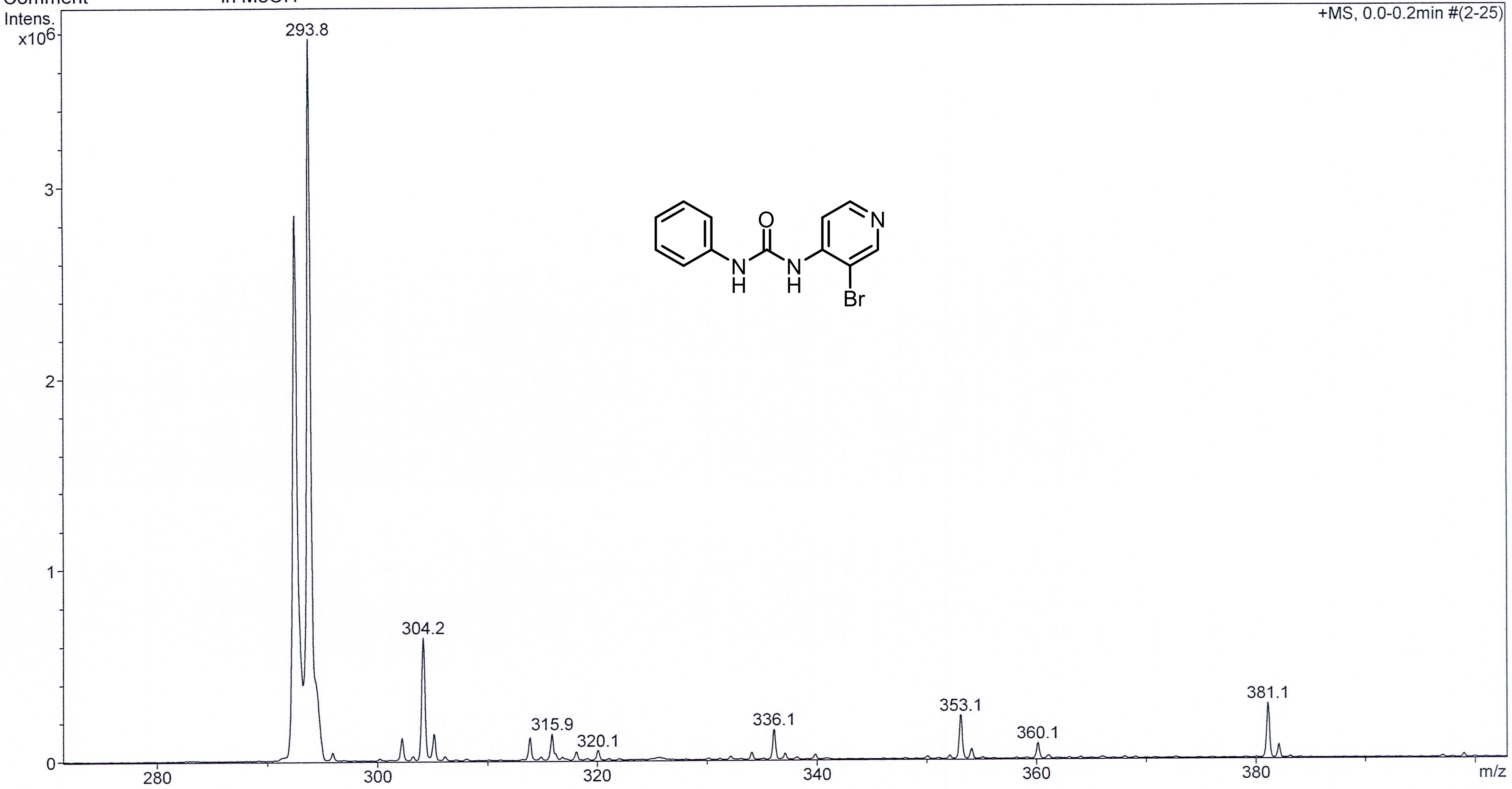

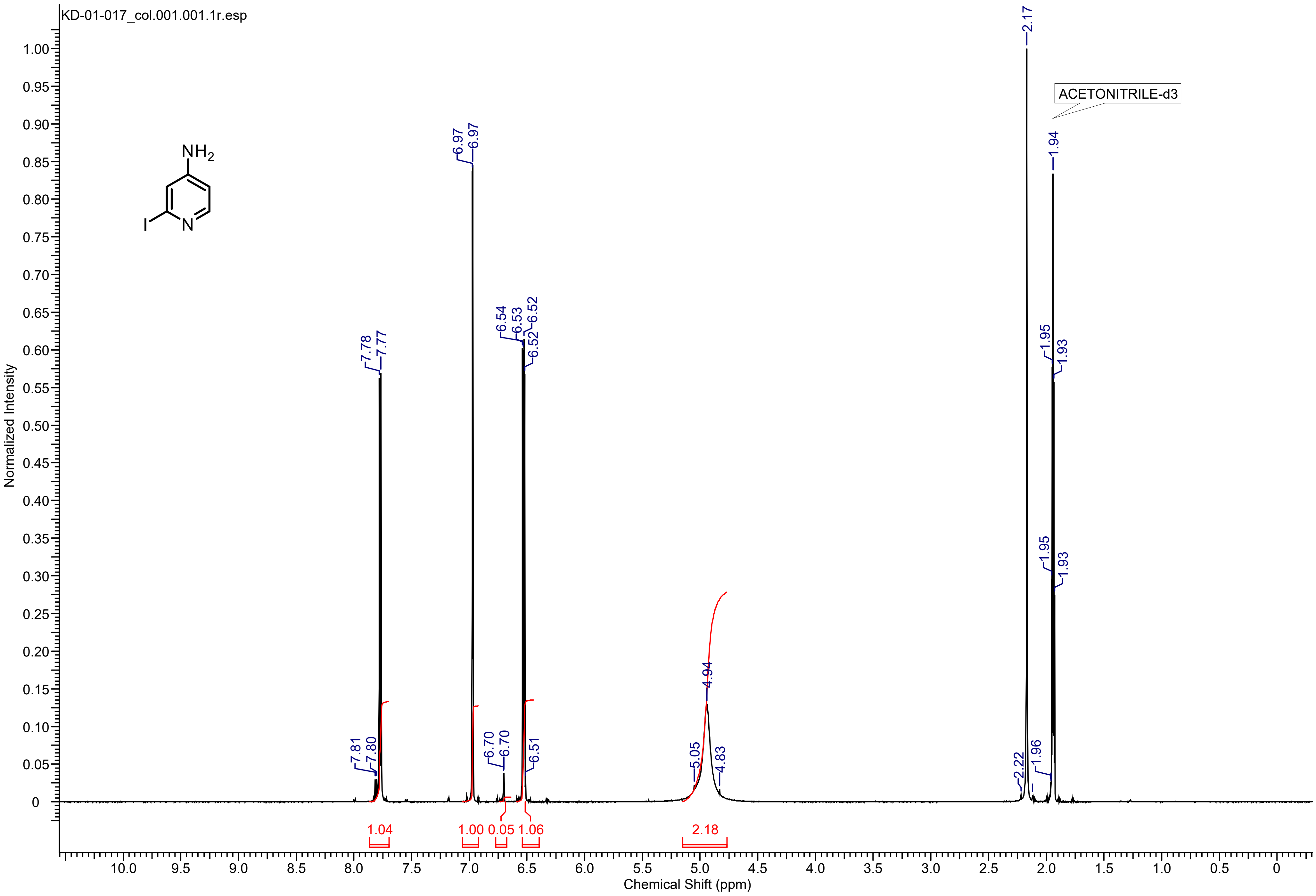

KD-01-017_col.001.001.1r.esp
NH2
I
N
ACETONITRILE-d3
2.17
1.94
6.97
6.97
7.78
7.77
6.54
6.53
6.52
6.52
1.95
1.93
7.81
7.80
6.70
6.70
6.51
5.05
4.94
4.83
2.22
1.96
1.04
1.00
0.05
1.06
2.18
Normalized Intensity
Chemical Shift (ppm)

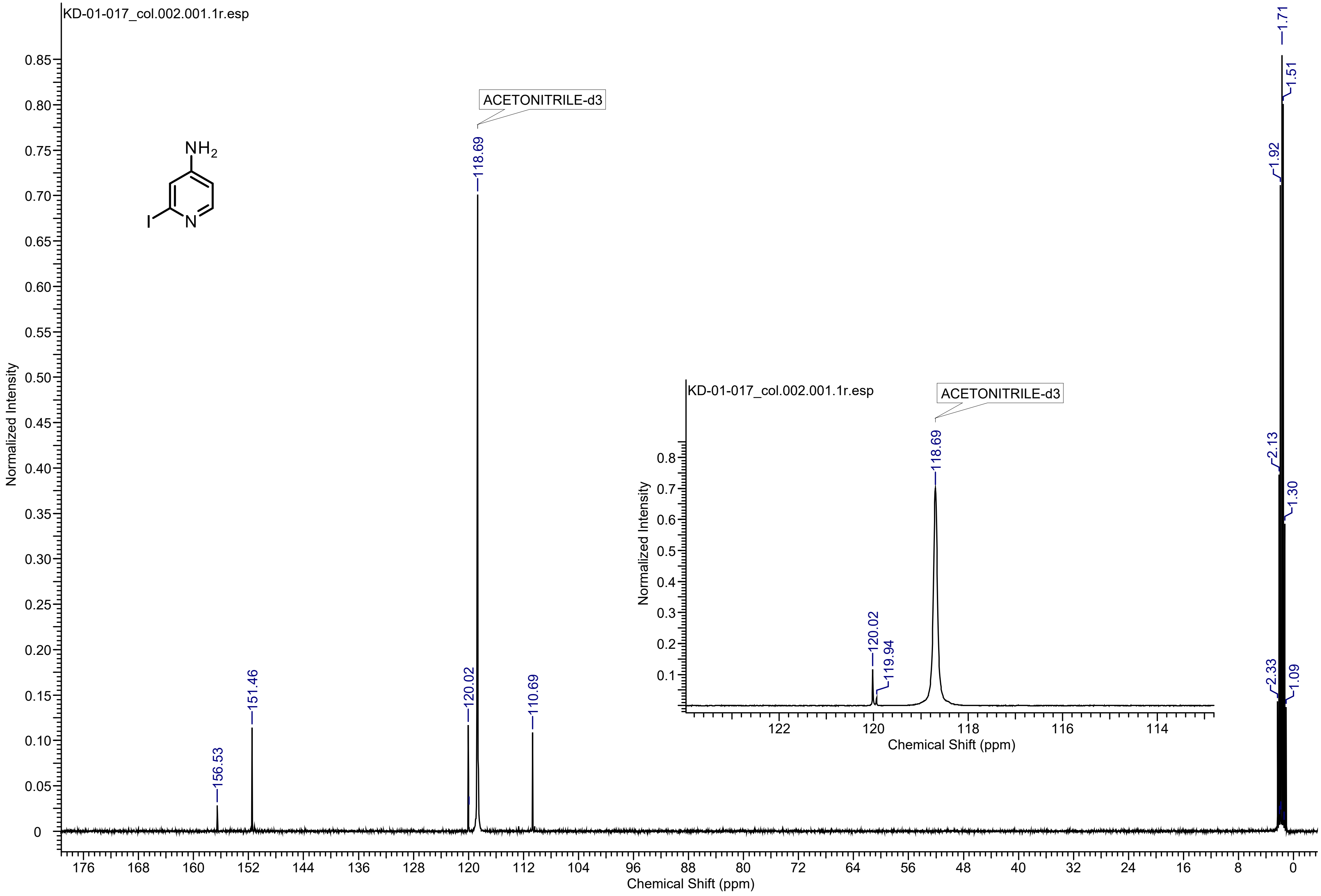

KD-01-017_col.002.001.1r.esp
ACETONITRILE-d3
NH2
I
N
156.53
151.46
120.02
118.69
110.69
2.33
2.13
1.92
1.71
1.51
1.30
1.09
Normalized Intensity
Chemical Shift (ppm)
KD-01-017_col.002.001.1r.esp
ACETONITRILE-d3
120.02
119.94
118.69
Normalized Intensity
Chemical Shift (ppm)

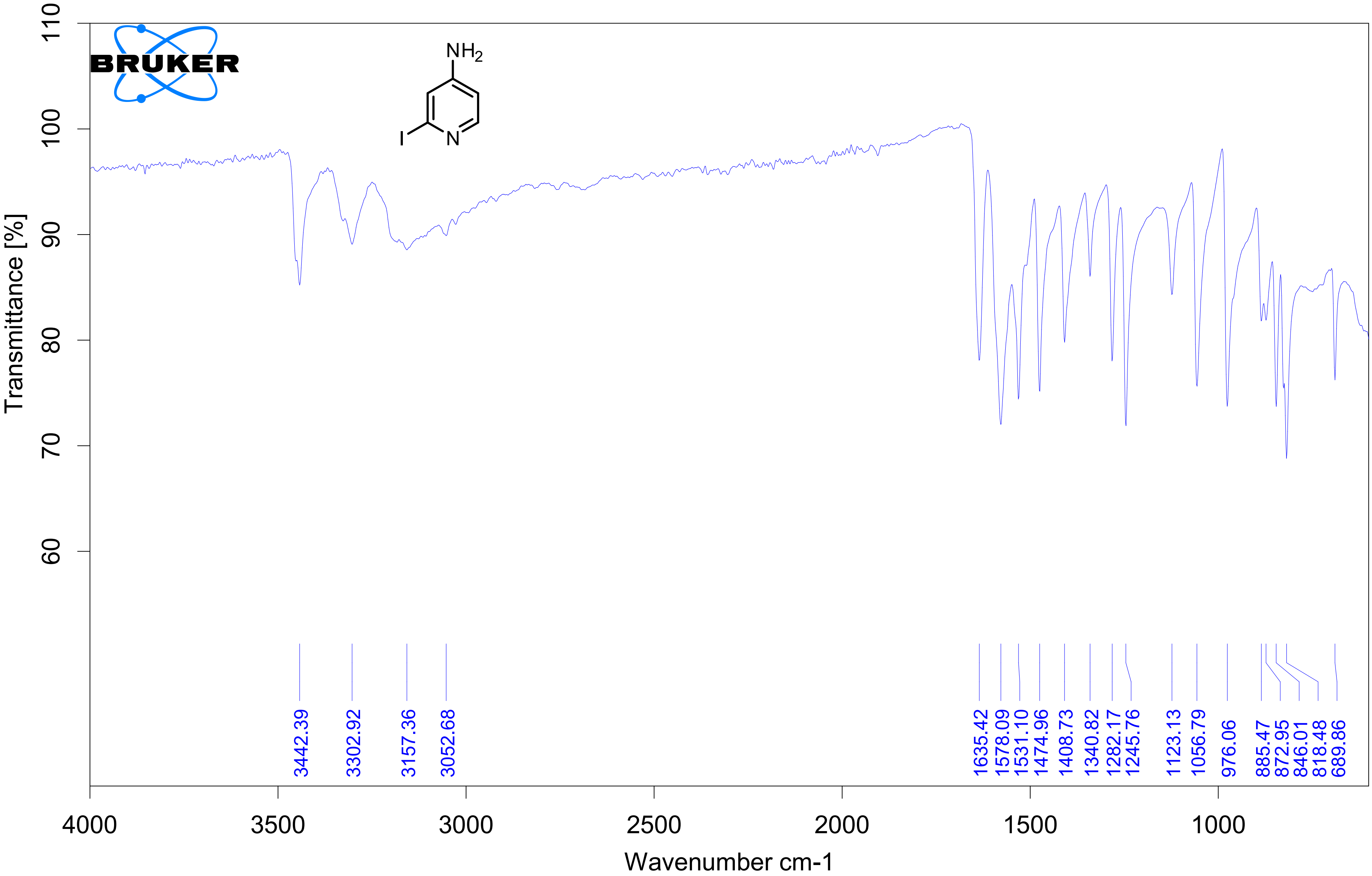

C:\DATA\User\Kim\KD-01-017.0	KD-01-017	oil	05.07.2021

ESI-MS

Analysis Info

Analysis Name	D:\Data\Kim\KD-01-017_1h3.d	Acquisition Date	29.04.2021 17:16:02
Method	AA_Standard_MS_2015.m		
Sample Name	KD-01-017_1h3	Instrument:	BRUKER - Ion Trap MS esquire HCT
Comment	in MeOH		

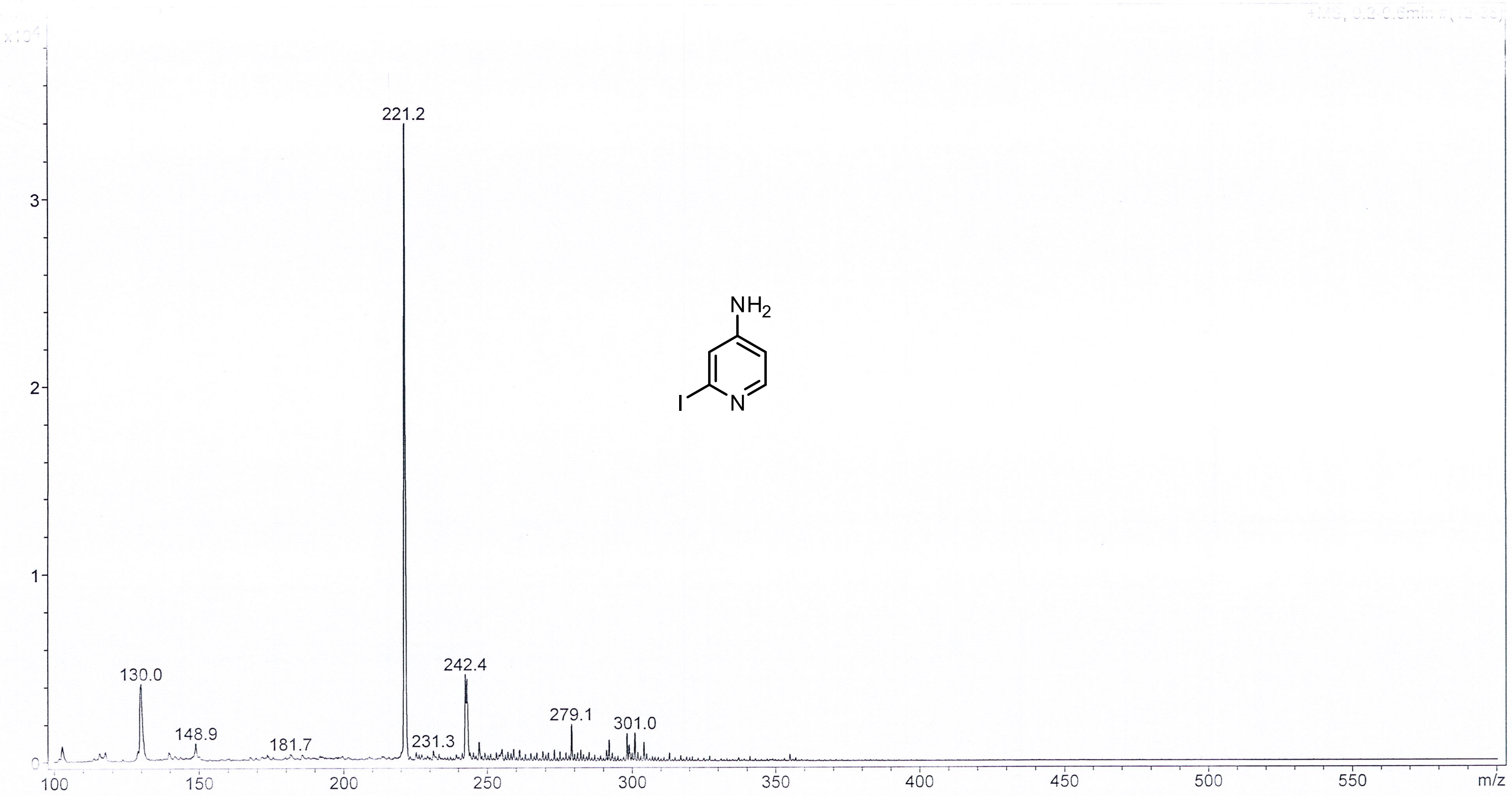

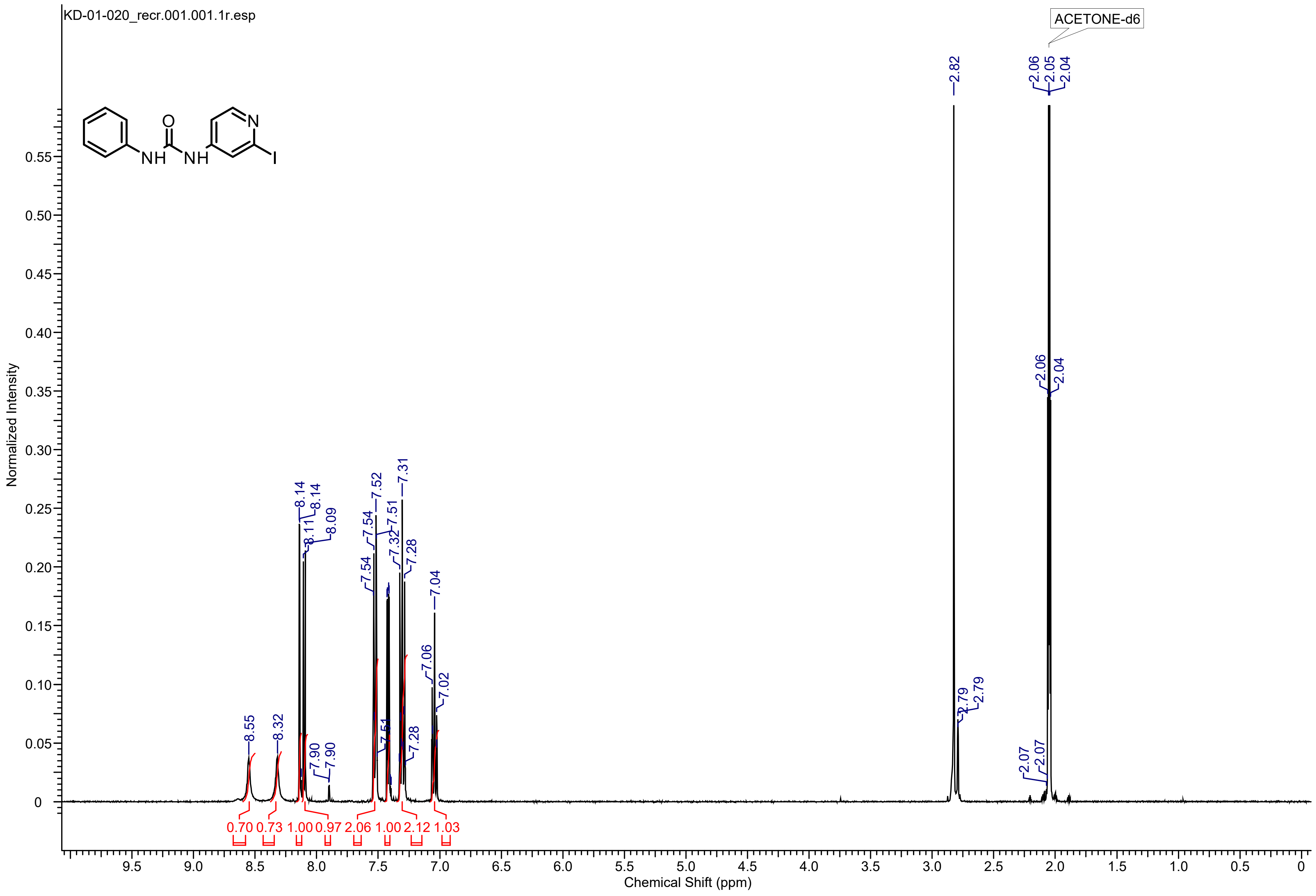
KD-01-020_recr.001.001.1r.esp
ACETONE-d6
Normalized Intensity
Chemical Shift (ppm)
8.55
8.32
8.14
8.14
8.11
8.09
7.90
7.90
7.54
7.54
7.52
7.51
7.32
7.31
7.28
7.28
7.06
7.04
7.02
2.82
2.79
2.79
2.07
2.07
2.06
2.05
2.04
0.70
0.73
1.00
0.97
2.06
1.00
2.12
1.03
O
NH
NH
N
I

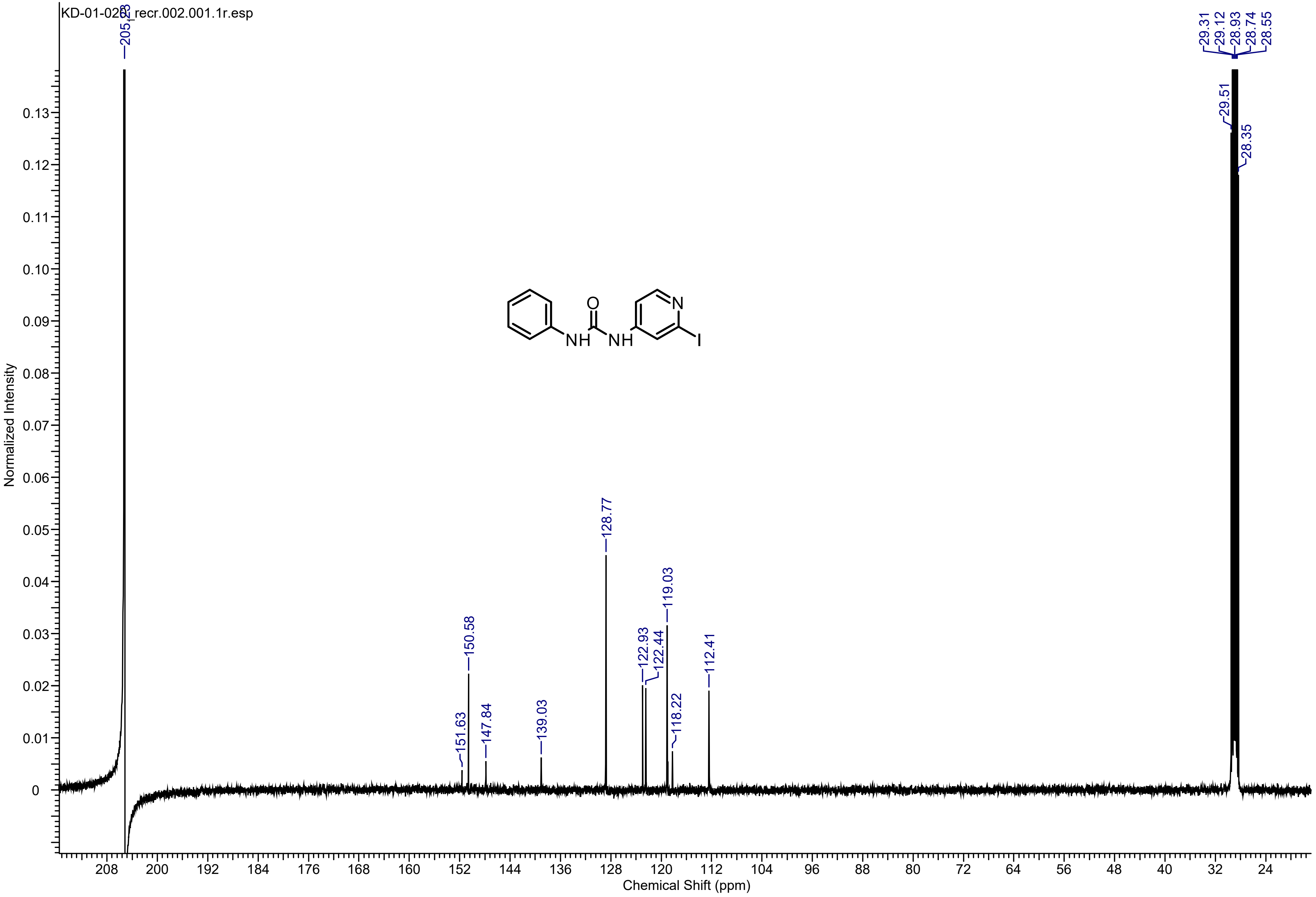
KD-01-020_recr.002.001.1r.esp
205.23
29.31
29.12
28.93
28.74
28.55
29.51
28.35
151.63
150.58
147.84
139.03
128.77
122.93
122.44
119.03
118.22
112.41
O
N
NH
NH
I
Normalized Intensity
0.13
0.12
0.11
0.10
0.09
0.08
0.07
0.06
0.05
0.04
0.03
0.02
0.01
0
208
200
192
184
176
168
160
152
144
136
128
120
112
104
96
88
80
72
64
56
48
40
32
24
Chemical Shift (ppm)

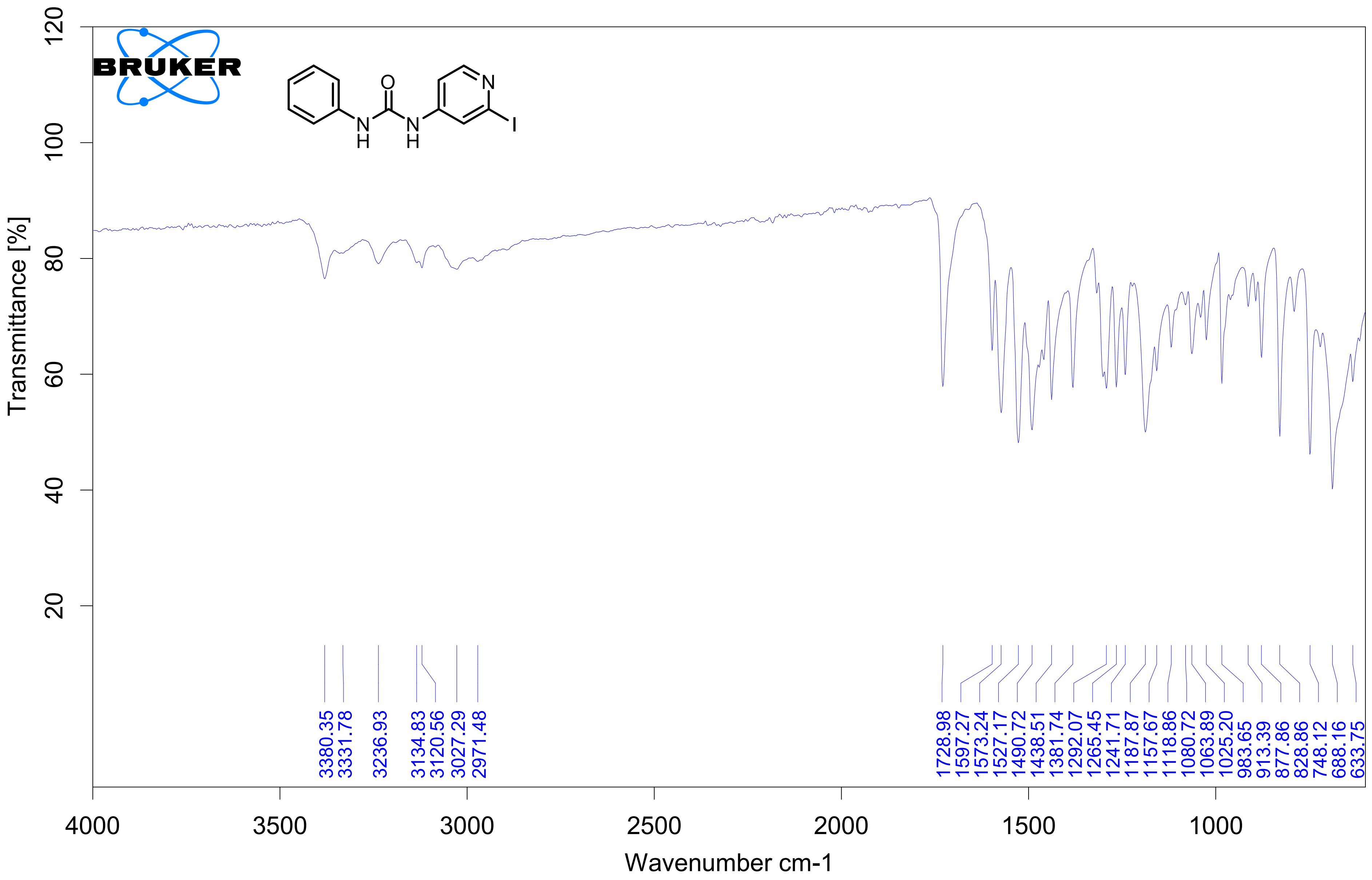

C:\DATA\User\Kim\KD-2I-analogue.0 KD-2I-analogue solid	05.07.2021

ESI-MS

Analysis Info | Acquisition Date | 19.05.2021 16:47:59

Analysis Name | D:\Data\Kim\2I-analogue_2.d
Method | AA_Standard_MS_2015.m
Sample Name | 2I-analogue_2 | Instrument: BRUKER - Ion Trap MS esquire HCT
Comment | in MeOH

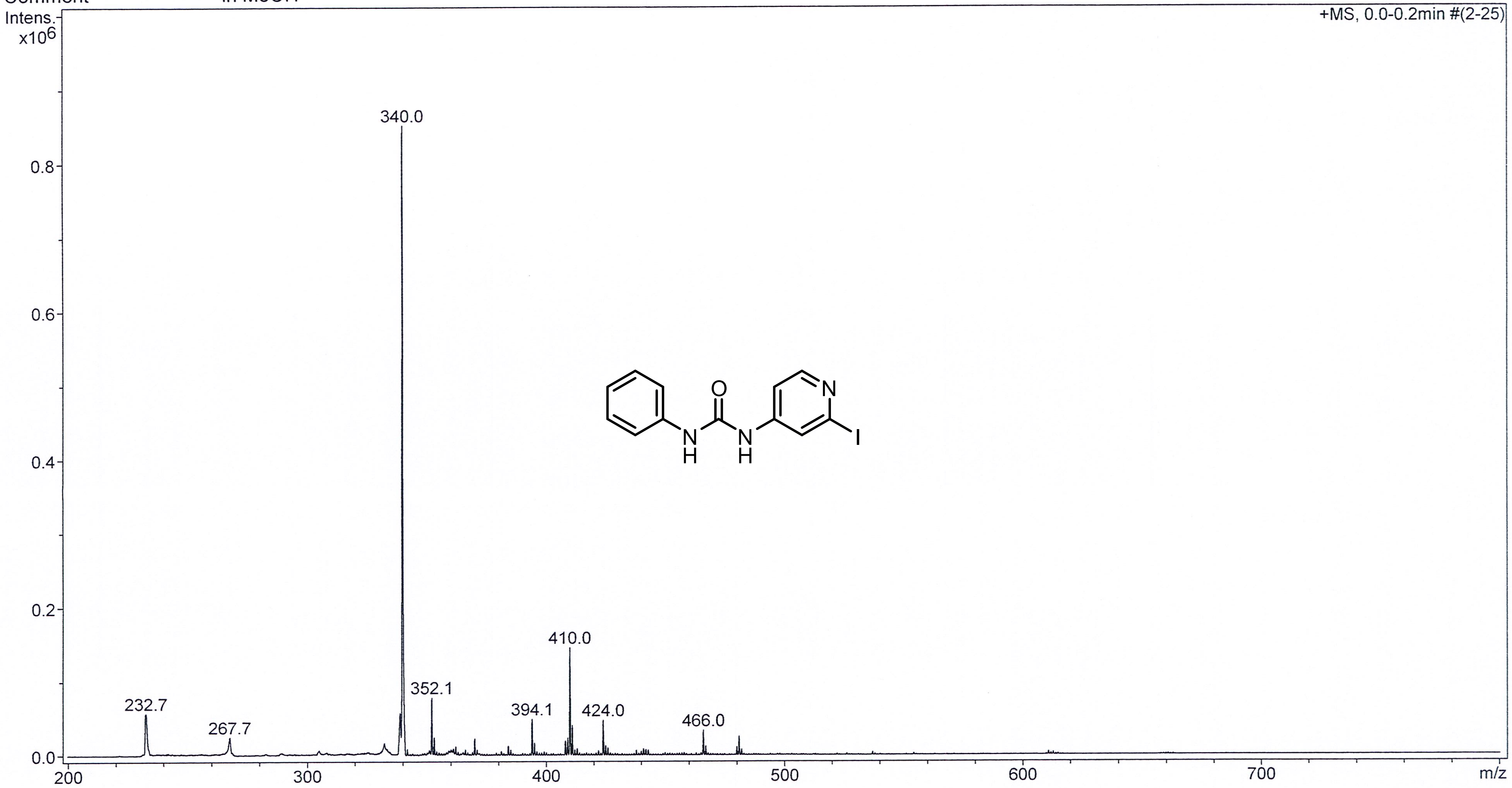

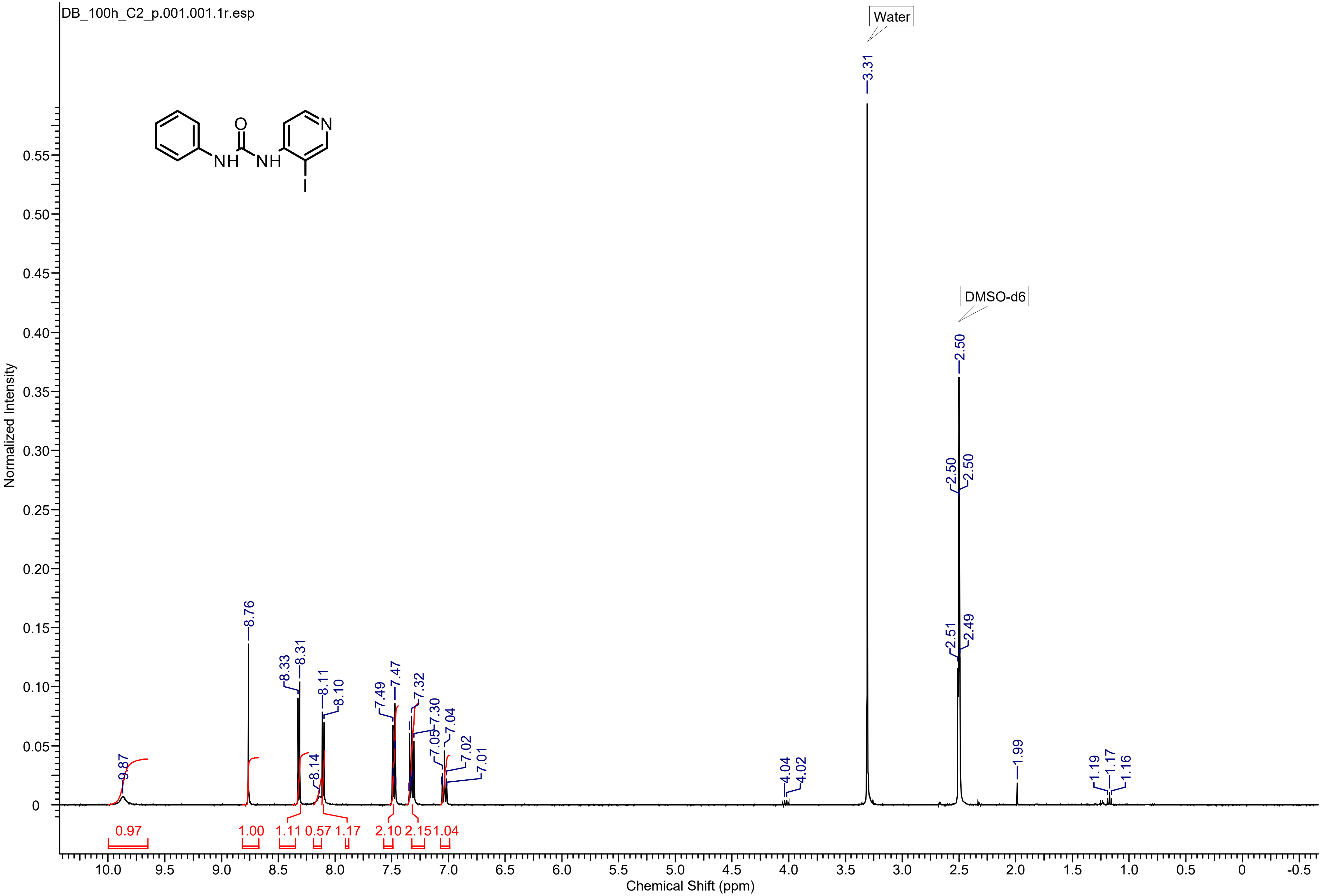
DB_100h_C2_p.001.001.1r.esp
Water
DMSO-d6
NH
NH
O
N
I
3.31
2.50
2.50
2.50
2.51
2.49
8.76
8.33
8.31
8.11
8.10
8.14
9.87
7.49
7.47
7.32
7.30
7.05
7.04
7.02
7.01
4.04
4.02
1.99
1.19
1.17
1.16
0.97
1.00
1.11
0.57
1.17
2.10
2.15
1.04
Normalized Intensity
Chemical Shift (ppm)

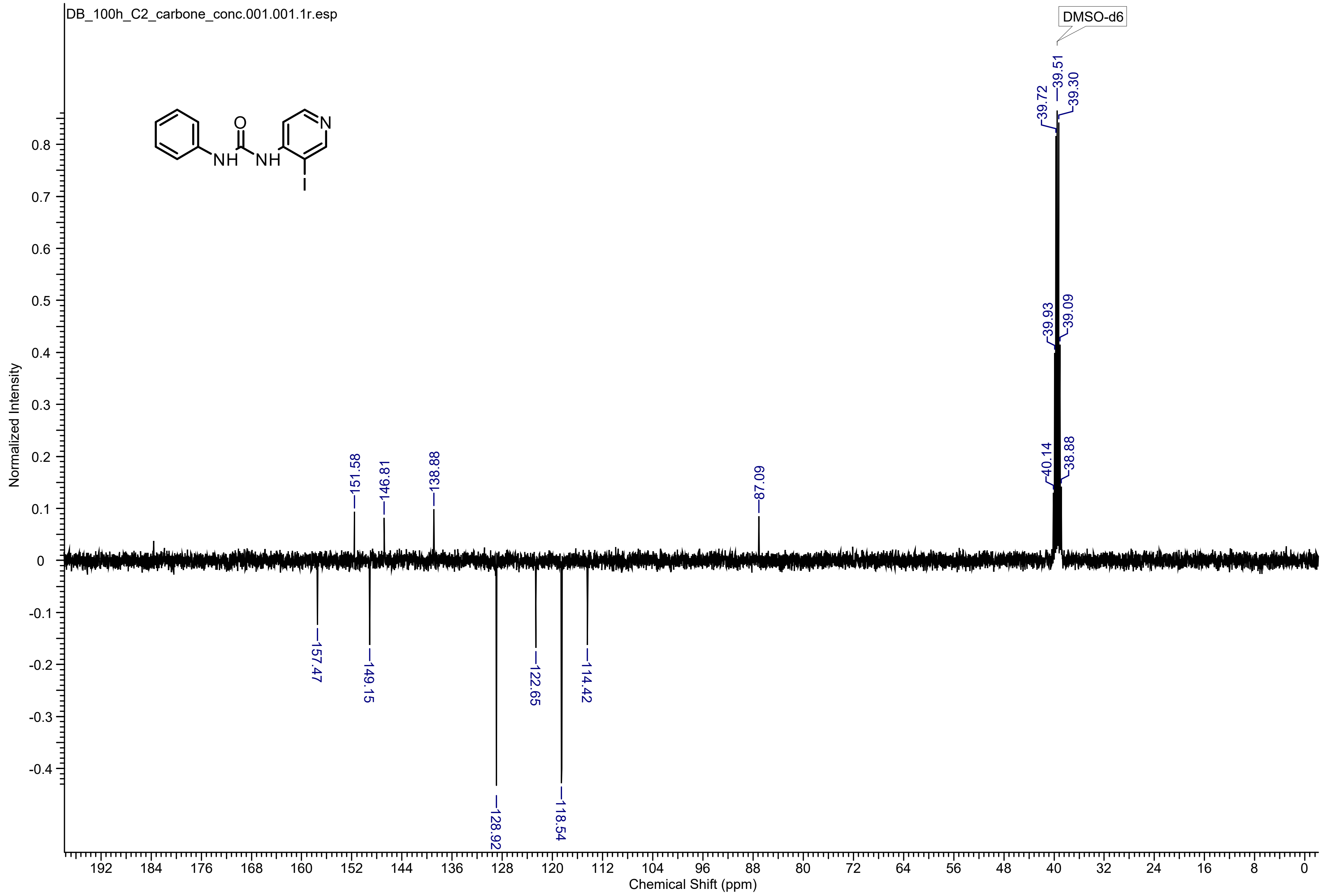
DB_100h_C2_carbone_conc.001.001.1r.esp
DMSO-d6
39.72
39.51
39.30
39.93
39.09
40.14
38.88
151.58
146.81
138.88
87.09
157.47
149.15
128.92
122.65
118.54
114.42
O
N
NH
NH
I
Normalized Intensity
Chemical Shift (ppm)

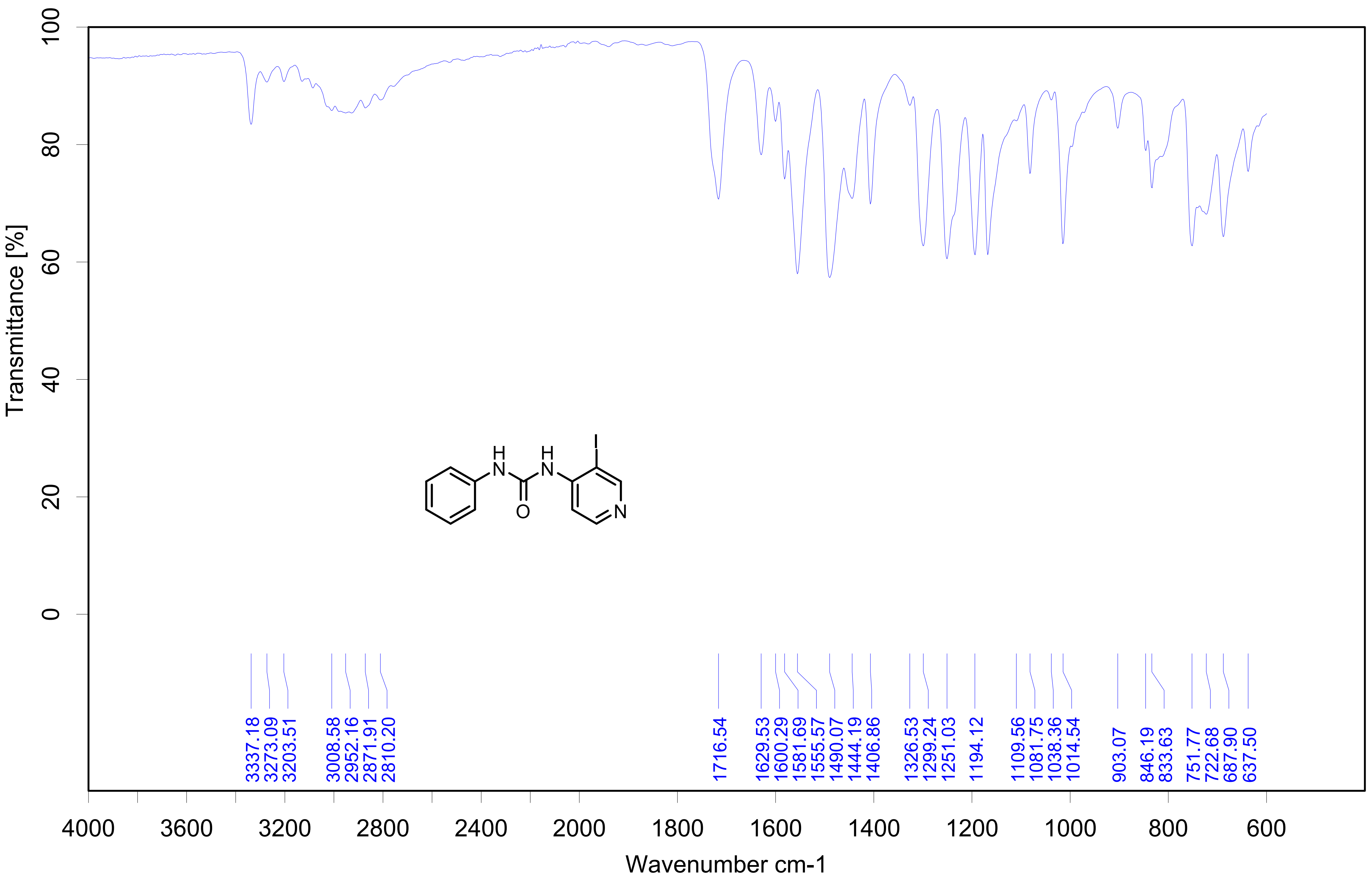

C:\DATA\User\Kim	C2.0	Date: 27.08.2019, 16:21:48

ESI-MS

Analysis Info

Analysis Name	D:\Data\Kim\FCF_02.d	Acquisition Date	05.07.2021 17:04:35
Method	AA_Standard_MS_2015.m		
Sample Name	FCF_02	Instrument:	BRUKER - Ion Trap MS esquire HCT
Comment	in MeOH		

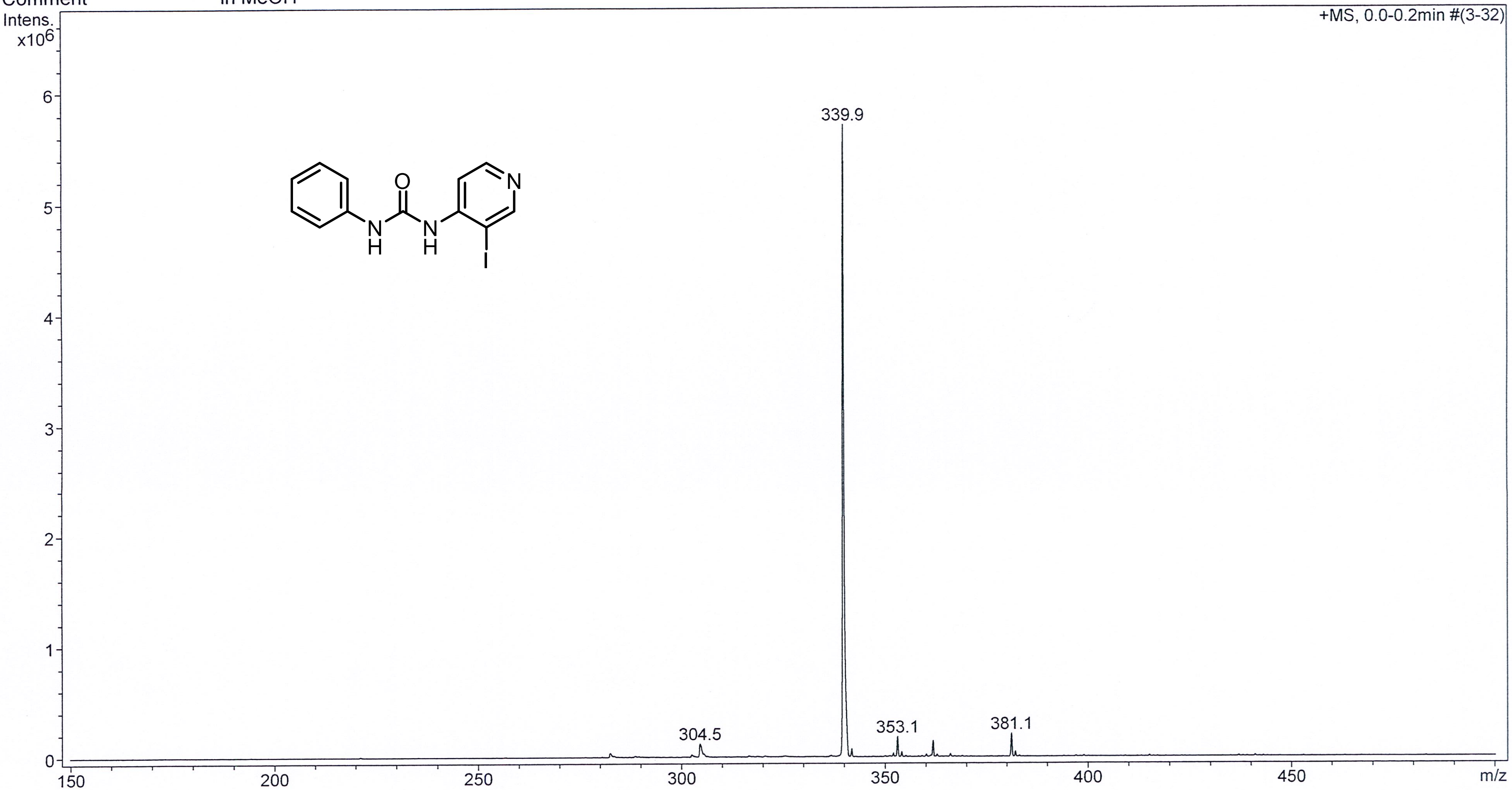

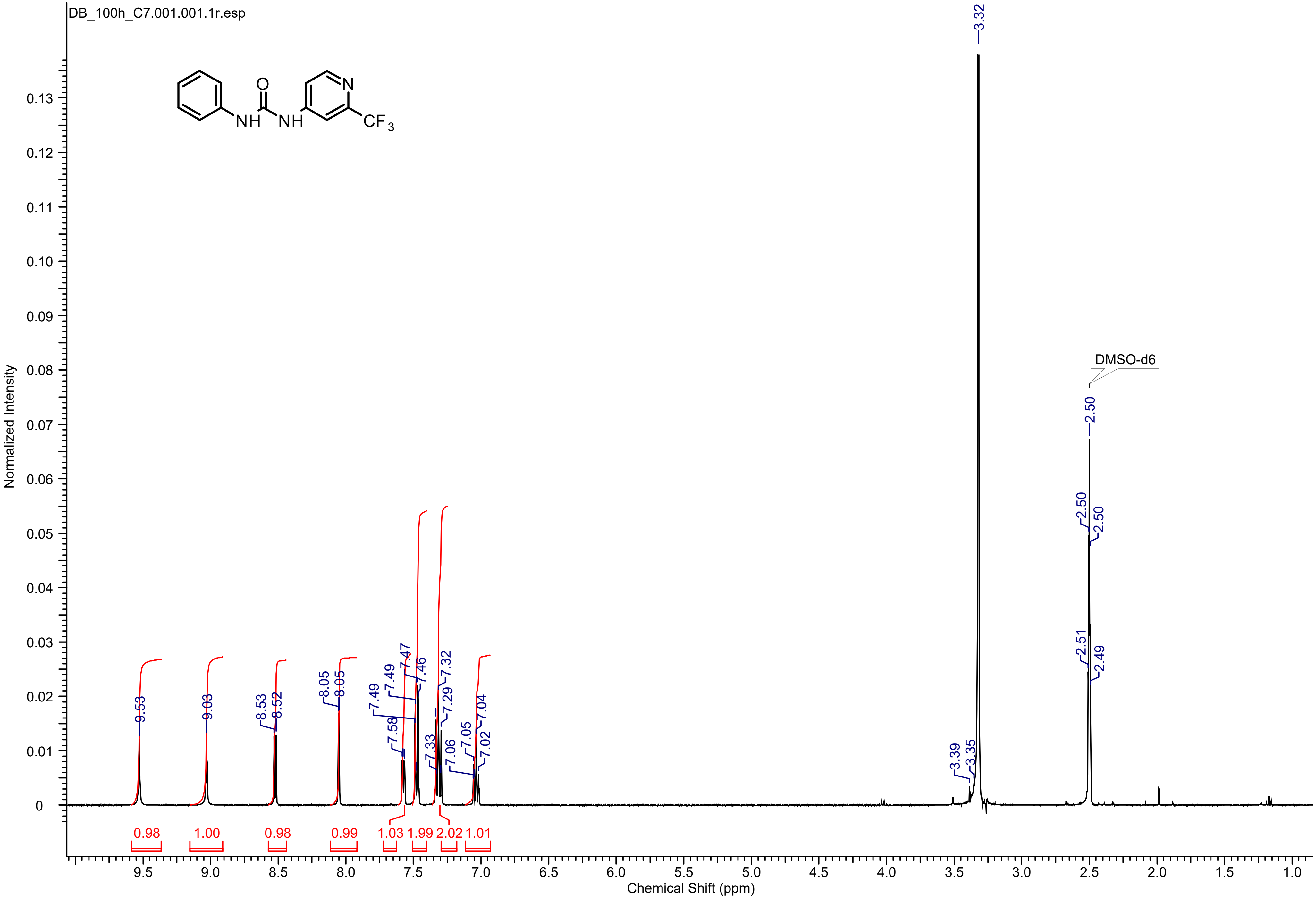

DB_100h_C7.001.001.1r.esp
NH
NH
O
N
CF3
DMSO-d6
Normalized Intensity
Chemical Shift (ppm)
9.53
9.03
8.53
8.52
8.05
8.05
7.58
7.49
7.49
7.47
7.46
7.33
7.32
7.29
7.06
7.05
7.04
7.02
3.39
3.35
3.32
2.51
2.50
2.50
2.50
2.49
0.98
1.00
0.98
0.99
1.03
1.99
2.02
1.01

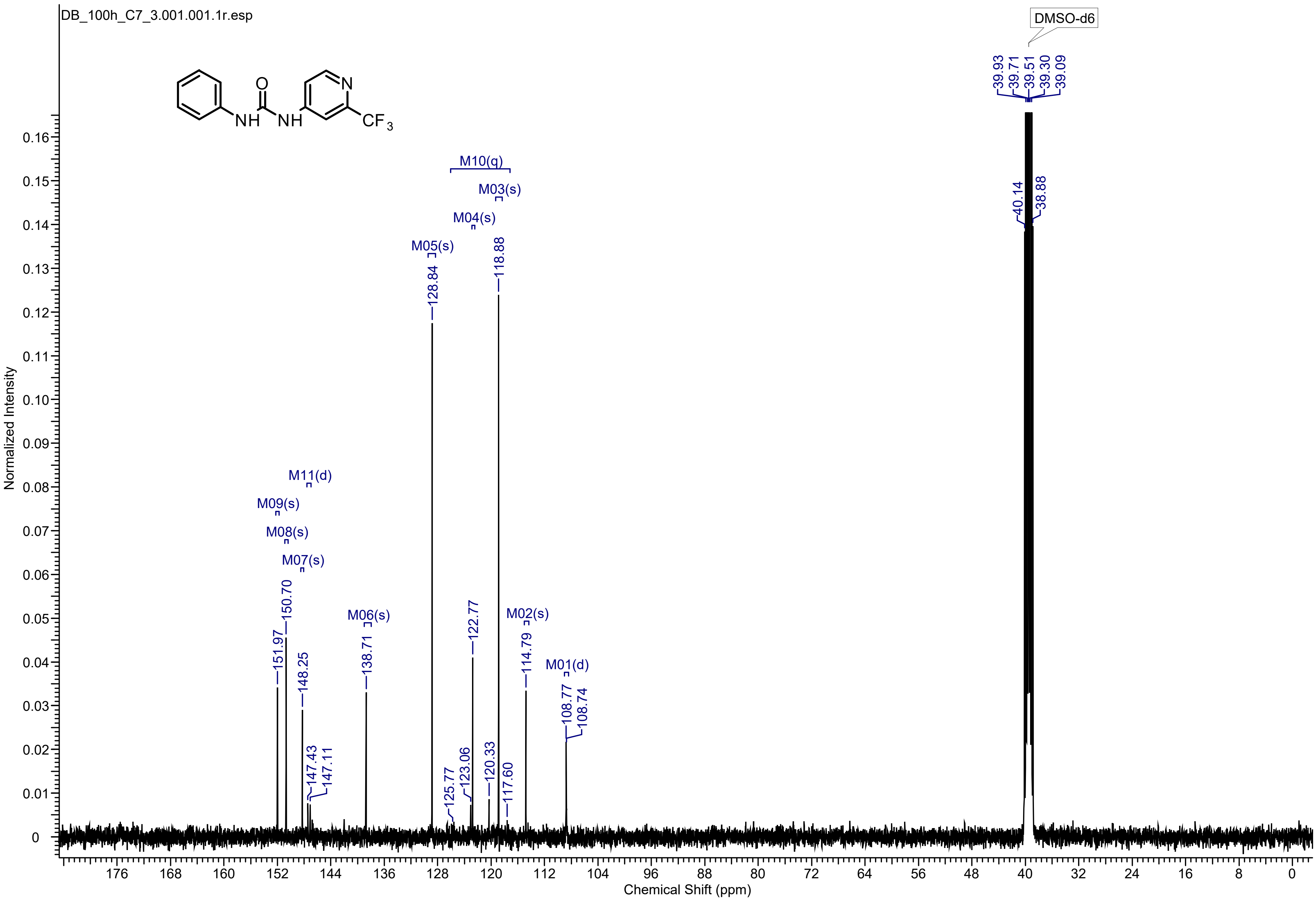
DB_100h_C7_3.001.001.1r.esp
DMSO-d6
NH
NH
O
N
CF3
39.93
39.71
39.51
39.30
39.09
40.14
38.88
M10(q)
M03(s)
M04(s)
M05(s)
128.84
118.88
M11(d)
M09(s)
M08(s)
M07(s)
150.70
151.97
148.25
147.43
147.11
M06(s)
138.71
122.77
125.77
123.06
120.33
117.60
M02(s)
114.79
M01(d)
108.77
108.74
Normalized Intensity
Chemical Shift (ppm)
0.16
0.15
0.14
0.13
0.12
0.11
0.10
0.09
0.08
0.07
0.06
0.05
0.04
0.03
0.02
0.01
0
176
168
160
152
144
136
128
120
112
104
96
88
80
72
64
56
48
40
32
24
16
8
0

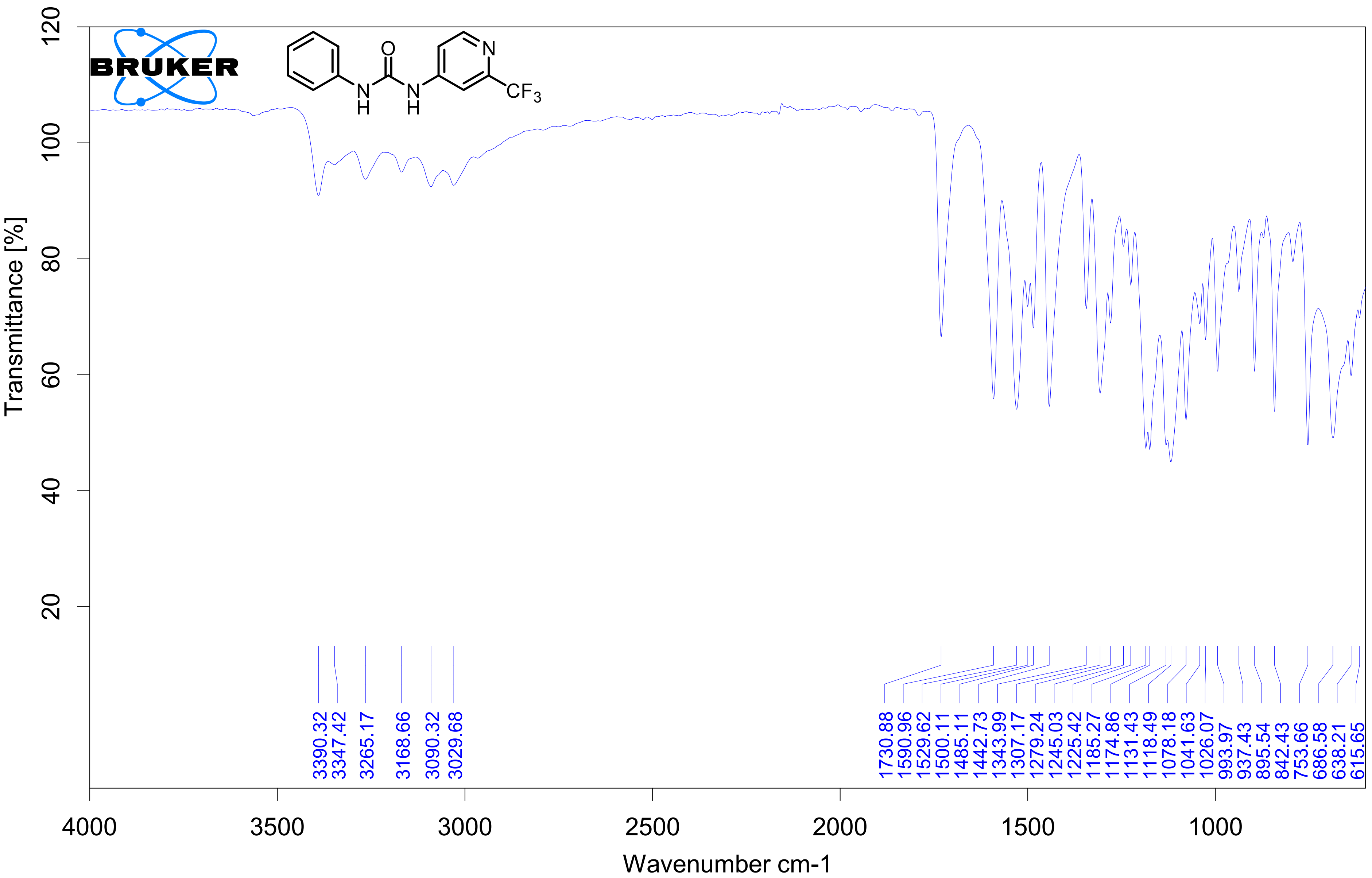

C:\DATA\User\Kim\C7.0 C7 solid	27.08.2019

ESI-MS

Analysis Info | Acquisition Date 05.07.2021 17:12:46

Analysis Name	D:\Data\Kim\FCF_07.d
Method	AA_Standard_MS_2015.m
Sample Name	FCF_07
Comment	in MeOH

Instrument: BRUKER - Ion Trap MS esquire HCT

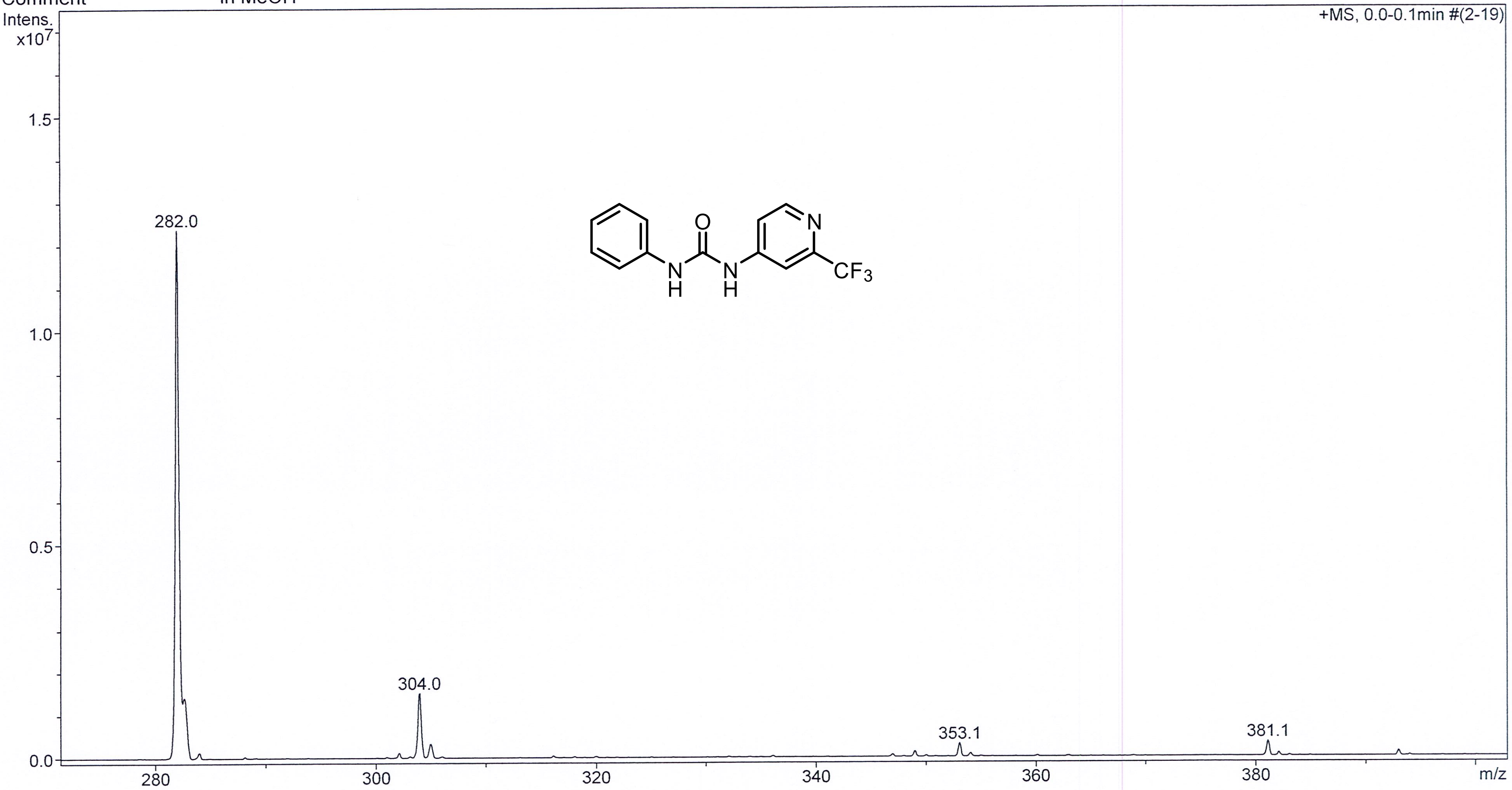

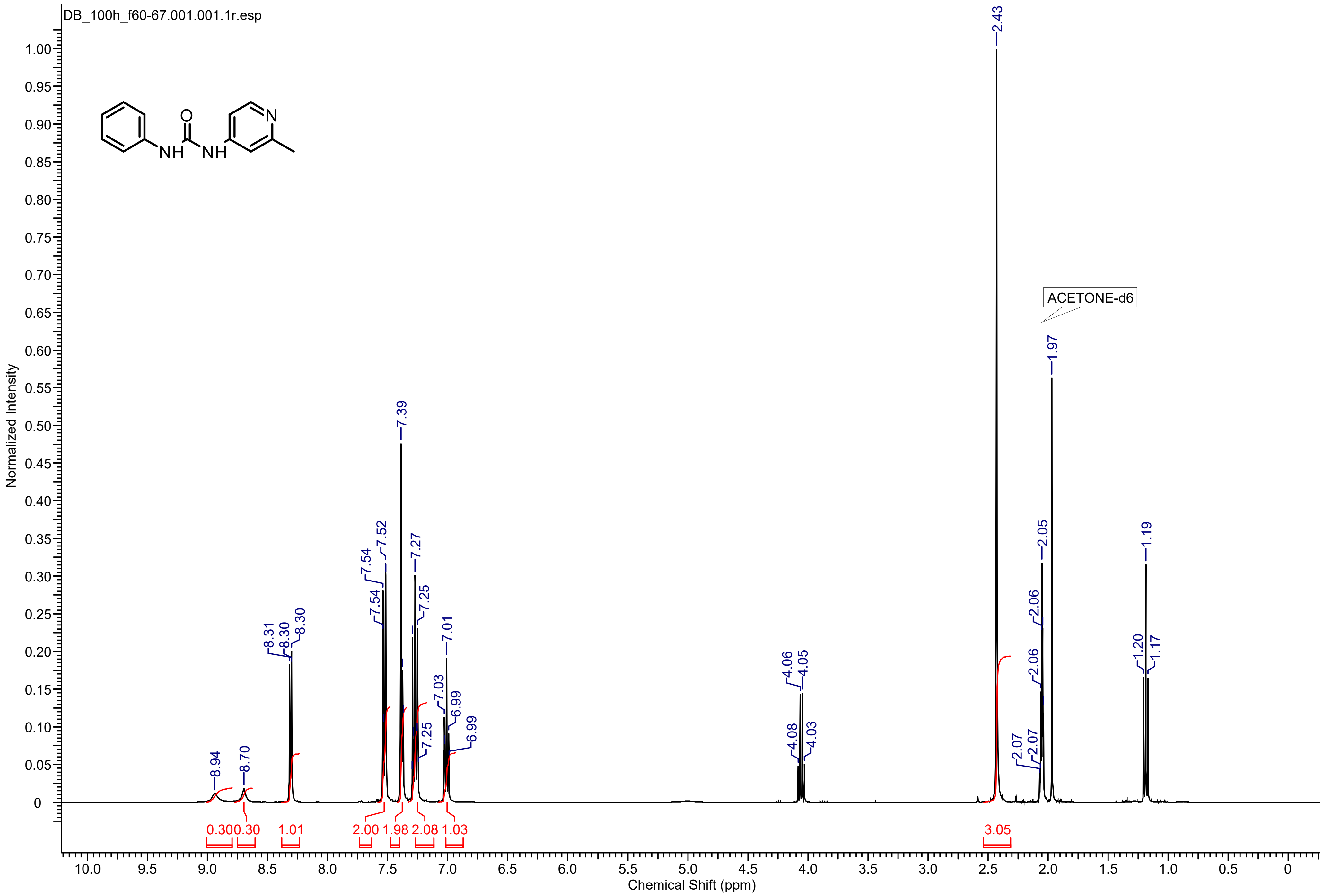
DB_100h_f60-67.001.001.1r.esp
ACETONE-d6
Normalized Intensity
Chemical Shift (ppm)
8.94
8.70
8.31
8.30
8.30
7.54
7.54
7.52
7.39
7.27
7.25
7.25
7.03
7.01
6.99
6.99
4.08
4.06
4.05
4.03
2.43
2.07
2.07
2.06
2.06
2.05
1.97
1.20
1.19
1.17
0.30
0.30
1.01
2.00
1.98
2.08
1.03
3.05

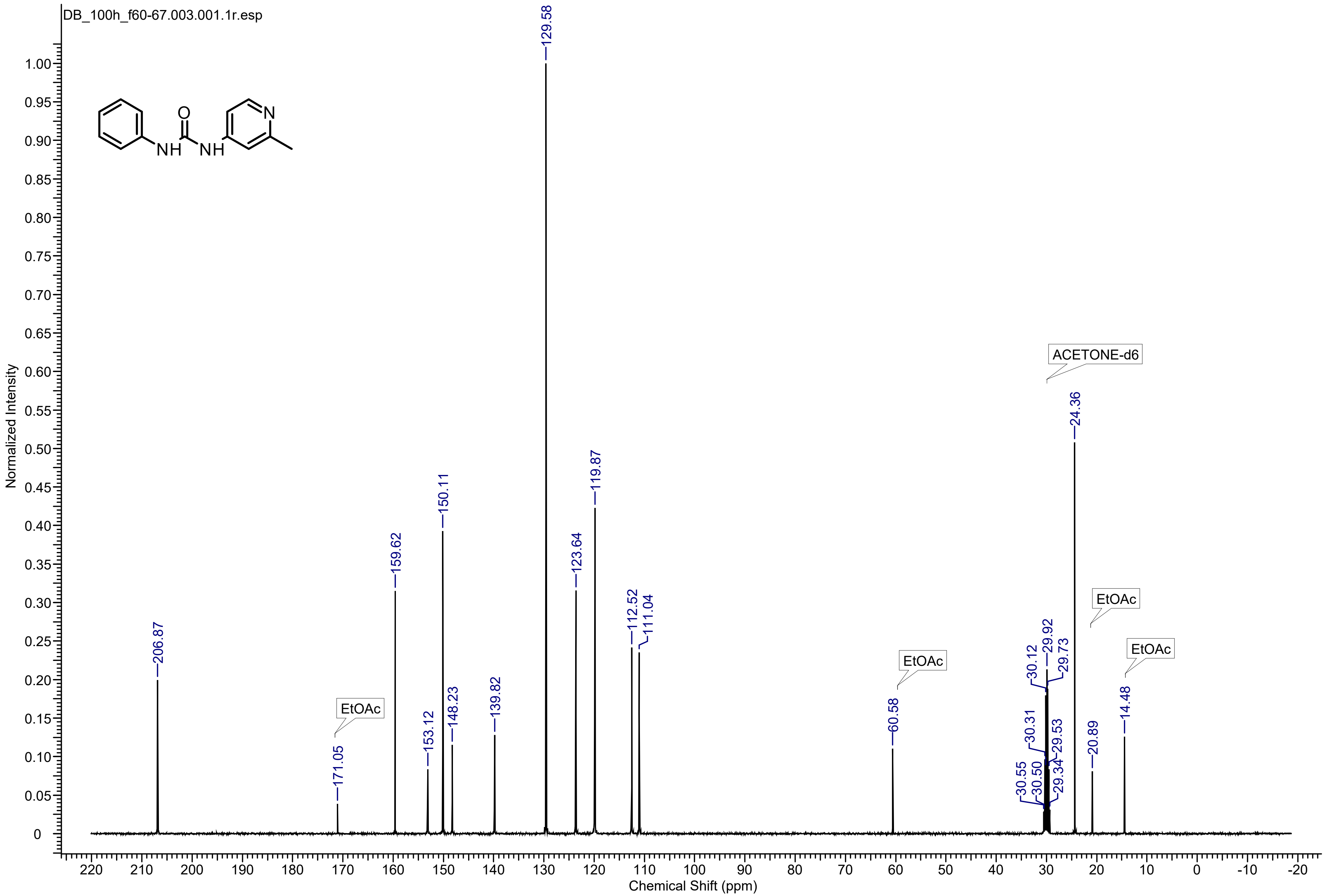

DB_100h_f60-67.003.001.1r.esp
O
NH
NH
N
206.87
171.05
EtOAc
159.62
153.12
150.11
148.23
139.82
129.58
123.64
119.87
112.52
111.04
60.58
EtOAc
30.55
30.50
30.31
30.12
29.92
29.73
29.53
29.34
ACETONE-d6
24.36
20.89
EtOAc
14.48
EtOAc
Normalized Intensity
Chemical Shift (ppm)

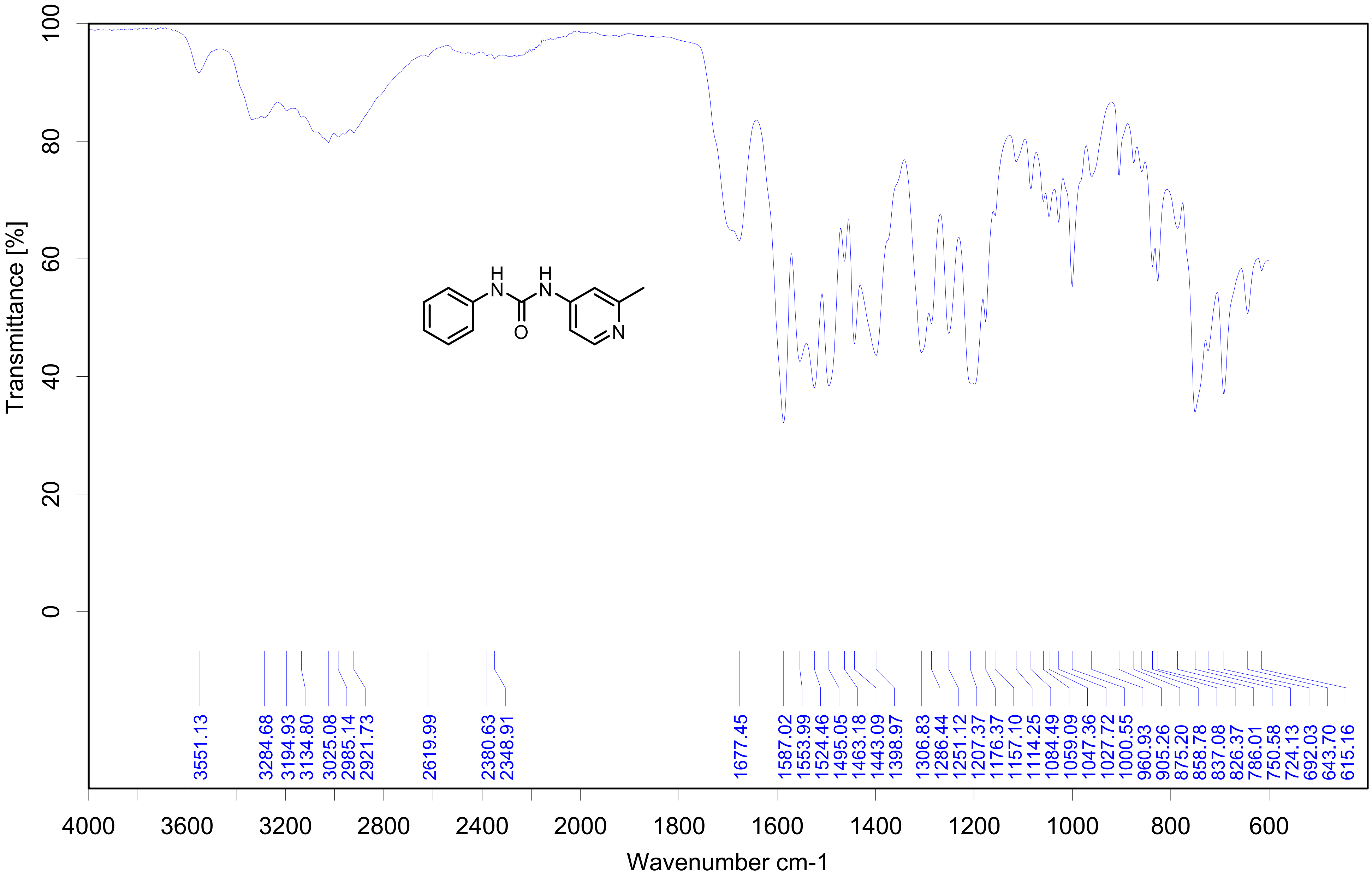

C:\DATA\User\Kim	C5.0	Date: 27.08.2019, 16:42:05

ESI-MS

Analysis Info Acquisition Date 05.07.2021 17:10:40

Analysis Name D:\Data\Kim\FCF_05.d
Method AA_Standard_MS_2015.m
Sample Name FCF_05 Instrument: BRUKER - Ion Trap MS esquire HCT
Comment in MeOH

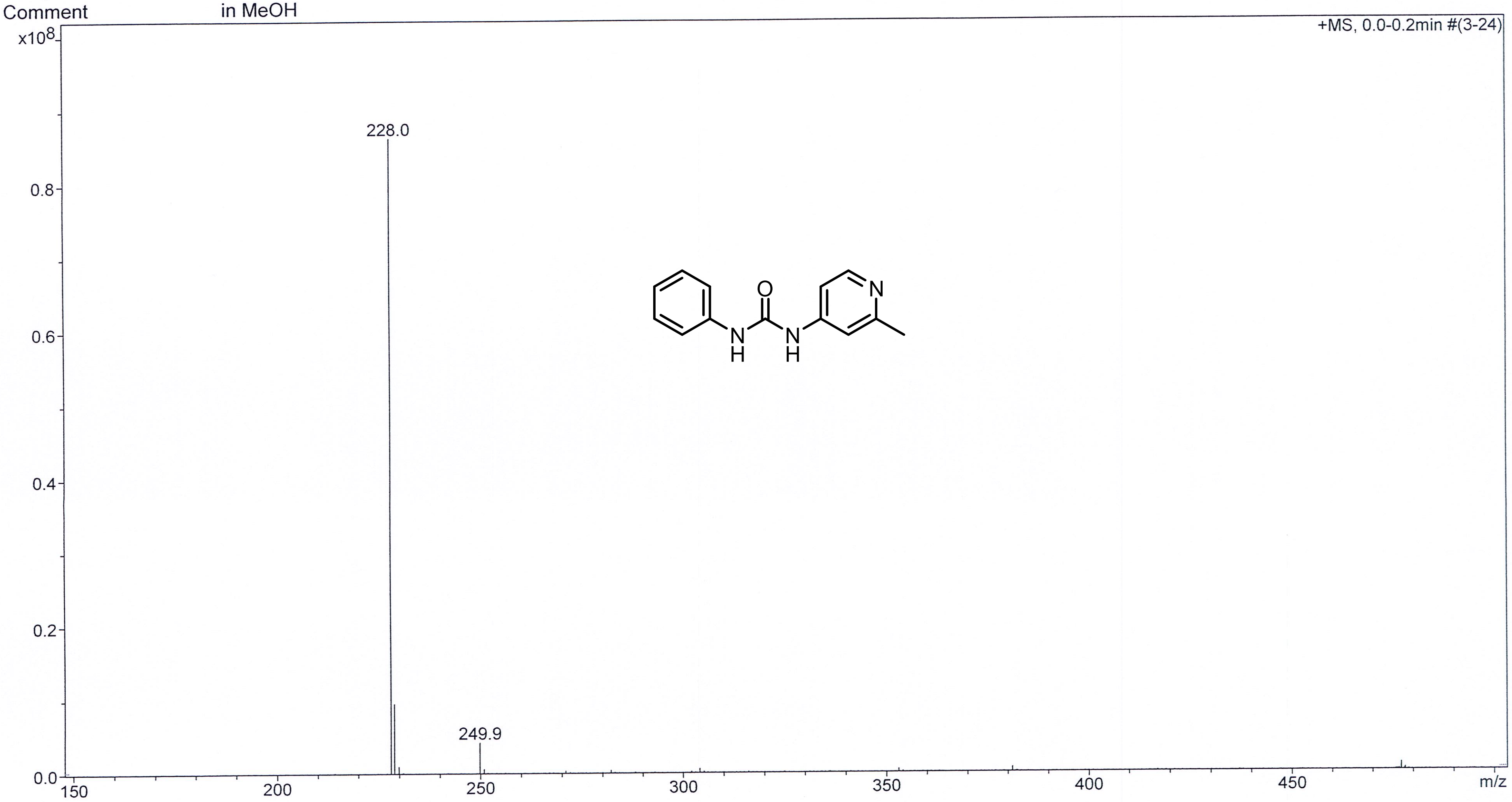

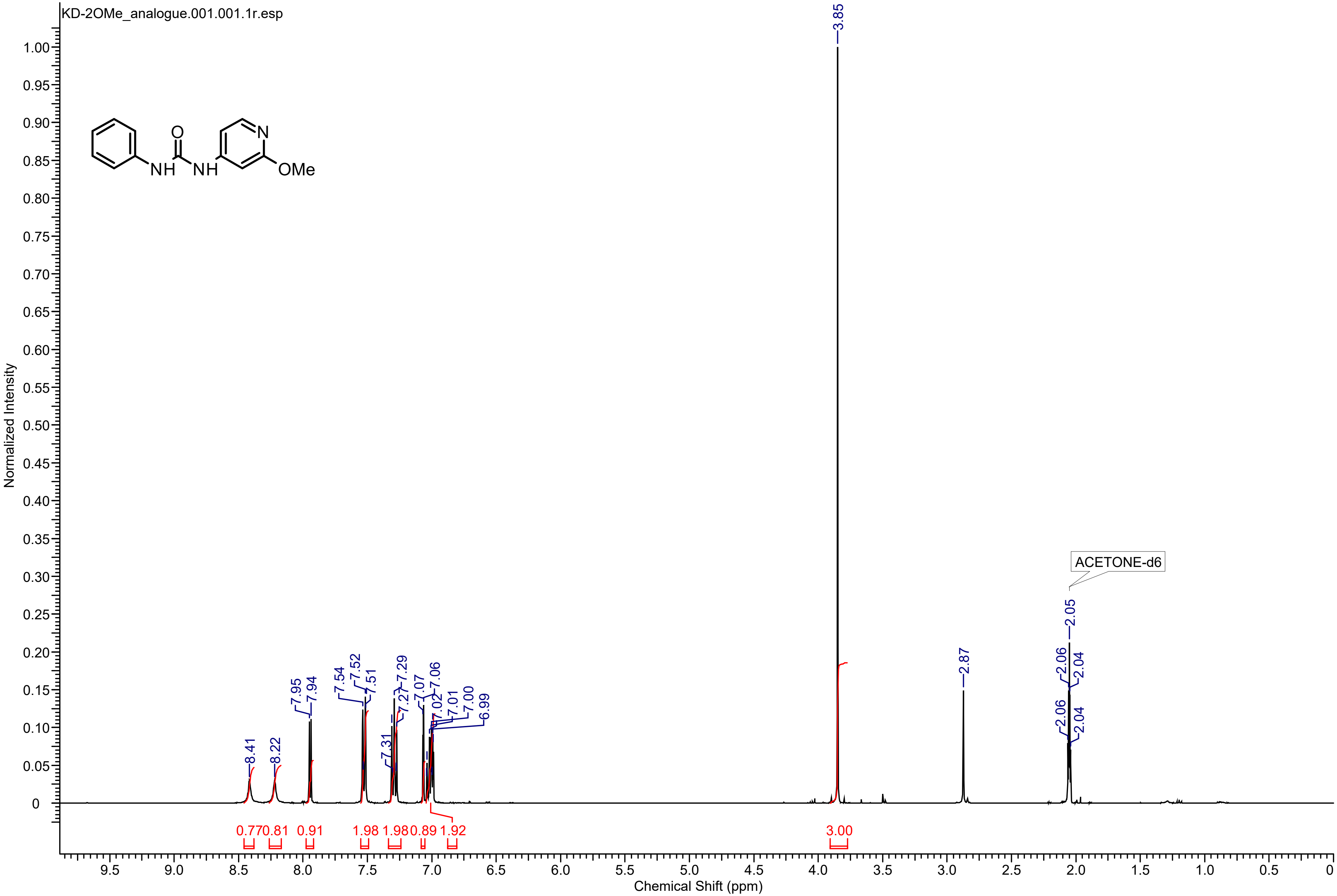

KD-2OMe_analogue.001.001.1r.esp
NH
NH
O
N
OMe
ACETONE-d6
3.85
2.87
2.06
2.05
2.04
8.41
8.22
7.95
7.94
7.54
7.52
7.51
7.31
7.29
7.27
7.07
7.06
7.02
7.01
7.00
6.99
0.77
0.81
0.91
1.98
1.98
0.89
1.92
3.00
Normalized Intensity
Chemical Shift (ppm)

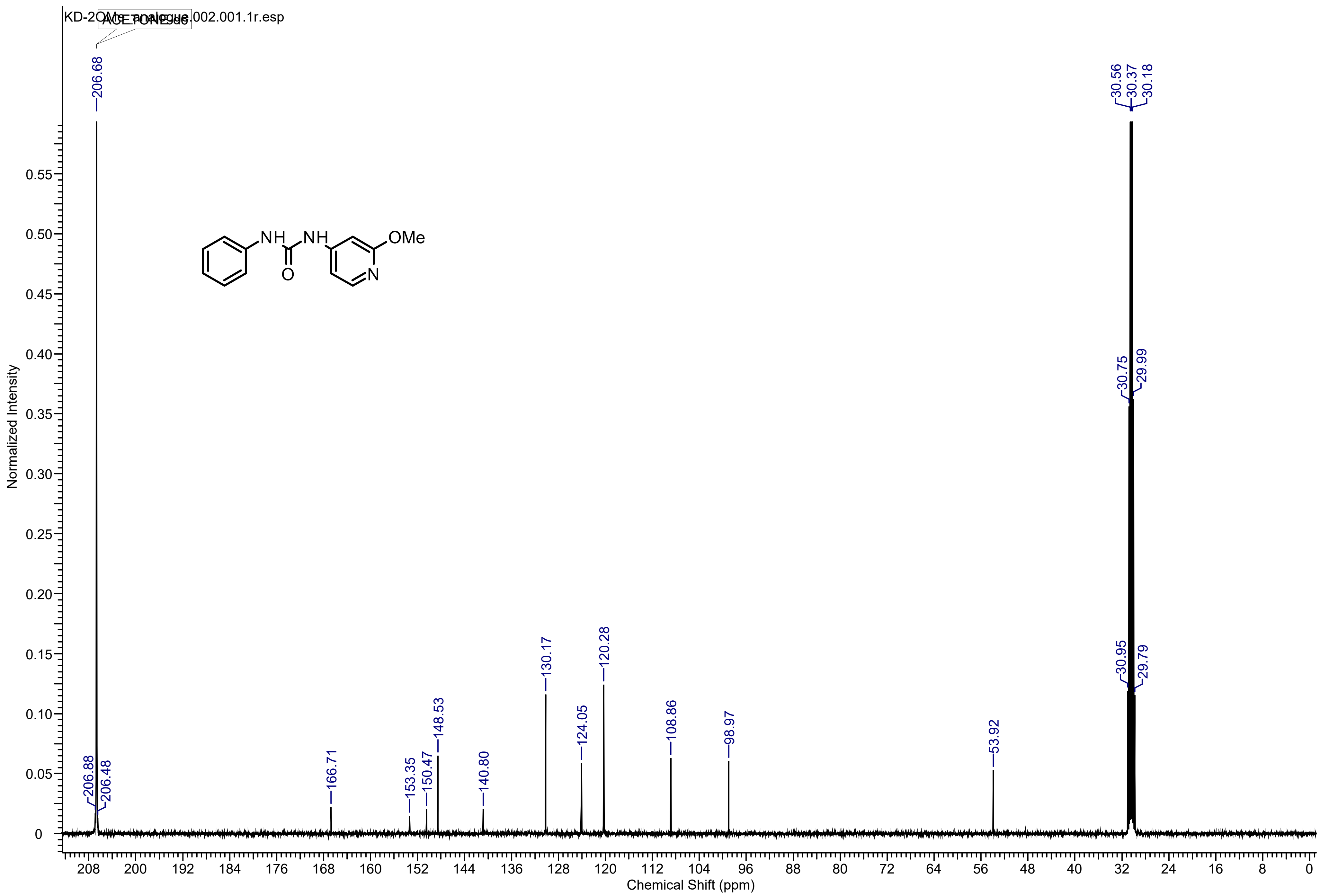

KD-2OMe analogue.002.001.1r.esp
ACETONE-d6
206.88
206.68
206.48
166.71
153.35
150.47
148.53
140.80
130.17
124.05
120.28
108.86
98.97
53.92
30.95
30.75
30.56
30.37
30.18
29.99
29.79
NH
NH
O
OMe
N
Normalized Intensity
Chemical Shift (ppm)

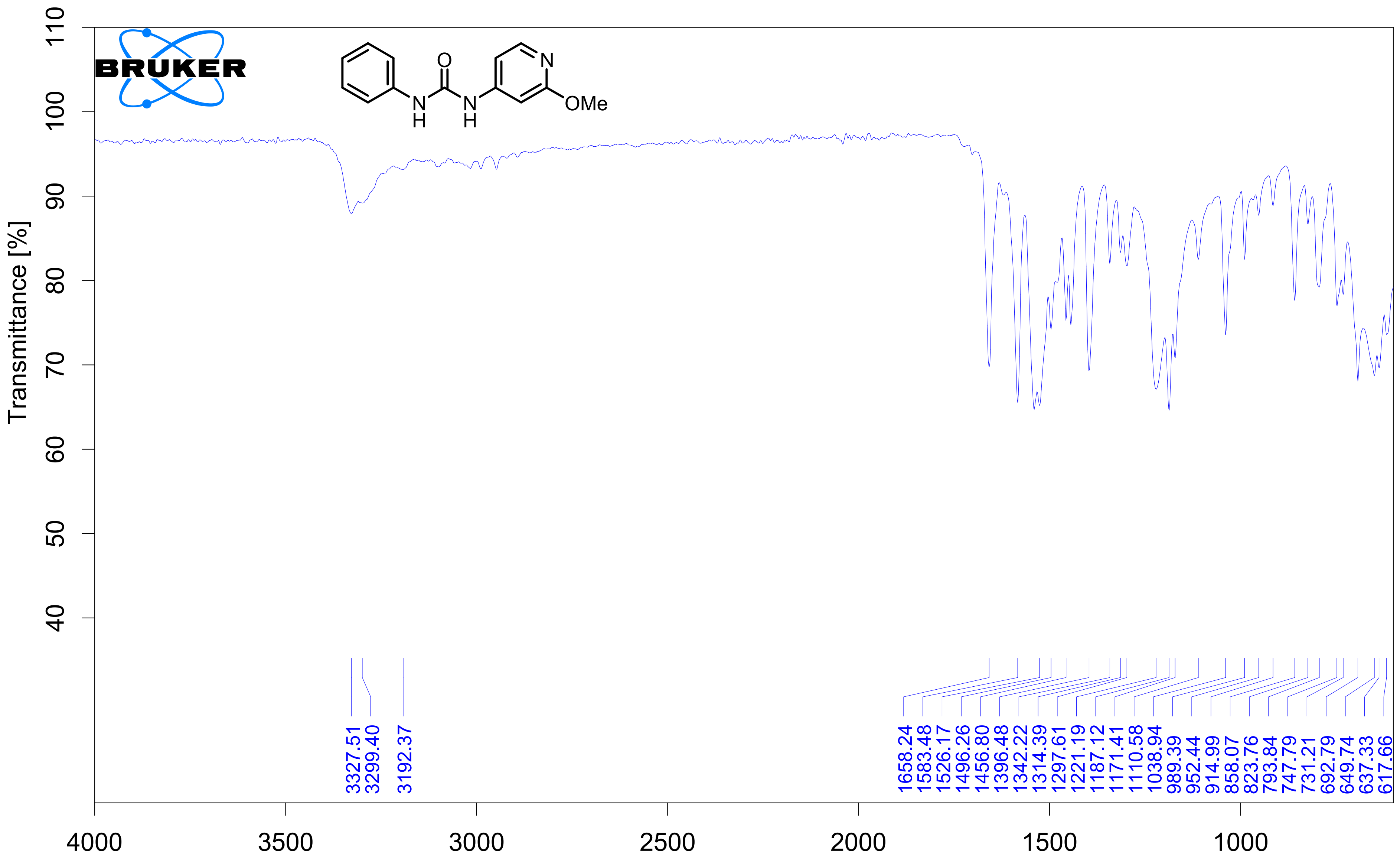

C:\DATA\User\Kim\KD-2OMe-analogue.0	KD-2OMe-analogue	solid	05.07.2021

ESI-MS

Analysis Info

Acquisition Date 05.07.2021 17:18:21

Analysis Name D:\Data\Kim\FCF_10.d
Method AA_Standard_MS_2015.m
Sample Name FCF_10
Comment in MeOH

Instrument: BRUKER - Ion Trap MS esquire HCT

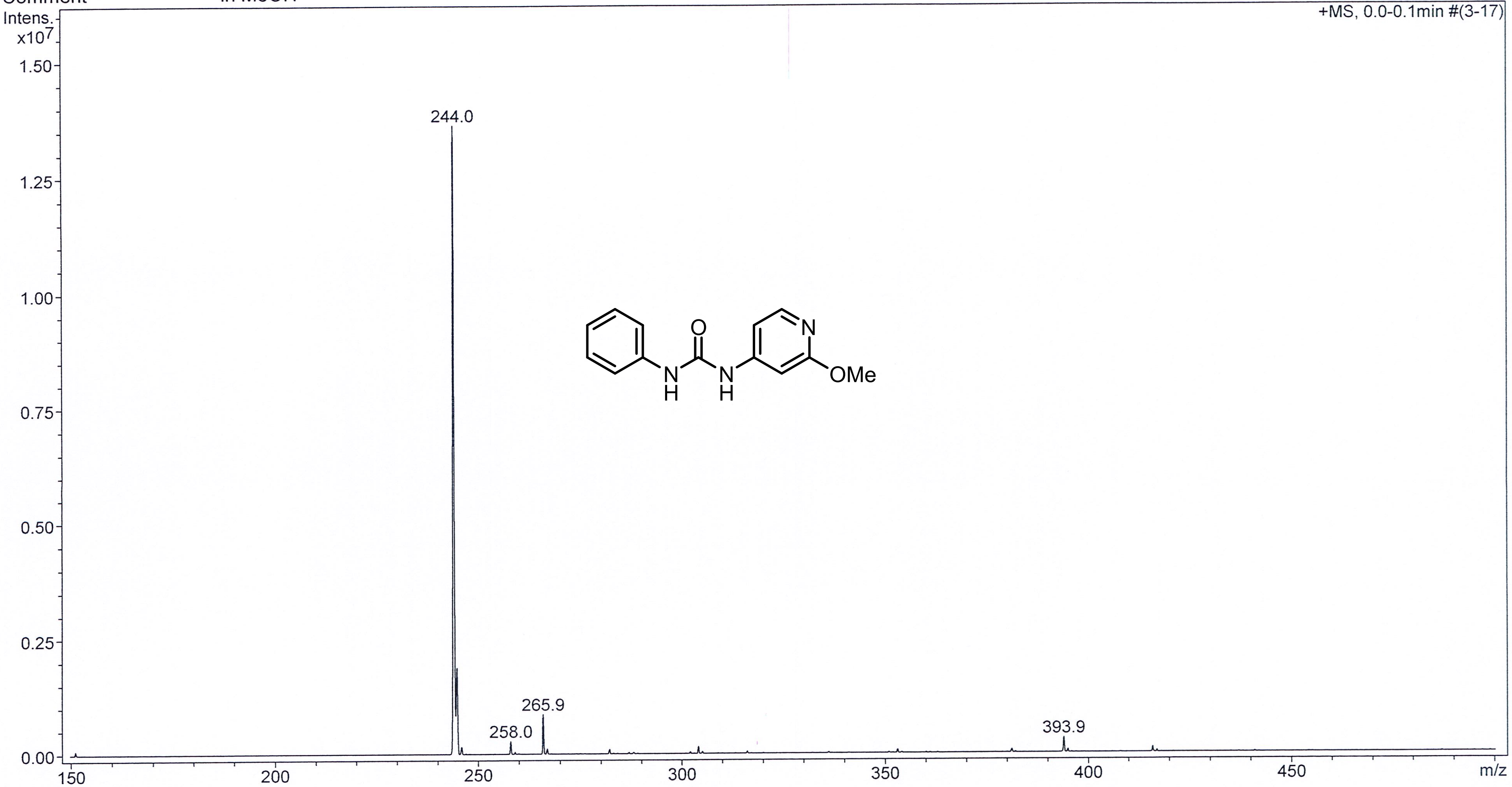

Supplement: Supplementary file 1 [file ijms-23-03963-s001.zip › ijms-1629384-supplementary.pdf]
